# A Survey on Current Practices, Needs, Responsibilities and Preferences for Knowledge Dissemination in the Field of Injury and Illness Prevention Among Competitive Snow Sports Stakeholders

**DOI:** 10.1186/s40798-025-00818-9

**Published:** 2025-02-19

**Authors:** Oriol Bonell Monsonís, Jörg Spörri, Vincent Gouttebarge, Caroline Bolling, Evert Verhagen

**Affiliations:** 1 Amsterdam Collaboration on Health and Safety in Sports, Department of Public and Occupational Health, Amsterdam Movement Sciences, Amsterdam UMC, University Medical Centres – Vrije Universiteit Amsterdam, Amsterdam, The Netherlands; 2 Amsterdam Movement Sciences, Musculoskeletal Health and Sports, Amsterdam, The Netherlands; 3 Sports Medical Research Group, Department of Orthopaedics, Balgrist University Hospital, University of Zurich, Zurich, Switzerland; 4 University Centre for Prevention and Sports Medicine, Department of Orthopaedics, Balgrist University Hospital, University of Zurich, Zurich, Switzerland; 5 Department of Orthopaedic Surgery and Sports Medicine, Amsterdam UMC Location University of Amsterdam, Amsterdam, The Netherlands; 6 Section Sports Medicine, Faculty of Health Sciences, University of Pretoria, Pretoria, South Africa

**Keywords:** Competitive snow sports, Injury, Illness, Prevention, Practices, Needs, Dissemination

## Abstract

**Background:**

Injury and illness prevention practices in competitive snow sports must be better understood among stakeholders. In particular, there is a need for a greater understanding of what context-specific stakeholders require for prevention. Therefore, this study surveyed stakeholders’ current practices, needs, responsibilities and knowledge dissemination preferences related to injury and illness prevention in competitive snow sports and described the main commonalities and differences between stakeholder groups.

**Methods:**

We conducted a cross-sectional study that used an online survey developed using Kipling’s principle (the “5W1H” method) and targeted athletes, coaches, team staff, ski racing suppliers, and representatives from all competition levels and all competitive snow sports governed by the International Ski and Snowboard Federation. The data were analysed following both quantitative and qualitative descriptive analyses.

**Results:**

Most of the 436 respondents believed in and reported needing more information on injury and illness prevention. The participants stated that the main goal of prevention was to avoid injuries and minimise their time away from being on snow, and they stressed their different underlying motivations. Despite the differences across subgroups, participants highlighted knee and head injuries and concussions as their primary injury prevention targets and priorities for additional information. Respiratory and cardiovascular illnesses were reported as their main targets of illness prevention, but more information on all illnesses was reported. Current practices and priorities for additional information fell under athlete-, equipment-, snow/environment-, and course-related prevention areas. Moreover, stakeholders highlighted their need for more information on mental health and training. Shared responsibilities were identified across the development, dissemination, and implementation of prevention, along with stakeholders’ preferred communication channels.

**Conclusions:**

Our study provides meaningful insights across athlete, equipment-, snow/environment-, and course-related prevention areas related to snow sports, roles, and competition levels. These insights may inform the development, dissemination and further implementation of any tailored and context-driven preventive measure by better addressing end-users’ needs. These findings may support successful future preventive interventions by providing key elements and a clear path to improve athletes’ health and safety.

**Supplementary Information:**

The online version contains supplementary material available at 10.1186/s40798-025-00818-9.

## Key Points

- In addition to the overview provided regarding injury and illness prevention within competitive snow sports, differences in practices and priorities between snow sports, roles and competition levels were identified, mainly because of the nature and characteristics of their settings.
- These differences in current practices and needs priorities between stakeholder groups indicate the need for context-specific and tailored approaches across athletes, equipment, course settings, and snow domains. Mental health and mental training were deemed the domains with the highest priority for further knowledge across all prevention areas.
- Different stakeholders play different roles in prevention, with athletes and coaches at the centre in the development and implementation. Collective approaches involving key stakeholders in developing, disseminating, and implementing prevention measures highlight the significance of shared responsibilities and stakeholder cooperation.
- Newly emerging topics included mental health and performance, the health and performance of female athletes, gradual onset-related low back pain, busy race schedules and recovery, nutrition, airbag technology, mouthguards, safety equipment and venue setup.

**Supplementary Information:**

The online version contains supplementary material available at 10.1186/s40798-025-00818-9.

## Background

Competitive snow sports are performance-driven and carry high injury risks [1–5], which can impact athletes ‘performance [6, 7]. The International Ski and Snowboard Federation (FIS) has established various prevention efforts to mitigate these risks [8–10]. Despite research and prevention initiatives over the last decade across all stages of prevention, snow sports, and competition levels [2, 3, 11–21], injury rates and severity remain high [1, 22–28], highlighting the need for more effective preventive actions. Current research faces challenges, including small sample sizes, methodological issues, the constant evolution of injury factors and their interaction with equipment, and competitive rules and regulations, particularly in competitive alpine skiing [15, 29, 30]. Additionally, there is limited research on other snow sports disciplines [3, 21, 24, 25, 27, 31–33].

Understanding the perspectives of athletes and other stakeholders, such as coaches, medical and technical staff, industry suppliers and FIS representatives [5], is essential for developing effective, context-driven prevention strategies [5, 11, 34–38]. While some studies have explored stakeholders’ views on injury prevention [5, 11], knowledge remains scarce, particularly at levels below the WC. These lower levels differ in resources, experience, and needs, making tailored approaches critical. [5, 11, 15, 16, 35, 36]

Gathering insights into stakeholders’ needs, perceived responsibilities, and preferred communication methods for prevention could inform future strategies [39]. Moreover, the nature and characteristics of the different stakeholder groups need to be acknowledged [5, 34, 40, 41]. In this respect, cross-sectional surveys have proven helpful in identifying and understanding stakeholders’ views on health and safety practices [11, 42–44]. Further actions need to consider the incidence but, most importantly, the severity and burden of injuries and illnesses. Ultimately, these preventive actions would foster more role-specific, snow sport-specific, and competition level-specific approaches. [39, 45]

This study surveyed stakeholders’ current practices, beliefs, needs, responsibilities and knowledge dissemination preferences regarding injury and illness prevention in competitive snow sports. Furthermore, we described the main commonalities and differences between FIS snow sport disciplines, stakeholder roles and competition levels.

## Methods

### Study Design and Setting

We conducted a cross-sectional survey study with stakeholders in the competitive snow sports context under the umbrella of FIS. The Cantonal Ethics Committee KEK Zurich (BASEC Nr. Req. 2021-01329) reviewed the present study protocol and judged it not to fall within the scope of the Swiss Human Research Act (HRA), which is why no informed consent was required from the participants.

### Population

We targeted all potential stakeholders in competitive snow sports who could be involved in injury and illness prevention. These consisted of athletes, coaches, team staff (e.g., health professionals and technical team members), ski racing supplier (SRS) representatives, and FIS representatives from the FIS competitive snow sports setting and their competition levels, such as alpine skiing, freestyle skiing, snowboarding, cross-country skiing, ski jumping, Nordic combined, and all the corresponding subdisciplines. From this point forward, these stakeholders from competitive snow sports under the umbrella of FIS are referred to as respondents.

### Survey Development and Design

The survey was developed in English by the research team, which consisted of five experts and researchers from the sports medicine field, all from different nations and areas of expertise. OBM is a PhD candidate from Andorra and a sports physiotherapist with experience in alpine skiing injury prevention. JS is a Swiss human movement scientist with extensive experience in alpine skiing research and injury prevention. VG is a French sports medical scientist, researcher and former professional athlete. CB is a Brazilian sports physical therapist with experience in sports injuries and a postdoctoral researcher. EV is a Dutch sports scientist and epidemiologist with thorough expertise in injury prevention.

We used Kipling’s principle, the “5W1H” method, to elaborate the survey. It is a systematic problem-solving procedure that uses 5 "W" (what, where, when, why, who) and 1 "H" (how) questions to view ideas from various perspectives and gain an in-depth understanding of a specific situation [46]. The survey intended to capture demographic data and respond to the main questions “*what*”, “*where*”, “*when/how often*”, “*why*”, “*who*”, and *“how”* regarding their current practices, beliefs, needs, responsibilities and knowledge dissemination preferences related to injury and illness prevention [47]. Therefore, it consisted of three parts: (1) demographic information and expertise (8 questions); (2) current practices, beliefs and responsibilities related to injury and illness prevention (9 questions); and (3) needs and communication preferences related to injury and illness prevention knowledge and its dissemination (9 questions). We incorporated closed-ended questions (e.g., a list of choices or yes and no answers) to obtain straightforward answers and open-ended questions to empower respondents to provide more detailed explanations. The detailed survey is available in Supplementary File 1. We pilot-tested the survey to determine the survey aim, content, readability and completion time with four external experts and researchers from the competitive snow sport context (e.g., physiotherapists, coaches, sports psychologists, sports scientists and former athletes across different snow sports). Minor changes were made to improve its clarity and completion.

### Data Collection

The survey was distributed between September and December 2023 via email by FIS to the registered email addresses of the Alpine Sports Directors of all National Ski and Snowboard Associations (NSSAs), FIS and SRS representatives, who were subsequently asked to distribute the survey among their competitive snow sports communities. The survey was closed two months after delivery (December 1st). Participation was voluntary and anonymous. The data were collected and administered through the REDCap electronic data capture tool [48, 49]. The data were assessed and stored in encrypted form and did not include any health-related data. The study used the Consensus-Based Checklist for Reporting of Survey Studies (CROSS), which is presented in Supplementary File 2. [50]

### Data Analysis

The data were cleaned and prepared for further analysis by one author (OBM) to recognise missing data and correct any errors and spelling mistakes. Five responses could not be considered due to missing or incomplete responses (e.g., demographics). Next, we conducted a descriptive analysis of the survey. Continuous variables are presented as the means with standard deviations (± SDs), and categorical variables are presented as the frequencies with percentages and the medians with interquartile ranges (IQRs). We calculated the descriptive statistics using IBM SPSS, V.21 (IBM Corp, Armonk, New York, USA). We defined different ranking lists, including (1) for the overall group of participants, (2) for the six FIS snow sport disciplines, (3) for the five stakeholder roles, and (4) for the different competition levels.

For the open-ended questions, we employed thematic analysis following a semantic approach to collect, analyse and summarise data from the open-ended questions (“*what*”, “*why*”, “*where*”, and “*when*”), following the six phases of the thematic analysis presented by Braun and Clark [51]. First, two independent researchers (OBM and CB) familiarised themselves with the data, generated initial codes, searched for themes, reviewed themes, defined and named them, and produced a report. A consensus was reached between OBM and CB. A concept map was developed to reduce the amount of data and analyse the interconnections between categories.

## Results

### Participants

**Fig. 1:**
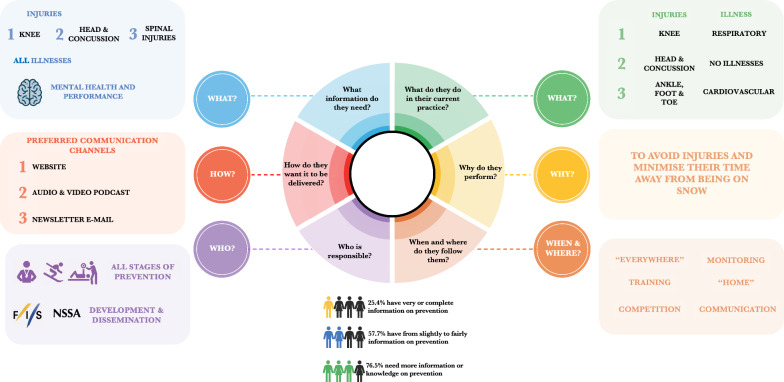
Diagram outlining the results for the “*what*”, “*why*”, “*when*”, “*where*”, “*who*” and “*how*” questions

A total of 441 snow sports stakeholders accessed the online questionnaire, five of whom were excluded because their questionnaire was incomplete. Among the 436 respondents, 79.4% completed the full survey (n = 346). They represented 23 nations from 5 continents (Supplementary File 3), mainly from France (21.3%, n = 92), Italy (15.5%, n = 67), the United States (13.0%, n = 56) and Great Britain (11.1%, n = 48). Many respondents held various roles and participated in different snow sports disciplines, subdisciplines, and competitions. The characteristics of the respondents are shown in Table 1, and a detailed description can be found in Supplementary File 4. The team staff roles included physiotherapists (n = 22), team directors (n = 10), strength & conditioning (S&C) coaches (n = 7), ski technicians (n = 6), manager or head coaches (n = 5), medical doctors (n = 4), team officials (n = 2), communication staff (n = 2), team coordinators (n = 2), psychologists (n = 1), nutritionists (n = 1) and team chefs (n = 1). The FIS representatives included roles such as technical delegates, officials, committee members, secretaries, and athletes' representatives. Figure 1 depicts all the information obtained through the present study. Supplementary Files 5–13 provide detailed information on the findings stratified by snow sports, stakeholder role, and competition level.

**Table 1:** Participant characteristics

		Stakeholder role				
	Total	Athlete	Coach	Team Staff	SRS—Industry	FIS representatives
**Respondents**	**463 (100.0)**	261 (56.4)	116 (25.1)	68 (14.7)	5 (1.1)	13 (2.8)
**Gender**						
Female	**168 (38.5)**	129 (49.4)	19 (16.4)	21 (30.9)	0 (0.0)	4 (30.8)
Male	**264 (60.6)**	129 (49.4)	97 (83.6)	47 (69.1)	5 (100.0)	8 (61.5)
Non-binary	**3 (0.7)**	2 (0.8)	0 (0.0)	0 (0.0)	0 (0.0)	1 (7.7)
I prefer not to say	**1 (0.2)**	1 (0.4)	0 (0.0)	0 (0.0)	0 (0.0)	0 (0.0)
**Age (median [Q3-Q1])**	**3 (5–2)**	2 (3–2)	5 (6–4)	5 (6–4)	5 (6–5)	6 (6.5–4)
**Years of experience in snow sports (mean [SD])**	**20.1 (13.0)**	14.6 (8.1)	31.1 (14.0)	22.7 (14.3)	33.0 (8.4)	32.5 (12.3)
**Snow sports**						
Alpine Skiing	**205 (44.0)**	100 (48.8)	62 (30.2)	30 (14.6)	5 (2.4)	8 (3.9)
Freestyle Skiing	**93 (20.0)**	41 (44.1)	25 (26.9)	20 (21.5)	3 (3.2)	4 (4.3)
Snowboarding	**72 (15.5)**	27 (37.5)	18 (25.0)	21 (29.0)	0 (0.0)	6 (8.3)
Cross-Country Skiing	**85 (18.2)**	50 (58.8)	13 (15.3)	18 (21.2)	1 (1.2)	3 (3.5)
Ski Jumping	**52 (11.2)**	36 (69.2)	6 (11.5)	9 (17.3)	0 (0.0)	1 (1.9)
Nordic Combined	**31 (6.7)**	10 (32.3)	10 (32.3)	11 (35.5)	0 (0.0)	0 (0.0)
**Competition levels**						
Youth competitions	**205 (20.0)**	88 (42.9)	74 (36.1)	33 (16.1)	3 (1.5)	7 (3.4)
FIS competitions	**327 (31.9)**	188 (57.2)	79 (24.2)	44 (13.5)	4 (1.2)	12 (3.7)
Continent Cups	**197 (19.2)**	94 (47.7)	62 (31.5)	29 (14.7)	5 (2.5)	7 (3.6)
World Cup	**198 (19.3)**	90 (45.5)	49 (24.7)	48 (24.2)	5 (2.5)	6 (3.0)
Master competitions	**26 (2.5)**	10 (38.5)	10 (38.5)	5 (19.2)	0 (0.0)	1 (3.8)
Mass events	**20 (1.9)**	6 (30.0)	5 (25.0)	7 (35.0)	0 (0.0)	2 (10.0)
Snow sport not governed by FIS	**53 (5.2)**	16 (30.2)	19 (35.8)	16 (30.2)	1 (1.9)	1 (1.9)

Values are presented in absolute numbers (and percentages in brackets) if not specified otherwise

Participants could answer the same question multiple times according to their characteristics

Age categories: (1) 10–14 y; (2) 15–19 y; (3) 20–29 y; (4) 30–39 y; (5) 40–49 y; (6) 50–59 y; (7) 60–69 yo; (8) 70–79 y; and (9) 80–89 y

### Current Prevention Practices

#### Which Injuries and Illnesses Receive Attention

The primary targets of prevention reported by body location were the knee (82.8% of respondents), the head and concussions (52.5%), and the ankle, foot and toe (41.3%). The knee and head injuries and concussions were considerably consistent across all subgroups, whereas the targeted third body location differed across all levels (Table 2).

**Table 2:** Stakeholders’ reported targets within their current injury prevention practices, with stratified subgroup ranks

Injury frequent targets	%	Overall rank	Snow sports						Stakeholder roles				
			Alpine Skiing	Freestyle Skiing	Snowboarding	Cross-Country Skiing	Ski Jumping	Nordic Combined	Athlete	Coach	Team Staff	SRS—Industry	FIS representatives
			*Subgroups rank*										
Head Injuries & Concussions	**52.5**	**2**	2	2	1	6	5	4	2	2	2	1	2
Shoulder, Upper Arm & Elbow Injuries	**37.4**	**4**	6	3	3	1	7	3	4	4	6	6	3
Forearm, Wrist & Hand Injuries	**19.5**	**8**	8	8	8	8	10	10	8	8	9	14	3
Thoracic Injuries	**9.2**	**11**	11	10	10	11	11	9	11	9	10	14	10
Abdominal Injuries	**6.2**	**12**	12	12	12	11	13	10	12	12	13	0	10
Spinal Injuries	**37.2**	**5**	4	3	4	5	6	7	6	3	3	3	3
Pelvic Injuries	**10.1**	**10**	10	9	9	10	9	10	10	11	8	–	8
Hip & Groin Injuries	**24.5**	**7**	7	7	6	6	3	5	7	7	7	6	9
Thigh Injuries	**12.6**	**9**	9	11	11	9	8	8	9	10	11	6	10
Knee Injuries	**82.8**	**1**	1	1	1	2	1	1	1	1	1	1	1
Lower Leg & Tibia/Shin Injuries	**34.9**	**6**	3	5	7	4	3	5	5	6	3	3	3
Ankle, Foot & Toe Injuries	**41.3**	**3**	5	6	4	2	2	2	3	5	5	5	3
Other injuries	**4.8**	**13**	13	–	13	11	13	13	13	13	12	14	10
None	**3.2**	**14**	14	12	14	14	11	13	13	14	14	14	10

Injury frequent targets	Competition levels						
	Youth competitions	FIS competitions	Continent Cups	World Cup	Master competitions	Mass events	Snow sport not governed by FIS
	*Subgroups rank*						
Head Injuries & Concussions	2	2	2	2	2	6	2
Shoulder, Upper Arm & Elbow Injuries	5	4	3	3	3	2	3
Forearm, Wrist & Hand Injuries	8	8	8	8	7	7	8
Thoracic Injuries	11	11	11	10	10	9	9
Abdominal Injuries	13	12	12	12	10	13	11
Spinal Injuries	4	5	4	4	5	3	6
Pelvic Injuries	10	10	10	10	9	9	10
Hip & Groin Injuries	7	7	7	7	8	3	7
Thigh Injuries	9	9	9	9	10	11	11
Knee Injuries	1	1	1	1	1	1	1
Lower Leg & Tibia/Shin Injuries	6	6	6	6	5	8	5
Ankle, Foot & Toe Injuries	3	3	5	5	3	5	4
Other injuries	12	13	13	13	13	11	13
None	14	14	14	14	14	13	14

FIS: International Ski and Snowboard Federation, SRS: Ski racing suppliers

Among the illnesses, respiratory (39.4%) and cardiovascular illnesses (22.5%) were their primary prevention targets, whereas 37.8% of respondents reported targeting no specific illness (Table 3). This pattern was also found across all subgroups, although there were differences among the first, second and third highest-ranked illnesses. At the open-ended questions, the respondents also added lacerations, mental health and gastrointestinal illnesses as prevention targets.

**Table 3:** Stakeholders’ reported targets within their current illness prevention practices, with stratified subgroup ranks

Illness frequent targets	%	Overall rank	Snow sports						Stakeholder roles				
			Alpine Skiing	Freestyle Skiing	Snowboarding	Cross-Country Skiing	Ski Jumping	Nordic Combined	Athlete	Coach	Team Staff	SRS—Industry	FIS representatives
			*Subgroups rank*										
Cardiovascular illnesses	**22.5**	**3**	3	3	3	2	6	4	3	3	3	1	2
Endocrinological illnesses	**10.8**	**6**	6	6	6	3	4	3	5	5	4	6	3
Respiratory illnesses	**39.4**	**1**	2	1	2	1	2	1	2	1	1	2	1
Thermoregulatory illnesses	**14.9**	**4**	4	5	4	5	3	6	4	5	6	2	6
Other illnesses	**12.4**	**5**	5	4	5	6	4	5	6	4	5	4	4
None	**37.8**	**2**	1	2	1	4	1	2	1	1	2	4	4

Illness frequent targets	Competition levels						
	Youth competitions	FIS competitions	Continent Cups	World Cup	Master competitions	Mass events	Snow sports not governed by FIS
	*Subgroups rank*						
Cardiovascular illnesses	3	3	3	3	3	2	4
Endocrinological illnesses	6	6	6	5	6	4	6
Respiratory illnesses	1	1	1	1	1	1	1
Thermoregulatory illnesses	4	4	5	4	5	4	3
Other illnesses	5	5	4	6	2	4	5
None	2	2	2	2	3	3	2

FIS: International Ski and Snowboard Federation, SRS: Ski racing suppliers

#### Targets of their Frequent Areas for Preventing Injury and Illness

The most common areas of prevention were physical aspects (88.5%), snow surfaces (75.2%), protective equipment and helmets (71.6%), mental aspects (70.4%) and visibility (70.2%) (Table 4). Physical aspects were mostly ranked highest across all subgroups. In contrast, the second and third-highest-ranked targets differed across disciplines, stakeholder roles and competition levels.

**Table 4:** Stakeholders’ reported prevention areas within their targets on their current injury and illness prevention practices, with stratified subgroup ranks

			Snow sports						Stakeholder roles				
Frequent targeted areas	%	Overall rank	Alpine Skiing	Freestyle Skiing	Snow-boarding	Cross-Country Skiing	Ski Jumping	Nordic Combined	Athlete	Coach	Team staff	SRS—Industry	FIS representatives
**A. Athlete-related areas**	**30.7**		29.5%	29.9%	31.0%	36.4%	31.2%	34.9%	29.3%	32.3%	34.5%	24.5%	26.1%
Physical aspects	**88.5**	**1**	1	1	1	1	4	1	1	1	1	1	3
Mental aspects	**70.4**	**4**	8	4	6	3	3	2	5	4	4	8	12
Skill/Technical/Tactical aspects	**64.9**	**6**	9	8	7	6	2	6	7	4	7	14	7
Load management	**45**	**14**	4	15	13	9	15	9	17	11	8	8	15
Education/Awareness	**50.9**	**11**	11	12	9	7	12	5	14	7	2	14	12
Others	**1.8**	**22**	20	20	22	22	20	22	22	21	21	14	22
**B. Equipment-related areas**	**27.1**		28.7%	26.6%	23.7%	22.5%	29.2%	25.4%	28.1%	26.0%	23.7%	38.8%	26.8%
Ski/board (incl. preparation)	**64.9**	**7**	5	7	10	8	11	10	6	8	9	1	7
Binding	**51.4**	**10**	10	11	18	17	5	6	12	10	10	1	7
Boot	**46.8**	**13**	13	13	14	14	10	14	13	15	13	5	7
Protectors/Helmet	**71.6**	**3**	4	3	2	11	7	10	2	9	11	5	4
Gear/Clothing	**44.7**	**15**	11	17	14	11	14	15	11	15	18	14	15
Others	**5**	**20**	21	22	20	19	19	20	20	21	19	–	–
**C. Course-related areas**	**19.6**		20.4%	21.7%	20.7%	17.3%	17.3%	17.4%	19.0%	20.3%	20.3%	20.4%	23.2%
Course design	**64.7**	**8**	5	6	4	2	16	16	8	3	5	7	7
Jump design	**42.7**	**16**	17	5	11	21	8	10	14	12	16	8	14
Safety nets	**49.8**	**12**	7	14	12	15	17	16	10	13	14	8	4
Jury decisions	**40.1**	**17**	15	16	16	16	8	6	16	17	14	8	4
Others	**8**	**19**	19	19	19	18	22	19	19	19	19	14	19
**D. Snow- and environment-related areas**	**22.7**		21.5%	21.8%	24.6%	23.8%	22.4%	22.3%	23.5%	21.5%	21.5%	16.3%	23.9%
Snow surface	**75.2**	**2**	2	9	5	5	6	4	3	2	3	1	1
Visibility	**70.2**	**5**	3	2	3	10	13	13	4	6	5	8	2
Temperature	**34.9**	**18**	18	18	17	3	18	16	18	18	17	22	15
Wind	**53.4**	**9**	16	9	8	13	1	3	9	13	11	14	15
Others	**4.1**	**21**	22	21	21	20	21	20	21	20	21	22	19

	Competition levels						
Frequent targeted areas	Youth competitions	FIS competitions	Continent Cups	World Cup	Master competitions	Mass events	Snow sports not governed by FIS
**A. Athlete-related areas**	30.9	31	31.3	30.7	27.5	31	32.2
Physical aspects	1	1	1	1	1	2	1
Mental aspects	4	3	3	3	8	1	2
Skill/Technical/Tactical aspects	5	7	6	7	6	5	9
Load management	13	14	14	12	18	17	10
Education/Awareness	9	9	10	14	10	5	7
Others	22	22	22	22	21	22	22
**B. Equipment-related areas**	27.3	26.9	26.1	26.1	29.1	24.1	24.6
Ski/board (incl. preparation)	7	6	8	9	8	10	13
Binding	10	11	13	10	4	14	10
Boot	12	15	15	16	10	13	13
Protectors/Helmet	3	5	4	5	6	9	4
Gear/Clothing	15	13	16	15	13	14	17
Others	20	20	21	20	21	19	20
**C. Course-related areas**	19.1	19.5	20.3	20.7	20.4	20.7	19.9
Course design	8	8	7	6	1	3	6
Jump design	16	16	11	11	15	14	12
Safety nets	13	12	12	13	10	17	15
Jury decisions	17	17	17	17	17	5	15
Others	19	19	19	19	19	19	19
**D. Snow- and environment-related areas**	22.7	22.7	22.4	22.6	23	24.1	23.4
TemperatSnow surfaceure	2	2	2	2	3	10	4
Visibility	5	4	5	4	4	3	3
Temperature	18	18	18	18	15	10	17
Wind	11	10	9	8	14	5	7
Others	21	21	20	21	19	19	21

FIS: International Ski and Snowboard Federation, SRS: Ski racing suppliers

### Their Beliefs Regarding Injury and Illness Prevention

Most of the surveyed stakeholders (87.5%) agreed on the importance of injury and illness prevention in snow sports, a distribution found across all the subgroups (Supplementary File 7). Furthermore, 46.4% of the respondents reported using injury and illness prevention strategies at least 5–6 days a week, whereas 38.0% spent between 1 and 4 days per week. The remaining respondents reported performing at most 2 days per month (10.4%) or not following any preventive measures (5.1%) (Supplementary File 7).

### Why they Follow Prevention Measures

The respondents described their major motivations to follow prevention measures referring to five underpinning reasons and tools, including (1) athletes’ health and welfare; (2) allowing athletes to perform, stressing their relationship with staying healthy; (3) avoiding injuries as a step to enjoy and have fun while doing the sport and ultimately “feeling good”; (4) safety and risk management; and (5) the coaching and team staff perspective, which stated that it is their job to follow injury and illness prevention measures.

### Where and when Prevention Occurs

The respondents reported performing prevention measures “*everywhere at any time*” in athletes’ daily lives. In this regard, four main occasions emerged: (1) before training and competition, which can occur in different scenarios; (2) during training, which involves physical and on-snow training; (3) during competitions, where communication plays a pivotal role; and (4) after training and competitions. Moreover, respondents reported that throughout the season, they carry out three additional blocks of prevention, involving (1) athletes’ health and performance monitoring, (2) education (seminars and workshops) and communication processes, and (3) at the medical centre and physiotherapy room in case the athlete needs treatment or further assistance.

### Needs and Future Opportunities

#### Do they Need more Information or Knowledge on Injury and Illness Prevention?

Approximately one quarter (25.4%) of the respondents reported having very or complete information, whereas 57.7% rated having slight to fair knowledge of prevention (Supplementary File 8). Similarly, three-quarters of the respondents (76.5%) reported needing more information or knowledge to strengthen their prevention strategies (Supplementary File 8).

#### Needs Regarding more Knowledge about Injuries and Illnesses

The main priorities by body location were knee injuries (64.4%), head injuries and concussions (57.3%), and spinal injuries (37.4%) (Table 5). This distribution was also present across all subgroups, with the knee and head (and concussions) ranking as the highest or second highest priority. In contrast, the third priority varied between subgroups.

**Table 5:** Stakeholders need more information or knowledge about injury prevention, with stratified subgroup ranks

Injury frequent targets	%	Overall rank	Snow sports	Stakeholder roles									
			Alpine Skiing	Freestyle Skiing	Snowboarding	Cross-Country Skiing	Ski Jumping	Nordic Combined	Athlete	Coach	Team Staff	SRS—Industry	FIS representatives
			*Subgroups rank*										
Head Injuries & Concussions	**57.3**	**2**	2	1	1	3	4	2	2	2	1	2	1
Shoulder, Upper Arm & Elbow Injuries	**26.4**	**6**	7	4	4	1	7	4	6	5	5	6	9
Forearm, Wrist & Hand Injuries	**13.8**	**9**	12	11	10	8	11	9	9	12	9	7	5
Thoracic Injuries	**13.3**	**11**	9	9	9	10	9	8	11	9	10	7	9
Abdominal Injuries	**13.8**	**9**	9	11	10	9	12	11	8	9	12	7	9
Spinal Injuries	**37.4**	**3**	3	3	2	6	5	5	3	3	3	3	3
Pelvic Injuries	**14.4**	**8**	8	8	7	10	8	11	12	8	8	7	5
Hip & Groin Injuries	**22**	**7**	6	7	6	7	6	7	7	7	7	7	5
Thigh Injuries	**13.3**	**11**	11	9	10	13	9	9	9	11	12	7	9
Knee Injuries	**64.4**	**1**	1	2	3	1	1	1	1	1	2	1	2
Lower Leg & Tibia/Shin Injuries	**29.6**	**5**	4	6	7	5	3	6	5	4	5	3	4
Ankle, Foot & Toe Injuries	**33**	**4**	5	4	5	4	2	3	4	6	4	3	5
Other injuries	**6**	**14**	13	14	14	14	14	14	14	13	14	7	14
None	**8.5**	**13**	14	13	13	10	13	13	13	14	10	7	9

Injury targeted needs	Competition levels						
	Youth competitions	FIS competitions	Continent Cups	World Cup	Master competitions	Mass events	Snow sport not governed by FIS
	*Subgroups rank*						
Head Injuries & Concussions	2	2	2	1	2	3	1
Shoulder, Upper Arm & Elbow Injuries	6	6	5	5	5	2	3
Forearm, Wrist & Hand Injuries	11	9	11	11	8	6	8
Thoracic Injuries	12	11	8	8	10	8	10
Abdominal Injuries	9	10	10	12	10	12	11
Spinal Injuries	3	3	3	3	4	4	5
Pelvic Injuries	8	8	8	9	6	9	7
Hip & Groin Injuries	7	7	7	7	8	10	8
Thigh Injuries	10	12	12	10	10	13	11
Knee Injuries	1	1	1	2	1	1	1
Lower Leg & Tibia/Shin Injuries	5	5	6	6	6	7	5
Ankle, Foot & Toe Injuries	4	4	4	4	3	5	4
Other injuries	13	13	13	14	10	14	11
None	14	13	14	13	10	11	14

FIS: International Ski and Snowboard Federation, SRS: Ski racing suppliers

For illnesses, the main priorities were respiratory (43.1%), cardiovascular (33.3%), endocrinological (31.7%), and thermoregulatory illnesses (30.7%), a distribution also found across all subgroups (Table 6). In the open-ended questions, respondents also reported on mental health training and lacerations as their additional needs for prevention.

**Table 6:** Stakeholders need more information or knowledge about illness prevention, with stratified subgroup ranks

Illness frequent targets	%	Overall rank	Snow sports	Stakeholder roles									
			Alpine Skiing	Freestyle Skiing	Snowboarding	Cross-Country Skiing	Ski Jumping	Nordic Combined	Athlete	Coach	Team Staff	SRS—Industry	FIS representatives
			*Subgroups rank*										
Cardiovascular illnesses	**33.3**	**2**	2	3	4	2	5	2	2	2	4	1	2
Endocrinological illnesses	**31.7**	**3**	3	4	3	3	1	4	4	3	2	3	2
Respiratory illnesses	**43.1**	**1**	1	1	1	1	3	1	1	1	1	1	1
Thermoregulatory illnesses	**30.7**	**4**	4	4	2	4	2	3	3	4	3	3	2
Other illnesses	**8.3**	**6**	6	6	6	6	6	5	6	6	6	3	6
None	**24.8**	**5**	5	2	4	5	4	6	5	4	5	3	2

Illness targeted needs	Competition levels						
	Youth competitions	FIS competitions	Continent Cups	World Cup	Master competitions	Mass events	Snow sports not governed by FIS
	*Subgroups rank*						
Cardiovascular illnesses	2	3	2	2	2	3	2
Endocrinological illnesses	3	2	3	3	4	4	4
Respiratory illnesses	1	1	1	1	1	1	1
Thermoregulatory illnesses	4	4	4	4	3	2	2
Other illnesses	6	6	6	6	5	6	6
None	5	5	5	5	5	5	5

FIS: International Ski and Snowboard Federation, SRS: Ski racing suppliers

#### Specific Needs Regarding Additional Knowledge About Athlete-, Equipment-, Course-, and Snow-Related Prevention Areas

The most prioritised areas included mental aspects (68.6%), physical aspects (64.2%), snow surface (59.2%), course design (53.0%) and ski and board (47.7%) (Table 7). Generally, regardless of the order, the same prevention areas were ranked across all subgroups.

**Table 7:** Stakeholders need more information or knowledge about injury and illness prevention, stratified by prevention area, with stratified subgroup ranks

Frequent targeted areas	%	Overall rank	Snow sports						Stakeholder roles				
			Alpine Skiing	Freestyle Skiing	Snow-boarding	Cross-Country Skiing	Ski Jumping	Nordic Combined	Athlete	Coach	Team Staff	SRS—Industry	FIS representatives
**A. Athlete-related areas**	**32.0**		31.4%	32.2%	32.7%	35.3%	31.6%	30.8%	31.9%	32.5%	32.8%	26.4%	32.8%
Physical aspects	**64.2**	**2**	3	2	2	1	7	1	2	2	2	1	1
Mental aspects	**68.6**	**1**	1	1	1	2	1	1	1	1	1	8	1
Skill/Technical/Tactical aspects	**40.4**	**8**	12	9	10	12	4	10	6	16	12	8	18
Load management	**36.2**	**12**	9	13	11	8	16	8	14	6	9	8	10
Education/Awareness	**33.9**	12	14	12	9	14	15	16	17	6	12	8	4
Social aspects	**26.1**	**19**	19	18	17	12	18	16	20	17	18	19	10
Others	**1.1**	**26**	26	26	25	24	23	23	26	27	25	17	25
None	**6.0**	**23**	23	23	23	23	23	26	23	25	25	19	23
**B. Equipment-related areas**	**24.9**		26.1%	22.5%	21.9%	20.4%	28.8%	27.1%	24.9%	24.4%	23.2%	37.7%	23.5%
Ski/board (incl. preparation)	**47.7**	**5**	6	11	12	6	6	6	5	8	7	1	4
Binding	**39.4**	**9**	8	16	15	21	2	1	11	5	7	1	10
Boot	**35.1**	**14**	13	17	21	16	8	9	13	11	11	6	8
Protectors/Helmet	**42.4**	**6**	5	6	5	20	13	13	7	9	16	6	15
Gear/Clothing	**27.8**	**18**	16	15	19	16	11	10	16	19	20	8	22
Others	**2.1**	**25**	24	24	24	24	23	26	24	23	23	19	23
None	**20.6**	**22**	22	22	21	10	21	21	21	21	21	19	18
**C. Course-related areas**	**20.6**		21.5%	22.8%	22.1%	19.1%	17.2%	19.4%	20.6%	21.5%	19.5%	18.9%	21.0%
Course design	**53.0**	**4**	4	3	2	5	13	10	4	4	4	1	4
Jump design	**35.3**	**13**	15	3	6	22	8	7	12	9	14	8	18
Safety nets	**28.9**	**17**	11	14	12	19	20	19	18	15	17	17	4
Jury decisions	**36.7**	**11**	10	9	15	8	10	13	10	11	15	8	10
Others	**0.9**	**27**	27	27	27	24	27	23	27	26	25	19	25
None	**22.9**	**20**	21	21	19	11	19	20	19	21	19	19	18
**D. Snow- and environment-related areas**	**22.5**		21.0%	22.5%	23.3%	25.2%	22.5%	22.7%	22.6%	21.6%	24.4%	17.0%	22.7%
Snow surface	**59.2**	**3**	1	5	4	3	5	5	3	2	2	1	3
Visibility	**41.1**	**7**	7	6	6	15	11	13	8	13	5	8	8
Temperature	**31.7**	**16**	17	20	12	4	16	16	15	17	9	8	10
Wind	**39.0**	**10**	18	8	8	7	2	1	9	14	6	19	15
Others	**2.3**	**24**	24	25	26	24	23	23	24	24	23	19	25
None	**20.9**	**21**	20	18	17	18	22	21	22	20	22	19	15

	Competition levels						
Needs targeted areas	Youth competitions	FIS competitions	Continent Cups	World Cup	Master competitions	Mass events	Snow sports not governed by FIS
**A. Athlete-related areas**	33.4%	33.1%	33.0%	31.5%	28.4%	32.1%	32.2%
Physical aspects	1	2	2	2	3	3	2
Mental aspects	2	1	1	1	3	1	1
Skill/Technical/Tactical aspects	6	6	6	12	10	17	6
Load management	10	14	13	9	17	10	8
Education/Awareness	12	13	9	13	10	17	12
Social aspects	17	17	17	19	17	5	19
Others	26	26	26	26	27	25	27
None	23	23	23	23	22	23	23
**B. Equipment-related areas**	24.8%	24.6%	24.8%	24.5%	25.2%	24.7%	22.7%
Ski/board (incl. preparation)	5	5	5	5	7	3	10
Binding	7	10	12	10	7	13	15
Boot	12	15	15	15	10	10	15
Protectors/Helmet	8	7	7	7	3	13	6
Gear/Clothing	18	19	18	18	19	13	18
Others	25	25	24	24	24	25	23
None	22	22	22	21	21	17	22
**C. Course-related areas**	19.9%	20.5%	20.4%	21.0%	22.5%	19.8%	20.8%
Course design	4	4	4	4	1	6	4
Jump design	16	12	9	11	15	10	11
Safety nets	19	18	19	16	10	17	19
Jury decisions	9	9	9	14	10	6	12
Others	27	27	27	26	24	25	26
None	20	20	20	20	22	21	15
**D. Snow- and environment-related areas**	22.0%	21.9%	21.9%	23.0%	23.9%	23.5%	24.3%
Snow surface	3	3	3	3	3	2	3
Visibility	12	8	8	7	2	9	8
Temperature	15	16	16	17	15	13	12
Wind	10	11	14	6	7	6	4
Others	24	24	24	24	24	23	25
None	21	21	20	21	20	22	21

FIS: International Ski and Snowboard Federation, SRS: Ski racing suppliers

Moreover, regarding athlete-related efforts, a need for increased knowledge on mental health and performance, female athletes, gradual onset-related low back pain, schedules, nutrition and recovery was reported. Regarding equipment-, course-, and snow-related aspects, the respondents highlighted interest in airbag technology and mouthguards, safety equipment, and venue setup. Finally, with respect to contextual elements, they reported that more efforts are required to educate athletes about safety and coaches about risk management, and they reported that they have easier access to information on the latest evidence and more availability of injury data.

#### Needs Regarding Additional Knowledge About Injury Registration, Warm-up/activation and Cool-down, Training and Testing, and Return to Sport

The respondents stated that their top priorities focused on the optimisation of (1) the return-to-sport process (RTS) (31.7%), (2) training methods (26.0%), (3) injury and illness registration methods (21.8%), (4) testing practices (19.8%), and (5) warm-up, activation and cool-down practices (18.7%) (Supplementary File 11).

One of the main issues raised within the open-ended questions was the need for more up-to-date research, including developing RTS guidelines and protocols for the most common snow sport-related injuries and more information on efficient training and training load management. Respondents likewise added training education for female-specific training and education regarding warm-ups and youth athletes, together with the development and implementation of routines regarding warm-ups (e.g., under cold conditions) and cool-down strategies. More data and benchmark values for testing and screening practices were also highlighted, along with a centralised and standardised reporting system optimally developed by FIS, allowing data comparison, tracking and monitoring.

### Responsibilities for Snow Sport-Related Injury and Illness Prevention Measures: Development, Dissemination and Implementation

**Fig. 2:**
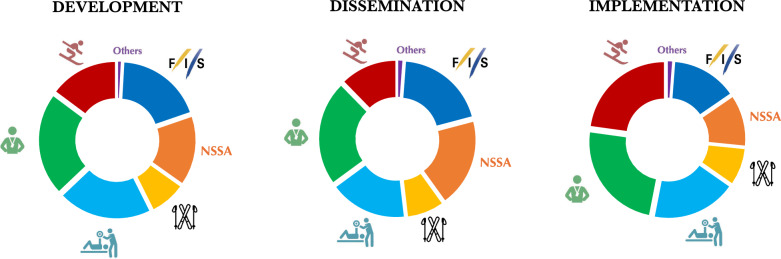
Stakeholders’ reported responsibilities across the development, dissemination and implementation of injury and illness prevention measures, with athletes (red), coaches (green), team staff (blue), SRS and industry (yellow), Regional and NSSAs (orange), FIS (blue) and other stakeholders (purple)

Coaches (62.6%), team staff (57.1%), and FIS (53.0%) were reported as the main accountable roles in developing prevention strategies. Regarding the dissemination of preventive measures, respondents referred to coaches (65.6%), FIS (56.9%), regional or NSSAs (55.3%) and team staff (48.4%). For implementation, the answers indicated coaches (81.7%), athletes (77.3%) and team staff (62.2%) (Fig. 2). Moreover, differences across subgroups were found, and they can be found in Supplementary File 12. Finally, all the respondents agreed that parents should play a role in all stages of prevention for young athletes.

### Knowledge Dissemination Preferences

The stakeholders’ preferred tools were diverse, as were the differences across subgroups, with a website (35.3%), audio and video podcasts (34.6% and 32.1%) and newsletters by e-mail (31.4%) as the most reported preferences (Table 8). Social media was also reported by 28.7% of the respondents, and Instagram was the most frequently mentioned platform (65%), followed by Facebook (11.9%), TikTok (8.4%) and YouTube (7.7%).

**Table 8:** Stakeholders’ preferred information channels for disseminating information or knowledge on injury and illness prevention, with stratified subgroup ranks

Communication channels	%	Overall rank	Snow sports	Stakeholder roles									
			Alpine Skiing	Freestyle Skiing	Snowboarding	Cross-Country Skiing	Ski Jumping	Nordic Combined	Athlete	Coach	Team Staff	SRS—Industry	FIS representatives
			*Subgroups rank*										
Book	**20.2**	**9**	9	10	9	9	10	7	9	8	6	4	8
e-Book	**30.3**	**5**	6	4	4	6	5	5	5	4	3	4	1
Magazine print	**13.1**	**14**	11	11	14	12	13	10	12	12	13	–	8
Magazine digital	**17.7**	**10**	10	9	11	11	7	10	8	9	10	4	2
Newsletter print	**8**	**12**	14	14	14	14	13	14	14	14	14	7	8
Newsletter e-mail	**31.4**	**4**	4	6	2	2	5	5	6	4	4	1	2
Podcasts audio	**34.6**	**2**	3	1	2	4	7	4	3	6	7	7	6
Podcasts video	**32.1**	**3**	2	2	5	7	9	8	4	3	8	1	7
Seminars	**27.3**	**8**	7	8	6	7	3	1	7	2	2	–	2
Webinars	**29.4**	**6**	5	5	1	4	2	1	10	1	1	7	5
Weblog	**4.4**	**15**	15	15	13	15	12	10	15	15	14	–	15
Website	**35.3**	**1**	1	7	6	1	1	1	2	7	4	1	8
Social media	**28.7**	**7**	8	3	8	3	4	10	1	11	11	–	8
Testimonials personal	**14.7**	**11**	13	13	10	9	11	9	11	10	9	–	13
Testimonials digital	**11.2**	**13**	11	1	12	12	13	15	13	12	12	7	13
Other/s	**2.3**	**16**	16	15	16	16	16	–	16	15	16	–	–

Communication channels	Competition levels						
	Youth competitions	FIS competitions	Continent Cups	World Cup	Master competitions	Mass events	Snow sport not governed by FIS
	*Subgroups rank*						
Book	9	9	9	8	11	7	7
e-Book	8	7	7	3	5	6	3
Magazine print	12	12	13	13	8	11	14
Magazine digital	10	10	10	11	9	7	12
Newsletter print	14	14	14	14	9	13	12
Newsletter e-mail	1	3	4	7	1	2	2
Podcasts audio	5	2	1	2	5	4	4
Podcasts video	4	4	2	6	4	7	6
Seminars	6	8	8	5	5	2	5
Webinars	2	6	5	1	3	1	1
Weblog	15	15	15	15	14	11	15
Website	2	1	3	4	1	4	7
Social media	6	5	6	9	14	7	11
Testimonials personal	11	11	11	10	12	13	9
Testimonials digital	13	13	12	12	12	13	10
Other/s	16	16	16	15	16	–	15

FIS: International Ski and Snowboard Federation, SRS: Ski racing suppliers

## Discussion

This study surveyed stakeholders in competitive snow sports to understand their practices, needs, responsibilities and knowledge dissemination preferences regarding injury and illness prevention. Stakeholders emphasised the importance of prevention and reported following strategies to minimise injury risks and reduce time away from training or competition. However, differences in prevention targets, priorities and approaches were evident across snow sports, roles, and competition levels, reflecting the diverse contexts and injury profiles of the field.

### A One-size Injury Prevention Approach Will not Fit All Snow Sports Alike

Prevention targets and priorities varied significantly among disciplines, influenced by each sport’s unique demands and injury patterns and the tasks performed within their teams. For example, spinal injuries were a specific focus in freestyle skiing and snowboarding, reflecting their perceived prevalence and consequences despite their limited representation in broader surveillance studies [2, 31]. These findings align with previous research highlighting the need for tailored, discipline-specific approaches [1, 5, 11, 24, 26, 27]. Context-specific interventions are vital for addressing the spectrum of challenges different snow sports face. With its established prevention frameworks, competitive alpine skiing could be a model for other snow sports to develop more nuanced and effective injury prevention measures.

### The Overlooked Need for Illness Prevention

Respiratory illness ranked among the top three practices and needs across all subgroups, yet 37.8% of the stakeholders reported that they did not target the prevention of any illness. The cold environments of snow sports, long outdoor exposure and competition structures such as on-snow training sessions and competition day structures (e.g., chair lift duration, waiting times, endurance discipline), present unique illness risks [35, 52–55]. While general illness prevention guidelines exist, stakeholders’ expressed needs suggest that current materials may not effectively reach or resonate with their intended audiences [56, 57]. Therefore, there is a need for more knowledge about illness prevention and for effective translation and dissemination of prevention knowledge, particularly in cold-weather contexts.

### Shared Responsibilities in Prevention

Stakeholders identified distinct roles across prevention stages: athletes and coaches were deemed central to all stages, while team staff, FIS and NSSAs were viewed as key to the development, dissemination and implementation of preventive measures. These findings resonate with socioecological sports injury prevention models, emphasising shared responsibilities across individual, sociocultural, and policy levels through active, passive, or both measures [34, 58–60]. In this regard, different levels have different tasks, levels of governance and responsibilities [61]. Athletes and their entourage (e.g., coaches and team staff) should engage in active prevention measures at the individual level. In contrast, governing bodies such as FIS and NSSAs must focus on structural and policy-level interventions to ensure a supportive environment for health and safety.

### Different Key Stakeholder Groups Want Different Communication Channels

Knowledge dissemination preferences varied by stakeholder group, likely reflecting differences in roles, generational factors, and the accessibility of communication channels [5, 11, 37, 38, 43, 62]. Thus, identifying their preferred dissemination preferences may ensure that information on prevention can reach the intended end-users. For instance, athletes and younger stakeholders may favour digital platforms such as social media, whereas coaches and team staff might prefer direct, role-specific resources. Prior research has shown that high-performance stakeholders rarely seek injury prevention knowledge from academic journals [38, 63], underscoring the importance of tailored, accessible communication strategies. [64, 65]

### Newly Emerging Topics in Athlete Health

Mental health and mental training emerged as a top priority, with stakeholders highlighting the need to better understand and integrate mental health and performance strategies into preventive practices [66]. Topics such as female athlete health, gradual onset low back pain in youth and elite athletes, and the demands of intense competition schedules also require greater attention. Indeed, raising awareness and spreading knowledge about female-specific challenges in competitive snow sports (e.g., the influence of the menstrual cycle and use of hormonal contraceptives on training, recovery and performance, and postpartum return to sport) may support female athletes and ensure an optimal environment for them to perform. These areas reflect evolving challenges in competitive snow sports and the importance of integrating mental, physical, and contextual factors into prevention efforts.

### Practical Recommendations

Findings from this study may inform governing bodies (FIS and NSSAs) in developing prevention approaches to the specific needs of each snow sport and competition level. Mental health and illness prevention should be prioritised, with strategies for effective knowledge dissemination via accessible platforms such as the FIS website, social media, and interactive resources. Collaborative workshops with experts and end-users help align science with practice, ensuring that preventive measures focusing on information dissemination, rules and regulations, and injury and illness prevention approaches are relevant and sustainable [41, 67]. Prevention should remain a collective effort [68, 69], with athletes and coaches at the centre of development and implementation, supported by governing bodies through active and passive measures.

### Strengths and Limitations

This study’s diverse sample, spanning multiple disciplines and competition levels, offers valuable insights, particularly for competition levels with less existing research, such as those beyond the WC level [1]. Moreover, we obtained a good representation of national teams from high- and low-resource settings with greater and lower snow sports culture [1, 5]. However, the uneven representation of roles and the survey design relying on closed-ended questions may have limited the breadth of perspectives captured. Moreover, because of the characteristics of networking and referrals, our sampling strategy facilitated reaching the whole competitive snow sports community, but two major pitfalls coexisted [70, 71]. Future research should address these gaps to provide a more comprehensive understanding of stakeholders’ perspectives.

## Conclusions

Competitive snow sports stakeholders highlighted diverse priorities and a desire for more knowledge on prevention within their contexts. These findings emphasise the need for tailored preventive approaches that address athlete, snow, equipment, course setting, and contextual factors, with shared responsibilities, cooperation and communication among stakeholders for prevention development, dissemination and implementation. Identifying preferred communication channels provides a pathway to effectively share knowledge. Hence, these findings draw a clear route toward each stakeholder group's specific routines, systems, and views to advance athletes' health.

## Supplementary Information

Supplementary Material 1

## Data Availability

All data relevant to the study are included in the manuscript or uploaded as supplemental information. Additional data are presented in the online Supplemental Files 1–13.

## Abbreviations

FIS: International Ski and Snowboard Federation
NSSA: National Ski and Snowboard Association
S&C: Strength and conditioning coach
SRS: Ski racing supplier
WC: World Cup
