# Supplementary material for: A Survey on Current Practices, Needs, Responsibilities and Preferences for Knowledge Dissemination in the Field of Injury and Illness Prevention Among Competitive Snow Sports Stakeholders
Source: Sports Med Open. 2025 Feb 19;11:17. doi: 10.1186/s40798-025-00818-9 (PMC11839548; doi:10.1186/s40798-025-00818-9)
Supplement: Supplementary file 1 — Supplementary Material 1 [file 40798_2025_818_MOESM1_ESM.pdf]

# A SURVEY ON CURRENT PRACTICES, NEEDS, RESPONSIBILITIES AND PREFERENCES FOR KNOWLEDGE DISSEMINATION IN THE FIELD OF INJURY AND ILLNESS PREVENTION AMONG COMPETITIVE SNOW SPORTS STAKEHOLDERS

**Journal: Sports Medicine Open**

**Authors**

Oriol Bonell Monsonís[a,b], Jörg Spörri[c,d], Vincent Gouttebarge[b,e,f], Caroline Bolling[a,e], Evert Verhagen[a,b]

a. Amsterdam Collaboration on Health & Safety in Sports, Department of Public and Occupational Health, Amsterdam Movement Sciences, Amsterdam UMC, University Medical Centres – Vrije Universiteit Amsterdam, Amsterdam, the Netherlands.
b. Amsterdam Movement Sciences, Musculoskeletal Health & Sports, Amsterdam, The Netherlands.
c. Sports Medical Research Group, Department of Orthopaedics, Balgrist University Hospital, University of Zurich, Zurich, Switzerland.
d. University Centre for Prevention and Sports Medicine, Department of Orthopaedics, Balgrist University Hospital, University of Zurich, Zurich, Switzerland.
e. Amsterdam UMC location University of Amsterdam, Department of Orthopaedic Surgery and Sports Medicine, Amsterdam, The Netherlands.
f. Section Sports Medicine, Faculty of Health Sciences, University of Pretoria, Pretoria, South Africa.

**Corresponding author**

Oriol Bonell Monsonís. E-mail address: o.bonellmonsonis@amsterdamumc.nl

**Supplementary files**

**Table of Contents**

**Supplementary file 1** ........ **4**

**FIS Needs Assessments Questionnaire** ........ 4

**Supplementary file 2** ........ **15**

**Consensus-Based Checklist for Reporting Of Survey Studies (CROSS)** ........ 15

**Supplementary file 3** ........ **20**

**Figure 1** Distribution of the 436 stakeholders surveyed ........ 20

**Supplementary file 4** ........ **21**

**Table 1** Participants characteristics. ........ 21

**Supplementary file 5** ........ **25**

**Table 1** Current practices on injuries. ........ 25

**Table 2** Current practices on illnesses. ........ 31

**Supplementary file 6** ........ **34**

**Table 1.A** Frequent targeted areas: overall, athlete- and equipment-related areas. ........ 34

**Table 1.B** Frequent targeted areas: course- and snow/environment-related areas. ........ 40

**Supplementary file 7** ........ **43**

**Table 1** Beliefs and frequency of prevention strategies. ........ 43

**Supplementary file 8** ........ **49**

**Table 1** Perspectives regarding having enough information or needing more information or knowledge on injury and illness prevention. ........ 49

**Supplementary file 9** ........ **52**

**Table 1** Needs for more information on injuries. ........................................................................52

**Table 2** Needs for more information on illnesses. ......................................................................58

**Supplementary file 10** .......................................................................................61

**Table 1.A** Needs for more information on targeted prevention areas: overall, athlete- and equipment-related areas. ..............................................................................................................61

**Table 1.B** Needs for more information on targeted areas: course- and snow/environment-related areas. ............................................................................................................................67

**Supplementary file 11** .......................................................................................72

**Table 1.A** Needs for more information on specific preventative intervention areas: injury and illness registration methods and warm-up, activation and cool-down strategies. .......................................72

**Table 1.B** Needs for more information on specific intervention areas: training methods and testing practices. ......................................................................................................................76

**Table 1.C** Needs for more information on specific intervention areas: the return-to-sport process. ..........................................................................................................................79

**Supplementary file 12** .......................................................................................82

**Table 1.A** Responsibilities across prevention stages: development and dissemination. ..................82

**Table 1.B** Responsibilities across prevention stages: implementation. ..........................................86

**Supplementary file 13** .......................................................................................89

**Table 1** Knowledge dissemination preferences. ...........................................................................89

**Supplementary file 1**

**FIS Needs Assessments Questionnaire**

# FIS Needs Assessments Questionnaire

Please complete the survey below.

Thank you!

---

FIS-AHU survey on the needs of snow sport stakeholders regarding the dissemination of knowledge on injury and illness prevention

The International Ski and Snowboard Federation's (FIS) Athlete Health Unit (AHU) has made it a priority to disseminate information on effective injury and illness prevention. In this regard, with this survey we aim to understand which information and methods are needed to help snow sports stakeholders to effectively protect the health of their athletes.

As a knowledgeable stakeholder in one of the FIS disciplines, we value your opinion. You are fully aware of the intricate problems related to injury and illness prevention in your discipline, and your knowledge helps us to chart the proper course for successful injury and illness prevention. This short survey is your chance to help formulating the requirements for possible contemporary FIS AHU knowledge dissemination/education initiatives.

The survey will take about 15 to 20 minutes to complete. There are no right or wrong answers to the questions. We are just interested in your knowledge, experience, and viewpoints.

Your response is collected anonymously, and only the project team members will have access to the survey data. Any resulting reports will only include summarized data.

We thank you for your participation on behalf of the project team and the FIS AHU.

## PART 1 - This first section of the questionnaire gathers context information on your background and expertise

| | |
|---|---|
| 1a) Which role do you primarily assign yourself? Multiple options can be answered. | ☐ Athlete<br>☐ Coach<br>☐ Team staff<br>☐ Equipment supplier representative / industry<br>☐ FIS representative |
| Please specify your role (e.g., medical doctor, physiotherapist, S&C coach, psychologist/mental coach, nutritionist, ski technician, etc.): | __________________________________ |
| Please specify your role (e.g., service man, engineer, R&D, management etc): | __________________________________ |
| Please specify your position (e.g., race director, committee member, etc): | __________________________________ |
| 1b) How do you identify yourself? | ☐ Female<br>☐ Male<br>☐ Non-binary<br>☐ I prefer not to say<br>☐ I prefer to self-describe |
| | __________________________________ |

| | |
|---|---|
| 1c) Which gender do your athletes identify with? | ☐ Female<br>☐ Male<br>☐ Both female and male<br>☐ Non-binary<br>☐ I prefer not to say<br>☐ I prefer to self-describe |
| | __________________________________ |
| 2) How many years have you been involved in this role?<br>[Type: Full numbers from 0-80] | __________________________________ |
| 3-i) In which FIS snow sport discipline(s) do you compete? Multiple options can be answered. | ☐ Alpine Skiing<br>☐ Freestyle Skiing<br>☐ Snowboarding<br>☐ Cross-Country Skiing<br>☐ Ski Jumping<br>☐ Nordic Combined |
| 3-ii) In which FIS snow sport discipline(s) do you work? Multiple options can be answered. | ☐ Alpine Skiing<br>☐ Freestyle Skiing<br>☐ Snowboarding<br>☐ Cross-Country Skiing<br>☐ Ski Jumping<br>☐ Nordic Combined |
| Please specify. Multiple options can be answered. | ☐ Slalom<br>☐ Giant Slalom<br>☐ Super-G<br>☐ Downhill<br>☐ Alpine Combined |
| Please specify. Multiple options can be answered. | ☐ Aerials<br>☐ Moguls<br>☐ Ski Cross<br>☐ Ski Halfpipe<br>☐ Ski Slopestyle |
| Please specify. Multiple options can be answered. | ☐ Snowboard Alpine<br>☐ Snowboard Cross<br>☐ Snowboard Halfpipe<br>☐ Snowboard Slopestyle<br>☐ Big Air |
| Please specify. Multiple options can be answered. | ☐ Sprint<br>☐ Distance |
| 4-i) In which competition format(s) of snow sport do you compete? Multiple options can be answered. | ☐ Youth competitions<br>☐ FIS competitions<br>☐ Continent Cups (e.g., European, North-American, South America, Asian, Australia/New Zealand)<br>☐ World Cup<br>☐ Master competitions<br>☐ Mass sport events<br>☐ Snow Sports events not governed by FIS (e.g. X-Games) |

| | |
|---|---|
| 4-ii) In which competition format(s) of snow sport do you work? Multiple options can be answered. | ☐ Youth competitions<br>☐ FIS competitions<br>☐ Continent Cups (e.g., European, North-American, South America, Asian, Australia/New Zealand)<br>☐ World Cup<br>☐ Master competitions<br>☐ Mass sport events<br>☐ Snow Sports events not governed by FIS (e.g. X-Games) |
| 5-i) At which training/performance level(s) of snow sport do you compete? Multiple options can be answered. | ☐ trained/developmental<br>☐ highly trained/National level<br>☐ elite/international level<br>☐ world class<br>☐ other |
| Please specify. | __________________________________ |
| 5-ii) At which training/performance level(s) of snow sport do you primarily work? Multiple options can be answered. | ☐ trained/developmental<br>☐ highly trained/National level<br>☐ elite/international level<br>☐ world class<br>☐ other |
| Please specify. | __________________________________ |
| 6) How many years have you been involved in snow sports in total? [Type: Full numbers from 1-70] | __________________________________ |
| 7) What is your age? | ◯ 10-14 years<br>◯ 15-19 years<br>◯ 20-29 years<br>◯ 30-39 years<br>◯ 40-49 years<br>◯ 50-59 years<br>◯ 60-69 years<br>◯ 70-79 years<br>◯ 80-89 years<br>◯ 90-99 years |
| 8-i) For which nation do you compete?<br><br>[e.g., "Andorra", "Netherlands", "Brasil", etc.] | __________________________________ |
| 8-ii) For which nation or organisation do you work?<br><br>[e.g., "Andorra", "Netherlands", "Brasil", etc.; if you work for an international institution (e.g. "IOC", "FIS"), a company (e.g. "equipment supplier") or as an "independent" expert, please state this with as specific a description as possible.] | __________________________________ |

**PART 2 - In this section, we are interested in your perceptions and perspectives on injury and illness prevention and your current practices.**

9a) Do you believe injury and illness prevention are important in snow sports?

◯ I don't know ◯ Not at all ◯ Slightly ◯ Somewhat ◯ Fairly ◯ Very ◯ Completely

9b) Could you briefly give reasoning for your answer?

10a) What kind of injuries are frequent targets of your prevention efforts? Multiple options can be answered.

- ☐ Head Injuries & Concussions
- ☐ Shoulder, Upper Arm & Elbow Injuries
- ☐ Forearm, Wrist & Hand Injuries
- ☐ Thoracic Injuries
- ☐ Abdominal Injuries
- ☐ Spinal Injuries
- ☐ Pelvic Injuries
- ☐ Hip & Groin Injuries
- ☐ Thigh Injuries
- ☐ Knee Injuries
- ☐ Lower Leg & Tibia/Shin Injuries
- ☐ Ankle, Foot & Toe Injuries
- ☐ Other injuries
- ☐ None

Can you briefly explain your answer?

__________________________________

10b) What kind of illnesses are frequent targets of your prevention efforts? Multiple options can be answered.

- ☐ Cardiovascular illnesses (i.e., health problems related to the heart or blood vessels)
- ☐ Endocrinological illnesses (i.e., health problems related to hormonal dysfunction)
- ☐ Respiratory illnesses (i.e., health problems affecting the respiratory system)
- ☐ Thermoregulatory illnesses (i.e., health problems in which abnormal changes in body temperature occur)
- ☐ Other illnesses
- ☐ None

Can you briefly explain your answer?

__________________________________

11a) What kind of athlete-related prevention areas are frequent targets of your prevention efforts? Multiple options can be answered.

- ☐ Physical Aspects
- ☐ Mental Aspects
- ☐ Skill/Technical/Tactical Aspects
- ☐ Load Management
- ☐ Education / Awareness
- ☐ Other/s

Can you briefly explain your answer?

__________________________________

| | |
|---|---|
| 11b) What kind of equipment-related prevention areas are frequent targets of your prevention efforts? Multiple options can be answered. | ☐ Ski / Board (including preparation)<br>☐ Binding<br>☐ Boot<br>☐ Protectors / Helmets<br>☐ Gear / Clothing<br>☐ Other/s |
| Can you briefly explain your answer? | ______________________________ |
| 11c) What kind of course-related prevention areas are frequent targets of your prevention efforts? Multiple options can be answered. | ☐ Course Design<br>☐ Jump Design<br>☐ Safety Nets<br>☐ Jury Decisions<br>☐ Other/s |
| Can you briefly explain your answer? | ______________________________ |
| 11d) What kind of snow/environment-related prevention areas are frequent targets of your prevention efforts? Multiple options can be answered. | ☐ Snow Surface<br>☐ Visibility<br>☐ Temperature<br>☐ Wind<br>☐ Other/s |
| Can you briefly explain your answer? | ______________________________ |
| 11e) What kind of other prevention areas are frequent targets of your prevention efforts? Please specify. If there is no other area, please write "none". | _____________________________________ |
| 12) How often do you use injury and illness prevention strategies? | ◯ >5-6 days/week (everyday)<br>◯ 5-6 days/week<br>◯ 3-4 days/week<br>◯ 1-2 days/week<br>◯ 1-2 days/month<br>◯ < 1 day/month<br>◯ Never |
| 13) Where do you perform snow sport-related injury and illness prevention strategies? | _____________________________________ |
| 14) Why do you follow your snow sport-related injury and illness prevention strategies or practices? | _____________________________________ |

15) How confident are you that your prevention efforts are effective?

◯ I don't know ◯ Not at all ◯ Slightly ◯ Somewhat ◯ Fairly ◯ Very ◯ Completely

| | |
|---|---|
| 16) In your opinion, who is responsible for applying/implementing snow sport-related injury and illness prevention measures? Multiple options can be answered. | ☐ Athlete<br>☐ Coach<br>☐ Team staff (medical or technical)<br>☐ Ski racing suplier representative / industry<br>☐ Regional/National Skiing and Snowboard Associations<br>☐ FIS<br>☐ Others |
| Please specify (e.g., medical doctor, physiotherapist, S&C coach, psychologist/mental coach, nutritionist, ski technician, etc.): | __________________________________ |
| Please specify (e.g., service man, research and development, etc): | __________________________________ |
| Please specify (e.g., staff for competitions, committee member, etc): | __________________________________ |
| Please specify | __________________________________ |
| 17) In your opinion, who is responsible for defining/developing snow sport-related injury and illness prevention measures? Multiple options can be answered. | ☐ Athlete<br>☐ Coach<br>☐ Team staff (medical or technical)<br>☐ Ski racing suplier representative / industry<br>☐ Regional/National Skiing and Snowboard Associations<br>☐ FIS<br>☐ Others |
| Please specify (e.g., medical doctor, physiotherapist, S&C coach, psychologist/mental coach, nutritionist, ski technician, etc.): | __________________________________ |
| Please specify (e.g., service man, research and development, etc): | __________________________________ |
| Please specify (e.g., staff for competitions, committee member, etc): | __________________________________ |
| Please specify | __________________________________ |
| 18) In your opinion, who is responsible for promoting/disseminating snow sport-related injury and illness prevention measures? Multiple options can be answered. | ☐ Athlete<br>☐ Coach<br>☐ Team staff (medical or technical)<br>☐ Ski racing suplier representative / industry<br>☐ Regional/National Skiing and Snowboard Associations<br>☐ FIS<br>☐ Others |
| Please specify (e.g., medical doctor, physiotherapist, S&C coach, psychologist/mental coach, nutritionist, ski technician, etc.): | __________________________________ |
| Please specify (e.g., service man, research and development, etc): | __________________________________ |

Please specify (e.g., staff for competitions, committee member, etc): __________________________________

Please specify __________________________________

## PART 3 - In this last section we ask about your needs, preferences, and expectations concerning the delivery of information on injury and illness prevention

19) Do you think you have enough information about snow sport-related injury and illness prevention?

○ I don't know ○ Not at all ○ Slightly ○ Somewhat ○ Fairly ○ Very ○ Completely

20) Do you feel like you need more information or knowledge in any of the above areas to strengthen your preventative efforts?

○ Yes
○ No

Could you briefly explain your answer (e.g., I would like to know more about the topic of ...)? __________________________________

21a) In order to better safeguard health in snow sports, what injuries and corresponding prevention measures/management strategies would you like to learn more about? Multiple options can be answered.

☐ Head Injuries & Concussions
☐ Shoulder, Upper Arm & Elbow Injuries
☐ Forearm, Wrist & Hand Injuries
☐ Thoracic Injuries
☐ Abdominal Injuries
☐ Spinal Injuries
☐ Pelvic Injuries
☐ Hip & Groin Injuries
☐ Thigh Injuries
☐ Knee Injuries
☐ Lower Leg & Tibia/Shin Injuries
☐ Ankle, Foot & Toe Injuries
☐ Other injuries
☐ None

Please specify __________________________________

21b) In order to better safeguard health in snow sports, what illnesses and corresponding prevention measures/management strategies would you like to learn more about? Multiple options can be answered.

☐ Cardiovascular illnesses (i.e., health problems related to the heart or blood vessels)
☐ Endocrinological illnesses (i.e., health problems related to hormonal dysfunction)
☐ Respiratory illnesses (i.e., health problems affecting the respiratory system)
☐ Thermoregulatory illnesses (i.e., health problems in which abnormal changes in body temperature occur)
☐ Other illnesses
☐ None

Please specify __________________________________

22) Which areas of prevention do you feel like you need more knowledge on to keep yourself or your athletes healthy? Multiple options can be answered.

22a) Athlete-related

☐ Physical Aspects
☐ Mental Aspects
☐ Skill / Technical / Tactical Aspects
☐ Load Management
☐ Education / Awareness
☐ Social Aspects
☐ Other/s
☐ None

Please specify

__________________________________

22b) Equipment-related

☐ Ski / Board (including preparation)
☐ Binding
☐ Boot
☐ Protectors / Helmets
☐ Gear / Clothing
☐ Other/s
☐ None

Please specify

__________________________________

22c) Course-related

☐ Course Design
☐ Jump Design
☐ Security Nets
☐ Jury Decisions
☐ Other/s
☐ None

Please specify

__________________________________

22d) Snow / environment-related

☐ Snow Surface
☐ Visibility
☐ Temperature
☐ Wind
☐ Other/s
☐ None

Please specify

__________________________________

22e) Others (please specify)

__________________________________________

23a) Do you feel like you need more information on how to optimize the recording of illness and injury cases?

◯ I don't know ◯ Not at all ◯ Slightly ◯ Somewhat ◯ Fairly ◯ Very ◯ Completely

23b) Could you briefly explain your answer?

__________________________________

24a) Do you feel like you need more information on how to optimize warm up, activate, and cool down?

◯ I don't know ◯ Not at all ◯ Slightly ◯ Somewhat ◯ Fairly ◯ Very ◯ Completely

24b) Could you briefly explain your answer?

__________________________________

25a) Do you feel like you need more information on how to optimize training?

◯ I don't know ◯ Not at all ◯ Slightly ◯ Somewhat ◯ Fairly ◯ Very ◯ Completely

25b) Could you briefly explain your answer?

__________________________________

26a) Do you feel like you need more information on how to optimize testing / screening?

◯ I don't know ◯ Not at all ◯ Slightly ◯ Somewhat ◯ Fairly ◯ Very ◯ Completely

26b) Could you briefly explain your answer?

__________________________________

27a) Do you feel like you need more information on how to optimize the return-to-sport process?

◯ I don't know ◯ Not at all ◯ Slightly ◯ Somewhat ◯ Fairly ◯ Very ◯ Completely

27b) Could you briefly explain your answer?

__________________________________

28) Through which way(s) would you prefer to obtain information on snow sport-related injury and illness prevention? Multiple options can be answered.

- ☐ Book (print)
- ☐ e-Book (digital)
- ☐ Magazine (print)
- ☐ e-Magazine (digital)
- ☐ Newsletter (print)
- ☐ Newsletter (e-mail)
- ☐ Podcasts (audio)
- ☐ Podcasts (video)
- ☐ Seminars (presence)
- ☐ Webinars (digital)
- ☐ Weblog
- ☐ Website
- ☐ Social media
- ☐ Delivery by testimonials (personal)
- ☐ Delivery by testimonials (digital)
- ☐ Other/s

Social media: Please specify the sources

__________________________________

Others: Please specify the sources

__________________________________

## PART 4- Additional personal comments

| | |
|---|---|
| 29) May we contact you in case we need further information? | ◯ Yes<br>◯ No |
| If yes, please provide your contact information | __________________________________ |
| 30) Do you want to add any additional comments? If yes, please specify here. | __________________________________________ |

**Supplementary file 2**

**Consensus-Based Checklist for Reporting Of Survey Studies (CROSS)**

Developed from: Sharma A, Minh Duc NT, Luu Lam Thang T, Nam NH, Ng SJ, Abbas KS, et al. A consensus-based checklist for reporting of survey studies (CROSS). *J Gen Intern Med*. 2021;36(10):3179–87.

| **Section/topic** | **Item** | **Item description** | **Reported on page #** |
|---|---|---|---|
| **Title and abstract** | | | |
| Title and abstract | 1a | State the word "survey" along with a commonly used term in title or abstract to introduce the study's design. | Title and abstract |
| | 1b | Provide an informative summary in the abstract, covering background, objectives, methods, findings/results, interpretation/discussion, and conclusions. | Abstract |
| **Introduction** | | | |
| Background | 2 | Provide a background about the rationale of study, what has been previously done, and why this survey is needed. | Background section |
| Purpose/aim | 3 | Identify specific purposes, aims, goals, or objectives of the study. | Background section |
| **Methods** | | | |
| Study design | 4 | Specify the study design in the methods section with a commonly used term (e.g., cross-sectional or longitudinal). | Study design and setting section |
| | 5a | Describe the questionnaire (e.g., number of sections, number of questions, number and names of instruments used). | Survey development and design section |

| | | | |
|---|---|---|---|
| Data collection methods | 5b | Describe all questionnaire instruments that were used in the survey to measure particular concepts. Report target population, reported validity and reliability information, scoring/classification procedure, and reference links (if any). | Target population, Data collection sections. |
| | 5c | Provide information on pretesting of the questionnaire, if performed (in the article or in an online supplement). Report the method of pretesting, number of times questionnaire was pre-tested, number and demographics of participants used for pretesting, and the level of similarity of demographics between pre-testing participants and sample population. | Survey development and design section |
| | 5d | Questionnaire if possible, should be fully provided (in the article, or as appendices or as an online supplement). | Supplementary file 1 |
| Sample characteristics | 6a | Describe the study population (i.e., background, locations, eligibility criteria for participant inclusion in survey, exclusion criteria). | Target population section, Table 1 |
| | 6b | Describe the sampling techniques used (e.g., single stage or multistage sampling, simple random sampling, stratified sampling, cluster sampling, convenience sampling). Specify the locations of sample participants whenever clustered sampling was applied. | Study design and setting section |
| | 6c | Provide information on sample size, along with details of sample size calculation. | Results section, Table 1 |
| | 6d | Describe how representative the sample is of the study population (or target population if possible), particularly for population-based surveys. | Target population section, Strengths and limitations |
| Survey administration | 7a | Provide information on modes of questionnaire administration, including the type and number of contacts, the location where the survey was conducted (e.g., outpatient room or by use of online tools, such as SurveyMonkey). | Data collection |
| | 7b | Provide information of survey's time frame, such as periods of recruitment, exposure, and follow-up days. | Data collection |

| | | | |
|---|---|---|---|
| | 7c | Provide information on the entry process:<br>–>For non-web-based surveys, provide approaches to minimize human error in data entry.<br>–>For web-based surveys, provide approaches to prevent "multiple participation" of participants. | Not reported/applicable |
| Study preparation | 8 | Describe any preparation process before conducting the survey (e.g., interviewers' training process, advertising the survey). | Not reported/applicable |
| Ethical considerations | 9a | Provide information on ethical approval for the survey if obtained, including informed consent, institutional review board [IRB] approval, Helsinki declaration, and good clinical practice [GCP] declaration (as appropriate). | Study design and setting section |
| | 9b | Provide information about survey anonymity and confidentiality and describe what mechanisms were used to protect unauthorized access. | Data collection |
| Statistical analysis | 10a | Describe statistical methods and analytical approach. Report the statistical software that was used for data analysis. | Data analysis |
| | 10b | Report any modification of variables used in the analysis, along with reference (if available). | Not reported/applicable |
| | 10c | Report details about how missing data was handled. Include rate of missing items, missing data mechanism (i.e., missing completely at random [MCAR], missing at random [MAR] or missing not at random [MNAR]) and methods used to deal with missing data (e.g., multiple imputation). | Data analysis |
| | 10d | State how non-response error was addressed. | Data analysis |
| | 10e | For longitudinal surveys, state how loss to follow-up was addressed. | Not reported/applicable |
| | 10f | Indicate whether any methods such as weighting of items or propensity scores have been used to adjust for non-representativeness of the sample. | Not reported/applicable |
| | 10g | Describe any sensitivity analysis conducted. | Not reported/applicable |

| | | | |
|---|---|---|---|
| **Results** | | | |
| Respondent characteristics | 11a | Report numbers of individuals at each stage of the study. Consider using a flow diagram, if possible. | Table 1 |
| | 11b | Provide reasons for non-participation at each stage, if possible. | Not reported/applicable |
| | 11c | Report response rate, present the definition of response rate or the formula used to calculate response rate. | Table 1 |
| | 11d | Provide information to define how unique visitors are determined. Report number of unique visitors along with relevant proportions (e.g., view proportion, participation proportion, completion proportion). | Results, Table 1 |
| Descriptive results | 12 | Provide characteristics of study participants, as well as information on potential confounders and assessed outcomes. | Results, Table 1 |
| Main findings | 13a | Give unadjusted estimates and, if applicable, confounder-adjusted estimates along with 95% confidence intervals and p-values. | Not reported/applicable |
| | 13b | For multivariable analysis, provide information on the model building process, model fit statistics, and model assumptions (as appropriate). | Not reported/applicable |
| | 13c | Provide details about any sensitivity analysis performed. If there are considerable amount of missing data, report sensitivity analyses comparing the results of complete cases with that of the imputed dataset (if possible). | Not reported/applicable |
| **Discussion** | | | |
| Limitations | 14 | Discuss the limitations of the study, considering sources of potential biases and imprecisions, such as non-representativeness of sample, study design, important uncontrolled confounders. | Strengths and limitations |
| Interpretations | 15 | Give a cautious overall interpretation of results, based on potential biases and imprecisions and | Practical recommendations, Key points |

| | | | |
|---|---|---|---|
| | | suggest areas for future research. | |
| Generalizability | 16 | Discuss the external validity of the results. | Strengths and limitations |
| **Other sections** | | | |
| Role of funding source | 17 | State whether any funding organization has had any roles in the survey's design, implementation, and analysis. | Declarations section |
| Conflict of interest | 18 | Declare any potential conflict of interest. | Declarations section |
| Acknowledgements | 19 | Provide names of organizations/persons that are acknowledged along with their contribution to the research. | Declarations section |

**Supplementary file 3**

**Figure 1** Distribution of the 436 stakeholders surveyed

A total of 23 countries were included, with an increase in colour density indicating an increased number of participants.

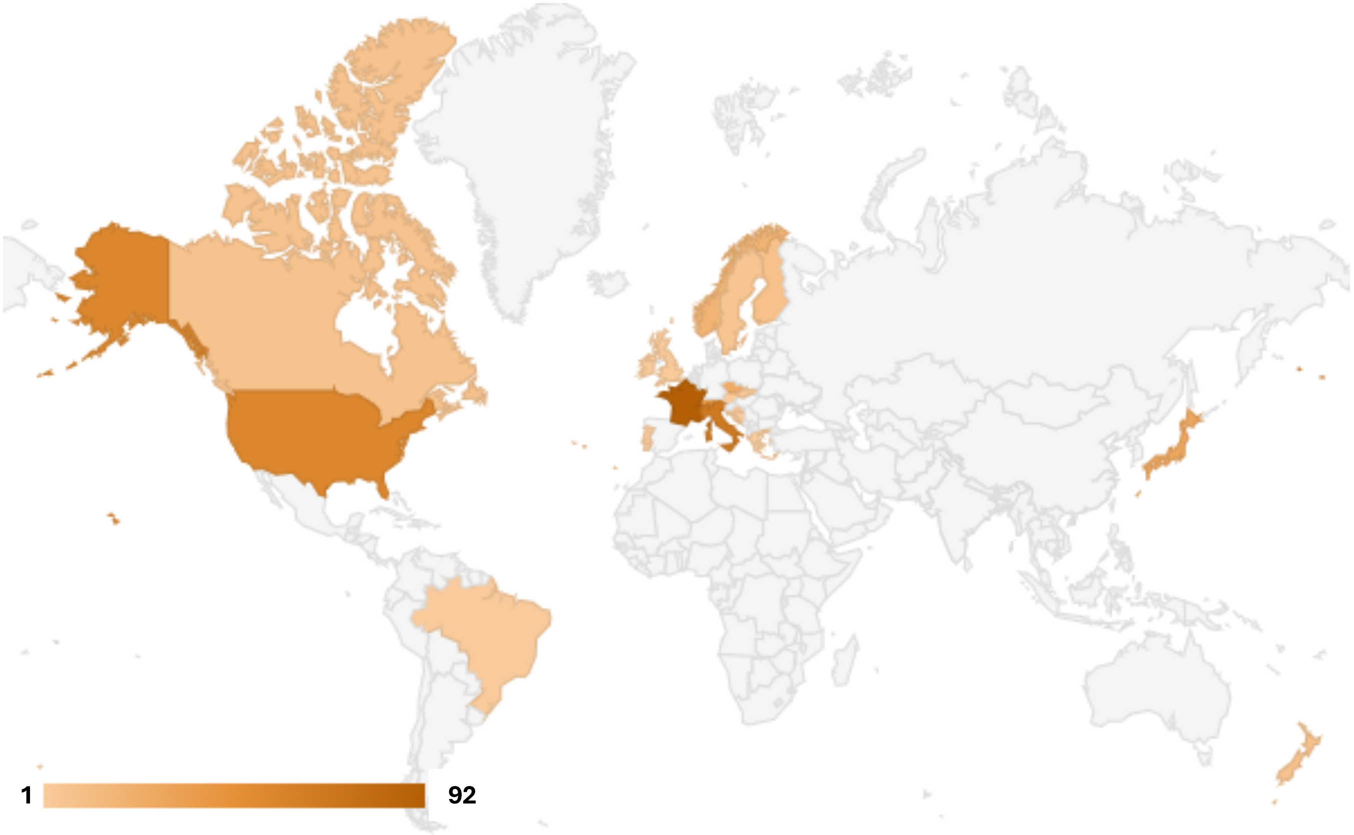

**Supplementary file 4**

**Table 1** Participants characteristics.

| **Stakeholder role** | **n (%)** | **Gender (n [%])** | | | | **Age (median [Q3-Q1])** | | | | | **Number of years of experience in snow sports (mean [SD])** |
|---|---|---|---|---|---|---|---|---|---|---|---|
| | | **Female** | **Male** | **Non-binary** | **I prefer not to say** | **Total** | **Female** | **Male** | **Non-binaryl** | **I prefer not to say** | **Total** |
| Athlete | 261 (56.4) | 129 (49.4) | 129 (49.4) | 2 (0.8) | 1 (0.4) | 2 (3-2) | 2 (3-2) | 2 (3-2) | 2.5 (2.5-2) | 4 (4-4) | 14.6 (8.1) |
| Coach | 116 (25.1) | 19 (16.4) | 97 (83.6) | 0 (0.0) | 0 (0.0) | 5 (6-4) | 5 (6-4) | 5 (6-4) | 0 (0) | 0 (0) | 31.1 (14.0) |
| Team staff | 68 (14.7) | 21 (30.9) | 47 (69.1) | 0 (0.0) | 0 (0.0) | 5 (6-4) | 5 (6-4) | 5 (6-4) | 0 (0) | 0 (0) | 22.7 (14.3) |
| Industry - SRS | 5 (1.1) | 0 (0.0) | 5 (100.0) | 0 (0.0) | 0 (0.0) | 5 (6-5) | 0 (0) | 5 (6-5) | 0 (0) | 0 (0) | 33.0 (8.4) |
| FIS representative | 13 (2.8) | 4 (30.8) | 8 (61.5) | 1 (7.7) | 0 (0.0) | 6 (6.5-4) | 5 (6-2.5) | 6 (7-5.25) | 4 (4-4) | 0 (0) | 32.5 (12.3) |
| **Total** | **463 (100.0)** | **168 (38.5)** | **264 (60.6)** | **3 (0.7)** | **1 (0.2)** | **3 (5-2)** | **3 (4-2)** | **4 (5-2)** | **3 (3.5-2.5)** | **4 (4-4)** | **20.1 (13.0)** |

Participants could answer the same question multiple times according to their characteristics.

Age categories: (1) 10-14 y; (2) 15-19 y; (3) 20-29 y; (4) 30-39 y; (5) 40-49 y; (6) 50-59 y; (7) 60-69 y; (8) 70-79 y; and (9) 80-89 y.

**Table 1** *continued.*

| Number of years of experience in snow sports (mean [SD]) | | | | Snow sport (n [%]) | | | | | | | | | |
|---|---|---|---|---|---|---|---|---|---|---|---|---|---|
| | | | | Alpine Skiing | | | | | | Freestyle Skiing | | | |
| Female | Male | Non-binary | I prefer not to say | Total | Slalom | Giant Slalom | Super-G | Downhill | Alpine Combined | Total | Aerials | Moguls | Ski Cross |
| 14.0 (5.4) | 15.1 (10.0) | 11.0 (4.2) | 30.0 (0.0) | 100 (48.8) | 91 (48.7) | 91 (47.9) | 80 (47.1) | 48 (39.0) | 41 (43.2) | 41 (44.1) | 3 (27.3) | 16 (45.7) | 7 (26.9) |
| 34.2 (14.0) | 30.5 (14.0) | 0.0 (0.0) | 0.0 (0.0) | 62 (30.2) | 56 (29.9) | 58 (30.5) | 53 (31.2) | 42 (34.1) | 29 (30.5) | 25 (26.9) | 3 (27.3) | 10 (28.6) | 6 (23.1) |
| 25.0 (15.4) | 21.6 (13.8) | 0.0 (0.0) | 0.0 (0.0) | 30 (14.6) | 28 (15.0) | 29 (15.3) | 24 (14.1) | 21 (17.1) | 15 (15.8) | 20 (21.5) | 5 (45.5) | 8 (22.9) | 7 (26.9) |
| 0.0 (0.0) | 33.0 (8.4) | 0.0 (0.0) | 0.0 (0.0) | 5 (2.4) | 5 (2.7) | 5 (2.6) | 5 (2.9) | 5 (4.1) | 5 (5.3) | 3 (3.2) | 0 (0.0) | 0 (0.0) | 3 (11.5) |
| 21.3 (7.5) | 38.5 (11.0) | 30.0 (0.0) | 0.0 (0.0) | 8 (3.9) | 7 (3.7) | 7 (3.7) | 8 (4.7) | 7 (5.7) | 5 (5.3) | 4 (4.3) | 0 (0.0) | 1 (2.9) | 3 (11.5) |
| **17.3 (10.5)** | **21.9 (14.1)** | **17.3 (11.4)** | **30.0 (0.0)** | **205 (44.0)** | **187 (24.4)** | **190 (24.8)** | **170 (22.2)** | **123 (16.1)** | **95 (12.4)** | **93 (20.0)** | **11 (8.2)** | **35 (26.1)** | **26 (19.4)** |

**Table 1** *continued.*

| **Competition level (n [%])** | | | | | | | | | | | | | **Competition level (n [%])** |
|---|---|---|---|---|---|---|---|---|---|---|---|---|---|
| **Freestyle Skiing** | | **Snowboarding** | | | | | | **Cross-Country Skiing** | | | **Ski Jumping** | **Nordic Combined** | **Youth competitions** |
| **Ski Halfpipe** | **Ski Slopestyle** | **Total** | **Alpine** | **Cross** | **Halfpipe** | **Slopestyle** | **Big Air** | **Total** | **Sprint** | **Distance** | | | |
| 2 (13.3) | 18 (38.3) | 27 (37.5) | 5 (21.7) | 13 (44.8) | 3 (16.7) | 8 (28.6) | 8 (26.7) | 50 (58.8) | 42 (57.5) | 48 (57.8) | 36 (69.2) | 10 (32.3) | 88 (42.9) |
| 6 (40.0) | 13 (27.7) | 18 (25.0) | 8 (34.8) | 6 (20.7) | 7 (38.9) | 9 (32.1) | 9 (30.0) | 13 (15.3) | 11 (15.1) | 13 (15.7) | 6 (11.5) | 10 (32.3) | 74 (36.1) |
| 7 (46.7) | 14 (29.8) | 21 (29.0) | 6 (26.1) | 7 (24.1) | 8 (44.4) | 10 (35.7) | 12 (40.0) | 18 (21.2) | 16 (21.9) | 18 (21.7) | 9 (17.3) | 11 (35.5) | 33 (16.1) |
| 0 (0.0) | 1 (2.1) | 0 (0.0) | 0 (0.0) | 0 (0.0) | 0 (0.0) | 0 (0.0) | 0 (0.0) | 1 (1.2) | 1 (1.4) | 1 (1.2) | 0 (0.0) | 0 (0.0) | 3 (1.5) |
| 0 (0.0) | 1 (2.1) | 6 (8.3) | 4 (17.4) | 3 (10.3) | 0 (0.0) | 1 (3.6) | 1 (3.3) | 3 (3.5) | 3 (4.1) | 3 (3.6) | 1 (1.9) | 0 (0.0) | 7 (3.4) |
| **15 (11.2)** | **47 (35.1)** | **72 (15.5)** | **23 (18.0)** | **29 (22.7)** | **18 (14.1)** | **28 (21.9)** | **30 (23.4)** | **85 (18.2)** | **73 (46.8)** | **83 (53.2)** | **11 (8.2)** | **35 (26.1)** | **205 (20.0)** |

**Table 1** *continued.*

| Competition level (n [%]) | | | | | | |
|---|---|---|---|---|---|---|
| **Youth competitions** | **FIS competitions** | **Continent Cups** | **World Cup** | **Master competitions** | **Mass events** | **Snow sports not governed by FIS** |
| 88 (42.9) | 188 (57.2) | 94 (47.7) | 90 (45.5) | 10 (38.5) | 6 (30.0) | 16 (30.2) |
| 74 (36.1) | 79 (24.2) | 62 (31.5) | 49 (24.7) | 10 (38.5) | 5 (25.0) | 19 (35.8) |
| 33 (16.1) | 44 (13.5) | 29 (14.7) | 48 (24.2) | 5 (19.2) | 7 (35.0) | 16 (30.2) |
| 3 (1.5) | 4 (1.2) | 5 (2.5) | 5 (2.5) | 0 (0.0) | 0 (0.0) | 1 (1.9) |
| 7 (3.4) | 12 (3.7) | 7 (3.6) | 6 (3.0) | 1 (3.8) | 2 (10.0) | 1 (1.9) |
| **205 (20.0)** | **327 (31.9)** | **197 (19.2)** | **198 (19.3)** | **26 (2.5)** | **20 (1.9)** | **53 (5.2)** |

**Supplementary file 5**

**Table 1** Current practices on injuries.

| | **Total** | | **Snow sports** | | | | | | | | | | | |
|---|---|---|---|---|---|---|---|---|---|---|---|---|---|---|
| | | | **Alpine Skiing (n=205)** | | **Freestyle Skiing (n=93)** | | **Snowboarding (n=72)** | | **Cross-Country Skiing (n=85)** | | **Ski Jumping (n=52)** | | **Nordic Combined (n=31)** | |
| | **n** | **%** | **n** | **%** | **n** | **%** | **n** | **%** | **n** | **%** | **n** | **%** | **n** | **%** |
| **Injury frequent targets** | | | | | | | | | | | | | | |
| Head Injuries & Concussions | **229** | **52.5%** | 124 | 60.5% | 61 | 65.6% | 52 | 72.2% | 19 | 22.4% | 13 | 25.0% | 9 | 29.0% |
| Shoulder, Upper Arm & Elbow Injuries | **163** | **37.4%** | 53 | 25.9% | 37 | 39.8% | 39 | 54.2% | 41 | 48.2% | 9 | 17.3% | 11 | 35.5% |
| Forearm, Wrist & Hand Injuries | **85** | **19.5%** | 39 | 19.0% | 11 | 11.8% | 19 | 26.4% | 17 | 20.0% | 4 | 7.7% | 3 | 9.7% |
| Thoracic Injuries | **40** | **9.2%** | 23 | 11.2% | 7 | 7.5% | 8 | 11.1% | 7 | 8.2% | 3 | 5.8% | 4 | 12.9% |
| Abdominal Injuries | **27** | **6.2%** | 15 | 7.3% | 1 | 1.1% | 5 | 6.9% | 7 | 8.2% | 2 | 3.8% | 3 | 9.7% |
| Spinal Injuries | **162** | **37.2%** | 84 | 41.0% | 37 | 39.8% | 31 | 43.1% | 23 | 27.1% | 10 | 19.2% | 7 | 22.6% |
| Pelvic Injuries | **44** | **10.1%** | 24 | 11.7% | 8 | 8.6% | 10 | 13.9% | 8 | 9.4% | 5 | 9.6% | 3 | 9.7% |
| Hip & Groin Injuries | **107** | **24.5%** | 46 | 22.4% | 19 | 20.4% | 22 | 30.6% | 19 | 22.4% | 15 | 28.8% | 8 | 25.8% |
| Thigh Injuries | **55** | **12.6%** | 27 | 13.2% | 6 | 6.5% | 7 | 9.7% | 10 | 11.8% | 7 | 13.5% | 5 | 16.1% |

| | | | | | | | | | | | | | | |
|---|---|---|---|---|---|---|---|---|---|---|---|---|---|---|
| Knee Injuries | **361** | **82.8%** | 175 | 85.4% | 76 | 81.7% | 52 | 72.2% | 40 | 47.1% | 47 | 90.4% | 26 | 83.9% |
| Lower Leg & Tibia/Shin Injuries | **152** | **34.9%** | 86 | 42.0% | 30 | 32.3% | 21 | 29.2% | 25 | 29.4% | 15 | 28.8% | 8 | 25.8% |
| Ankle, Foot & Toe Injuries | **180** | **41.3%** | 67 | 32.7% | 24 | 25.8% | 31 | 43.1% | 40 | 47.1% | 31 | 59.6% | 15 | 48.4% |
| Other injuries | **21** | **4.8%** | 9 | 4.4% | 0 | 0.0% | 2 | 2.8% | 7 | 8.2% | 2 | 3.8% | 1 | 3.2% |
| None | **14** | **3.2%** | 4 | 2.0% | 1 | 1.1% | 1 | 1.4% | 5 | 5.9% | 3 | 5.8% | 1 | 3.2% |

**Table 1** *continued.*

| | Stakeholder roles | | | | | | | | | | Competition levels | | | |
|---|---|---|---|---|---|---|---|---|---|---|---|---|---|---|
| | Athletes (n=261) | | Coaches (n=116) | | Team staff (n=68) | | Industry - SRS (n=5) | | FIS representatives (n=13) | | Youth competitions (n=205) | | FIS competitions (n=327) | |
| | n | % | n | % | n | % | n | % | n | % | n | % | n | % |
| **Injury frequent targets** | | | | | | | | | | | | | | |
| Head Injuries & Concussions | 121 | 46.4% | 74 | 63.8% | 36 | 52.9% | 5 | 100.0% | 7 | 53.8% | 95 | 46.3% | 159 | 48.6% |
| Shoulder, Upper Arm & Elbow Injuries | 98 | 37.5% | 47 | 40.5% | 25 | 36.8% | 1 | 20.0% | 4 | 30.8% | 66 | 32.2% | 121 | 37.0% |
| Forearm, Wrist & Hand Injuries | 54 | 20.7% | 21 | 18.1% | 11 | 16.2% | 0 | 0.0% | 4 | 30.8% | 35 | 17.1% | 62 | 19.0% |
| Thoracic Injuries | 19 | 7.3% | 16 | 13.8% | 10 | 14.7% | 0 | 0.0% | 1 | 7.7% | 15 | 7.3% | 27 | 8.3% |
| Abdominal Injuries | 15 | 5.7% | 11 | 9.5% | 5 | 7.4% | 0 | 0.0% | 1 | 7.7% | 12 | 5.9% | 18 | 5.5% |
| Spinal Injuries | 83 | 31.8% | 54 | 46.6% | 31 | 45.6% | 3 | 60.0% | 4 | 30.8% | 68 | 33.2% | 120 | 36.7% |
| Pelvic Injuries | 21 | 8.0% | 14 | 12.1% | 12 | 17.6% | 0 | 0.0% | 3 | 23.1% | 16 | 7.8% | 29 | 8.9% |
| Hip & Groin Injuries | 62 | 23.8% | 30 | 25.9% | 20 | 29.4% | 1 | 20.0% | 2 | 15.4% | 44 | 21.5% | 77 | 23.5% |
| Thigh Injuries | 34 | 13.0% | 15 | 12.9% | 8 | 11.8% | 1 | 20.0% | 1 | 7.7% | 30 | 14.6% | 35 | 10.7% |
| Knee Injuries | 208 | 79.7% | 107 | 92.2% | 55 | 80.9% | 5 | 100.0% | 8 | 61.5% | 153 | 74.6% | 252 | 77.1% |
| Lower Leg & Tibia/Shin Injuries | 85 | 32.6% | 41 | 35.3% | 31 | 45.6% | 3 | 60.0% | 4 | 30.8% | 65 | 31.7% | 105 | 32.1% |

| | | | | | | | | | | | | | | |
|---|---|---|---|---|---|---|---|---|---|---|---|---|---|---|
| Ankle, Foot & Toe Injuries | 111 | 42.5% | 46 | 39.7% | 28 | 41.2% | 2 | 40.0% | 4 | 30.8% | 83 | 40.5% | 128 | 39.1% |
| Other injuries | 10 | 3.8% | 5 | 4.3% | 6 | 8.8% | 0 | 0.0% | 1 | 7.7% | 14 | 6.8% | 17 | 5.2% |
| None | 10 | 3.8% | 1 | 0.9% | 2 | 2.9% | 0 | 0.0% | 1 | 7.7% | 5 | 2.4% | 8 | 2.4% |

**Table 1** *continued.*

| | **Competition levels** | | | | | | | | | |
|---|---|---|---|---|---|---|---|---|---|---|
| | **Continent Cups (n=197)** | | **World Cup (n=198)** | | **Master competitions (n=26)** | | **Mass events (n=20)** | | **Snow sports not governed by FIS (n=53)** | |
| | **n** | **%** | **n** | **%** | **n** | **%** | **n** | **%** | **n** | **%** |
| **Injury frequent targets** | | | | | | | | | | |
| Head Injuries & Concussions | 98 | 49.7% | 108 | 54.5% | 17 | 65.4% | 6 | 30.0% | 32 | 60.4% |
| Shoulder, Upper Arm & Elbow Injuries | 81 | 41.1% | 80 | 40.4% | 10 | 38.5% | 11 | 55.0% | 26 | 49.1% |
| Forearm, Wrist & Hand Injuries | 41 | 20.8% | 30 | 15.2% | 8 | 30.8% | 5 | 25.0% | 11 | 20.8% |
| Thoracic Injuries | 18 | 9.1% | 21 | 10.6% | 3 | 11.5% | 3 | 15.0% | 9 | 17.0% |
| Abdominal Injuries | 12 | 6.1% | 10 | 5.1% | 3 | 11.5% | 1 | 5.0% | 5 | 9.4% |
| Spinal Injuries | 77 | 39.1% | 78 | 39.4% | 9 | 34.6% | 8 | 40.0% | 17 | 32.1% |
| Pelvic Injuries | 20 | 10.2% | 21 | 10.6% | 5 | 19.2% | 3 | 15.0% | 7 | 13.2% |
| Hip & Groin Injuries | 47 | 23.9% | 51 | 25.8% | 7 | 26.9% | 8 | 40.0% | 14 | 26.4% |
| Thigh Injuries | 21 | 10.7% | 22 | 11.1% | 3 | 11.5% | 2 | 10.0% | 5 | 9.4% |
| Knee Injuries | 156 | 79.2% | 153 | 77.3% | 19 | 73.1% | 12 | 60.0% | 39 | 73.6% |
| Lower Leg & Tibia/Shin Injuries | 67 | 34.0% | 60 | 30.3% | 9 | 34.6% | 4 | 20.0% | 18 | 34.0% |

| | | | | | | | | | | |
|---|---|---|---|---|---|---|---|---|---|---|
| Ankle, Foot & Toe Injuries | 76 | 38.6% | 72 | 36.4% | 10 | 38.5% | 7 | 35.0% | 21 | 39.6% |
| Other injuries | 8 | 4.1% | 9 | 4.5% | 2 | 7.7% | 2 | 10.0% | 3 | 5.7% |
| None | 3 | 1.5% | 8 | 4.0% | 0 | 0.0% | 1 | 5.0% | 1 | 1.9% |

**Table 2** Current practices on illnesses.

| | Total | | Snow sports | | | | | | | | | | | |
|---|---|---|---|---|---|---|---|---|---|---|---|---|---|---|
| | | | **Alpine Skiing (n=205)** | | **Freestyle Skiing (n=93)** | | **Snowboarding (n=72)** | | **Cross-Country Skiing (n=85)** | | **Ski Jumping (n=52)** | | **Nordic Combined (n=31)** | |
| | **n** | **%** | **n** | **%** | **n** | **%** | **n** | **%** | **n** | **%** | **n** | **%** | **n** | **%** |
| **Illness frequent targets** | | | | | | | | | | | | | | |
| Cardiovascular illnesses | **98** | **22.5%** | 49 | 23.9% | 16 | 17.2% | 14 | 19.4% | 23 | 27.1% | 3 | 5.8% | 7 | 22.6% |
| Endocrinological illnesses | **47** | **10.8%** | 18 | 8.8% | 3 | 3.2% | 7 | 9.7% | 16 | 18.8% | 9 | 17.3% | 8 | 25.8% |
| Respiratory illnesses | **172** | **39.4%** | 67 | 32.7% | 33 | 35.5% | 23 | 31.9% | 51 | 60.0% | 14 | 26.9% | 17 | 54.8% |
| Thermoregulatory illnesses | **65** | **14.9%** | 25 | 12.2% | 9 | 9.7% | 10 | 13.9% | 11 | 12.9% | 13 | 25.0% | 3 | 9.7% |
| Other illnesses | **54** | **12.4%** | 23 | 11.2% | 14 | 15.1% | 9 | 12.5% | 9 | 10.6% | 9 | 17.3% | 6 | 19.4% |
| None | **165** | **37.8%** | 79 | 38.5% | 31 | 33.3% | 24 | 33.3% | 12 | 14.1% | 21 | 40.4% | 9 | 29.0% |

**Table 2** *continued.*

| | Stakeholder roles | | | | | | | | | | Competition levels | | | |
|---|---|---|---|---|---|---|---|---|---|---|---|---|---|---|
| | Athletes (n=261) | | Coaches (n=116) | | Team staff (n=68) | | Industry - SRS (n=5) | | FIS representatives (n=13) | | Youth competitions (n=205) | | FIS competitions (n=327) | |
| | n | % | n | % | n | % | n | % | n | % | n | % | n | % |
| **Illness frequent targets** | | | | | | | | | | | | | | |
| Cardiovascular illnesses | 63 | 24.1% | 23 | 19.8% | 13 | 19.1% | 3 | 60.0% | 5 | 38.5% | 35 | 17.1% | 69 | 21.1% |
| Endocrinological illnesses | 28 | 10.7% | 11 | 9.5% | 10 | 14.7% | 0 | 0.0% | 3 | 23.1% | 20 | 9.8% | 39 | 11.9% |
| Respiratory illnesses | 96 | 36.8% | 47 | 40.5% | 32 | 47.1% | 2 | 40.0% | 7 | 53.8% | 71 | 34.6% | 125 | 38.2% |
| Thermoregulatory illnesses | 45 | 17.2% | 11 | 9.5% | 8 | 11.8% | 2 | 40.0% | 1 | 7.7% | 30 | 14.6% | 47 | 14.4% |
| Other illnesses | 26 | 10.0% | 17 | 14.7% | 9 | 13.2% | 1 | 20.0% | 2 | 15.4% | 21 | 10.2% | 41 | 12.5% |
| None | 102 | 39.1% | 47 | 40.5% | 23 | 33.8% | 1 | 20.0% | 2 | 15.4% | 69 | 33.7% | 109 | 33.3% |

**Table 2** *continued.*

| | Competition levels | | | | | | | | | |
|---|---|---|---|---|---|---|---|---|---|---|
| | Continent Cups (n=197) | | World Cup (n=198) | | Master competitions (n=26) | | Mass events (n=20) | | Snow sports not governed by FIS (n=53) | |
| | n | % | n | % | n | % | n | % | n | % |
| **Illness frequent targets** | | | | | | | | | | |
| Cardiovascular illnesses | 44 | 22.3% | 49 | 24.7% | 6 | 23.1% | 6 | 30.0% | 9 | 17.0% |
| Endocrinological illnesses | 21 | 10.7% | 23 | 11.6% | 0 | 0.0% | 2 | 10.0% | 4 | 7.5% |
| Respiratory illnesses | 73 | 37.1% | 85 | 42.9% | 10 | 38.5% | 10 | 50.0% | 23 | 43.4% |
| Thermoregulatory illnesses | 25 | 12.7% | 28 | 14.1% | 3 | 11.5% | 2 | 10.0% | 10 | 18.9% |
| Other illnesses | 27 | 13.7% | 21 | 10.6% | 7 | 26.9% | 2 | 10.0% | 7 | 13.2% |
| None | 66 | 33.5% | 66 | 33.3% | 6 | 23.1% | 3 | 15.0% | 11 | 20.8% |

**Supplementary file 6**

**Table 1.A** Frequent targeted areas: overall, athlete- and equipment-related areas.

| | **Total** | | **Snow sports** | | | | | | | | | | | |
|---|---|---|---|---|---|---|---|---|---|---|---|---|---|---|
| | | | **Alpine Skiing (n=205)** | | **Freestyle Skiing (n=93)** | | **Snowboarding (n=72)** | | **Cross-Country Skiing (n=85)** | | **Ski Jumping (n=52)** | | **Nordic Combined (n=31)** | |
| | **n** | **%** | **n** | **%** | **n** | **%** | **n** | **%** | **n** | **%** | **n** | **%** | **n** | **%** |
| **Frequent targeted areas** | | | | | | | | | | | | | | |
| **A. Athlete-related areas** | **1402** | **30.7%** | 655 | 29.5% | 276 | 29.9% | 211 | 31.0% | 225 | 36.4% | 157 | 31.2% | 114 | 34.9% |
| Physical aspects | **386** | **88.5%** | 177 | 86.3% | 76 | 81.7% | 60 | 83.3% | 65 | 76.5% | 37 | 71.2% | 28 | 90.3% |
| Mental aspects | **307** | **70.4%** | 133 | 64.9% | 64 | 68.8% | 44 | 61.1% | 48 | 56.5% | 38 | 73.1% | 26 | 83.9% |
| Skill/technical/tactical aspects | **283** | **64.9%** | 128 | 62.4% | 54 | 58.1% | 41 | 56.9% | 40 | 47.1% | 40 | 76.9% | 19 | 61.3% |
| Load management | **196** | **45.0%** | 101 | 49.3% | 36 | 38.7% | 28 | 38.9% | 31 | 36.5% | 19 | 36.5% | 18 | 58.1% |
| Education/awareness | **222** | **50.9%** | 109 | 53.2% | 43 | 46.2% | 37 | 51.4% | 39 | 45.9% | 22 | 42.3% | 21 | 67.7% |
| Others | **8** | **1.8%** | 7 | 3.4% | 3 | 3.2% | 1 | 1.4% | 2 | 2.4% | 1 | 1.9% | 2 | 6.5% |

| | | | | | | | | | | | | | | |
|---|---|---|---|---|---|---|---|---|---|---|---|---|---|---|
| **B. Equipment-related areas** | **1240** | **27.1%** | 636 | 28.7% | 245 | 26.6% | 161 | 23.7% | 139 | 22.5% | 147 | 29.2% | 83 | 25.4% |
| Ski/board (including preparation) | **283** | **64.9%** | 144 | 70.2% | 55 | 59.1% | 34 | 47.2% | 38 | 44.7% | 26 | 50.0% | 17 | 54.8% |
| Binding | **224** | **51.4%** | 119 | 58.0% | 49 | 52.7% | 22 | 30.6% | 13 | 15.3% | 36 | 69.2% | 19 | 61.3% |
| Boot | **204** | **46.8%** | 104 | 50.7% | 40 | 43.0% | 24 | 33.3% | 24 | 28.2% | 27 | 51.9% | 14 | 45.2% |
| Protectors/helmet | **312** | **71.6%** | 154 | 75.1% | 69 | 74.2% | 53 | 73.6% | 27 | 31.8% | 34 | 65.4% | 17 | 54.8% |
| Gear/clothing | **195** | **44.7%** | 109 | 53.2% | 31 | 33.3% | 24 | 33.3% | 27 | 31.8% | 20 | 38.5% | 13 | 41.9% |
| Others | **22** | **5.0%** | 6 | 2.9% | 1 | 1.1% | 4 | 5.6% | 10 | 11.8% | 4 | 7.7% | 3 | 9.7% |

**Table 1.A** *continued.*

| | Stakeholder roles | | | | | | | | | | Competition levels | | | |
|---|---|---|---|---|---|---|---|---|---|---|---|---|---|---|
| | Athletes (n=261) | | Coaches (n=116) | | Team staff (n=68) | | Industry - SRS (n=5) | | FIS representatives (n=13) | | Youth competitions (n=205) | | FIS competitions (n=327) | |
| | n | % | n | % | n | % | n | % | n | % | n | % | n | % |
| **Frequent targeted areas** | | | | | | | | | | | | | | |
| **A. Athlete-related areas** | 764 | 29.3% | 447 | 32.2% | 244 | 34.5% | 12 | 24.5% | 37 | 26.1% | 606 | 30.9% | 986 | 31.0% |
| Physical aspects | 223 | 85.4% | 108 | 93.1% | 63 | 92.6% | 5 | 100.0% | 10 | 76.9% | 160 | 78.0% | 264 | 80.7% |
| Mental aspects | 182 | 69.7% | 91 | 78.4% | 45 | 66.2% | 2 | 40.0% | 7 | 53.8% | 126 | 61.5% | 215 | 65.7% |
| Skill/technical/tactical aspects | 165 | 63.2% | 91 | 78.4% | 40 | 58.8% | 1 | 20.0% | 8 | 61.5% | 125 | 61.0% | 199 | 60.9% |
| Load management | 91 | 34.9% | 72 | 62.1% | 39 | 57.4% | 2 | 40.0% | 5 | 38.5% | 87 | 42.4% | 139 | 42.5% |
| Education/awareness | 100 | 38.3% | 84 | 72.4% | 53 | 77.9% | 1 | 20.0% | 7 | 53.8% | 105 | 51.2% | 163 | 49.8% |
| Others | 3 | 1.1% | 1 | 0.9% | 4 | 5.9% | 1 | 20.0% | 0 | 0.0% | 3 | 1.5% | 6 | 1.8% |

| | | | | | | | | | | | | | | |
|---|---|---|---|---|---|---|---|---|---|---|---|---|---|---|
| **B. Equipment-related areas** | 733 | 28.1% | 361 | 26.0% | 168 | 23.7% | 19 | 38.8% | 38 | 26.8% | 535 | 27.3% | 856 | 26.9% |
| Ski/board (including preparation) | 170 | 65.1% | 83 | 71.6% | 36 | 52.9% | 5 | 100.0% | 8 | 61.5% | 122 | 59.5% | 200 | 61.2% |
| Binding | 120 | 46.0% | 77 | 66.4% | 35 | 51.5% | 5 | 100.0% | 8 | 61.5% | 99 | 48.3% | 152 | 46.5% |
| Boot | 116 | 44.4% | 59 | 50.9% | 33 | 48.5% | 4 | 80.0% | 8 | 61.5% | 92 | 44.9% | 138 | 42.2% |
| Protectors/helmet | 195 | 74.7% | 82 | 70.7% | 34 | 50.0% | 4 | 80.0% | 9 | 69.2% | 130 | 63.4% | 210 | 64.2% |
| Gear/clothing | 121 | 46.4% | 59 | 50.9% | 20 | 29.4% | 1 | 20.0% | 5 | 38.5% | 82 | 40.0% | 142 | 43.4% |
| Others | 11 | 4.2% | 1 | 0.9% | 10 | 14.7% | 0 | 0.0% | 0 | 0.0% | 10 | 4.9% | 14 | 4.3% |

**Table 1.A** *continued.*

| | **Competition levels** | | | | | | | | | |
|---|---|---|---|---|---|---|---|---|---|---|
| | **Continent Cups (n=197)** | | **World Cup (n=198)** | | **Master competitions (n=26)** | | **Mass events (n=20)** | | **Snow sports not governed by FIS (n=53)** | |
| | **n** | **%** | **n** | **%** | **n** | **%** | **n** | **%** | **n** | **%** |
| **Frequent targeted areas** | | | | | | | | | | |
| **A. Athlete-related areas** | 631 | 31.3% | 617 | 30.7% | 73 | 27.5% | 54 | 31.0% | 157 | 32.2% |
| Physical aspects | 166 | 84.3% | 172 | 86.9% | 20 | 76.9% | 13 | 65.0% | 39 | 73.6% |
| Mental aspects | 139 | 70.6% | 137 | 69.2% | 15 | 57.7% | 14 | 70.0% | 34 | 64.2% |
| Skill/technical/tactical aspects | 131 | 66.5% | 114 | 57.6% | 17 | 65.4% | 11 | 55.0% | 28 | 52.8% |
| Load management | 91 | 46.2% | 97 | 49.0% | 6 | 23.1% | 5 | 25.0% | 24 | 45.3% |
| Education/awareness | 102 | 51.8% | 93 | 47.0% | 14 | 53.8% | 11 | 55.0% | 30 | 56.6% |
| Others | 2 | 1.0% | 4 | 2.0% | 1 | 3.8% | 0 | 0.0% | 2 | 3.8% |

| | | | | | | | | | | |
|---|---|---|---|---|---|---|---|---|---|---|
| **B. Equipment-related areas** | 526 | 26.1% | 524 | 26.1% | 77 | 29.1% | 42 | 24.1% | 120 | 24.6% |
| Ski/board (including preparation) | 121 | 61.4% | 111 | 56.1% | 15 | 57.7% | 9 | 45.0% | 22 | 41.5% |
| Binding | 95 | 48.2% | 102 | 51.5% | 18 | 69.2% | 7 | 35.0% | 24 | 45.3% |
| Boot | 85 | 43.1% | 83 | 41.9% | 14 | 53.8% | 8 | 40.0% | 22 | 41.5% |
| Protectors/helmet | 138 | 70.1% | 132 | 66.7% | 17 | 65.4% | 10 | 50.0% | 32 | 60.4% |
| Gear/clothing | 84 | 42.6% | 84 | 42.4% | 12 | 46.2% | 7 | 35.0% | 16 | 30.2% |
| Others | 3 | 1.5% | 12 | 6.1% | 1 | 3.8% | 1 | 5.0% | 4 | 7.5% |

**Table 1.B** Frequent targeted areas: course- and snow/environment-related areas.

| | Total | | Snow sports | | | | | | | | | | | |
|---|---|---|---|---|---|---|---|---|---|---|---|---|---|---|
| | | | Alpine Skiing (n=205) | | Freestyle Skiing (n=93) | | Snowboarding (n=72) | | Cross-Country Skiing (n=85) | | Ski Jumping (n=52) | | Nordic Combined (n=31) | |
| | n | % | n | % | n | % | n | % | n | % | n | % | n | % |
| **C. Course-related areas** | **895** | **19.6%** | 452 | 20.4% | 200 | 21.7% | 141 | 20.7% | 107 | 17.3% | 87 | 17.3% | 57 | 17.4% |
| Course design | **282** | **64.7%** | 144 | 70.2% | 57 | 61.3% | 50 | 69.4% | 52 | 61.2% | 13 | 25.0% | 8 | 25.8% |
| Jump design | **186** | **42.7%** | 68 | 33.2% | 61 | 65.6% | 33 | 45.8% | 3 | 3.5% | 31 | 59.6% | 17 | 54.8% |
| Safety nets | **217** | **49.8%** | 140 | 68.3% | 39 | 41.9% | 30 | 41.7% | 21 | 24.7% | 12 | 23.1% | 8 | 25.8% |
| Jury decisions | **175** | **40.1%** | 88 | 42.9% | 34 | 36.6% | 23 | 31.9% | 19 | 22.4% | 31 | 59.6% | 19 | 61.3% |
| Others | **35** | **8.0%** | 12 | 5.9% | 9 | 9.7% | 5 | 6.9% | 12 | 14.1% | 0 | 0.0% | 5 | 16.1% |
| **D. Snow- and environment-related areas** | **1037** | **22.7%** | 476 | 21.5% | 201 | 21.8% | 167 | 24.6% | 147 | 23.8% | 113 | 22.4% | 73 | 22.3% |
| Snow surface | **328** | **75.2%** | 172 | 83.9% | 53 | 57.0% | 48 | 66.7% | 41 | 48.2% | 35 | 67.3% | 22 | 71.0% |
| Visibility | **306** | **70.2%** | 158 | 77.1% | 71 | 76.3% | 52 | 72.2% | 28 | 32.9% | 21 | 40.4% | 15 | 48.4% |
| Temperature | **152** | **34.9%** | 61 | 29.8% | 22 | 23.7% | 23 | 31.9% | 48 | 56.5% | 11 | 21.2% | 8 | 25.8% |
| Wind | **233** | **53.4%** | 79 | 38.5% | 53 | 57.0% | 40 | 55.6% | 25 | 29.4% | 45 | 86.5% | 25 | 80.6% |
| Others | **18** | **4.1%** | 6 | 2.9% | 2 | 2.2% | 4 | 5.6% | 5 | 5.9% | 1 | 1.9% | 3 | 9.7% |

**Table 1.B** *continued.*

| | Stakeholder roles | | | | | | | | | | Competition levels | | | |
|---|---|---|---|---|---|---|---|---|---|---|---|---|---|---|
| | Athletes (n=261) | | Coaches (n=116) | | Team staff (n=68) | | Industry - SRS (n=5) | | FIS representatives (n=13) | | Youth competitions (n=205) | | FIS competitions (n=327) | |
| | n | % | n | % | n | % | n | % | n | % | n | % | n | % |
| **C. Course-related areas** | 496 | 19.0% | 282 | 20.3% | 144 | 20.3% | 10 | 20.4% | 33 | 23.2% | 373 | 19.1% | 619 | 19.5% |
| Course design | 157 | 60.2% | 94 | 81.0% | 44 | 64.7% | 3 | 60.0% | 8 | 61.5% | 120 | 58.5% | 195 | 59.6% |
| Jump design | 100 | 38.3% | 68 | 58.6% | 26 | 38.2% | 2 | 40.0% | 6 | 46.2% | 76 | 37.1% | 129 | 39.4% |
| Safety nets | 126 | 48.3% | 61 | 52.6% | 32 | 47.1% | 2 | 40.0% | 9 | 69.2% | 87 | 42.4% | 145 | 44.3% |
| Jury decisions | 95 | 36.4% | 52 | 44.8% | 32 | 47.1% | 2 | 40.0% | 9 | 69.2% | 75 | 36.6% | 125 | 38.2% |
| Others | 18 | 6.9% | 7 | 6.0% | 10 | 14.7% | 1 | 20.0% | 1 | 7.7% | 15 | 7.3% | 25 | 7.6% |
| **D. Snow- and environment-related areas** | 612 | 23.5% | 299 | 21.5% | 152 | 21.5% | 8 | 16.3% | 34 | 23.9% | 444 | 22.7% | 721 | 22.7% |
| Snow surface | 187 | 71.6% | 99 | 85.3% | 46 | 67.6% | 5 | 100.0% | 12 | 92.3% | 145 | 70.7% | 226 | 69.1% |
| Visibility | 184 | 70.5% | 85 | 73.3% | 44 | 64.7% | 2 | 40.0% | 11 | 84.6% | 125 | 61.0% | 212 | 64.8% |
| Temperature | 85 | 32.6% | 51 | 44.0% | 24 | 35.3% | 0 | 0.0% | 5 | 38.5% | 73 | 35.6% | 110 | 33.6% |
| Wind | 146 | 55.9% | 61 | 52.6% | 34 | 50.0% | 1 | 20.0% | 5 | 38.5% | 95 | 46.3% | 161 | 49.2% |
| Others | 10 | 3.8% | 3 | 2.6% | 4 | 5.9% | 0 | 0.0% | 1 | 7.7% | 6 | 2.9% | 12 | 3.7% |

**Table 1.B** *continued.*

| | **Competition levels** | | | | | | | | | |
|---|---|---|---|---|---|---|---|---|---|---|
| | **Continent Cups (n=197)** | | **World Cup (n=198)** | | **Master competitions (n=26)** | | **Mass events (n=20)** | | **Snow sports not governed by FIS (n=53)** | |
| | **n** | **%** | **n** | **%** | **n** | **%** | **n** | **%** | **n** | **%** |
| **C. Course-related areas** | 409 | 20.3% | 416 | 20.7% | 54 | 20.4% | 36 | 20.7% | 97 | 19.9% |
| Course design | 124 | 62.9% | 129 | 65.2% | 20 | 76.9% | 12 | 60.0% | 31 | 58.5% |
| Jump design | 99 | 50.3% | 98 | 49.5% | 10 | 38.5% | 7 | 35.0% | 23 | 43.4% |
| Safety nets | 96 | 48.7% | 95 | 48.0% | 14 | 53.8% | 5 | 25.0% | 18 | 34.0% |
| Jury decisions | 82 | 41.6% | 78 | 39.4% | 7 | 26.9% | 11 | 55.0% | 18 | 34.0% |
| Others | 8 | 4.1% | 16 | 8.1% | 3 | 11.5% | 1 | 5.0% | 7 | 13.2% |
| **D. Snow- and environment-related areas** | 452 | 22.4% | 454 | 22.6% | 61 | 23.0% | 42 | 24.1% | 114 | 23.4% |
| Snow surface | 140 | 71.1% | 140 | 70.7% | 19 | 73.1% | 9 | 45.0% | 32 | 60.4% |
| Visibility | 136 | 69.0% | 134 | 67.7% | 18 | 69.2% | 12 | 60.0% | 33 | 62.3% |
| Temperature | 60 | 30.5% | 59 | 29.8% | 10 | 38.5% | 9 | 45.0% | 16 | 30.2% |
| Wind | 110 | 55.8% | 113 | 57.1% | 11 | 42.3% | 11 | 55.0% | 30 | 56.6% |
| Others | 6 | 3.0% | 8 | 4.0% | 3 | 11.5% | 1 | 5.0% | 3 | 5.7% |

## Supplementary file 7

**Table 1** Beliefs and frequency of prevention strategies.

| | Total | | Snow sports | | | | | | | | | | | |
|---|---|---|---|---|---|---|---|---|---|---|---|---|---|---|
| | | | Alpine Skiing (n=205) | | Freestyle Skiing (n=93) | | Snowboarding (n=72) | | Cross-Country Skiing (n=85) | | Ski Jumping (n=52) | | Nordic Combined (n=31) | |
| | n | % | n | % | n | % | n | % | n | % | n | % | n | % |
| **Beliefs in snow sports prevention** | | | | | | | | | | | | | | |
| I don't know | **11** | **2.5%** | 7 | 3.7% | 0 | 0.0% | 1 | 1.5% | 1 | 1.3% | 1 | 2.0% | 1 | 3.2% |
| Not at all | **7** | **1.6%** | 1 | 0.5% | 1 | 1.2% | 3 | 4.6% | 1 | 1.3% | 1 | 2.0% | 0 | 0.0% |
| Slightly | **6** | **1.4%** | 0 | 0.0% | 2 | 2.4% | 0 | 0.0% | 1 | 1.3% | 3 | 5.9% | 1 | 3.2% |
| Somewhat | **10** | **2.3%** | 7 | 3.7% | 0 | 0.0% | 0 | 0.0% | 3 | 3.9% | 0 | 0.0% | 1 | 3.2% |
| Fairly | **21** | **4.8%** | 9 | 4.8% | 4 | 4.9% | 3 | 4.6% | 3 | 3.9% | 1 | 2.0% | 2 | 6.5% |
| Very | **108** | **24.8%** | 40 | 21.3% | 24 | 29.3% | 9 | 13.8% | 21 | 27.3% | 15 | 29.4% | 9 | 29.0% |
| Completely | **272** | **62.5%** | 124 | 66.0% | 51 | 62.2% | 49 | 75.4% | 47 | 61.0% | 30 | 58.8% | 17 | 54.8% |

**Table 1** *continued.*

| Frequency of performing prevention | | | | | | | | | | | | | | |
|---|---|---|---|---|---|---|---|---|---|---|---|---|---|---|
| >5-6 days/week | **156** | **36.2%** | 81 | 43.5% | 24 | 29.6% | 23 | 36.5% | 21 | 27.3% | 7 | 13.7% | 12 | 38.7% |
| 5-6 days/week | **44** | **10.2%** | 21 | 11.3% | 13 | 16.0% | 6 | 9.5% | 4 | 5.2% | 4 | 7.8% | 1 | 3.2% |
| 3-4 days/week | **85** | **19.7%** | 38 | 20.4% | 14 | 17.3% | 18 | 28.6% | 14 | 18.2% | 16 | 31.4% | 4 | 12.9% |
| 1-2 days/week | **79** | **18.3%** | 27 | 14.5% | 19 | 23.5% | 8 | 12.7% | 21 | 27.3% | 9 | 17.6% | 8 | 25.8% |
| 1-2 days/month | **28** | **6.5%** | 12 | 6.5% | 4 | 4.9% | 3 | 4.8% | 6 | 7.8% | 2 | 3.9% | 3 | 9.7% |
| < 1 day/month | **17** | **3.9%** | 2 | 1.1% | 5 | 6.2% | 1 | 1.6% | 5 | 6.5% | 4 | 7.8% | 2 | 6.5% |
| Never | **22** | **5.1%** | 5 | 2.7% | 2 | 2.5% | 4 | 6.3% | 6 | 7.8% | 9 | 17.6% | 1 | 3.2% |

**Table 1** *continued.*

| | Stakeholder roles | | | | | | | | | | Competition levels | | | |
|---|---|---|---|---|---|---|---|---|---|---|---|---|---|---|
| | Athletes (n=261) | | Coaches (n=116) | | Team staff (n=68) | | Industry - SRS (n=5) | | FIS representatives (n=13) | | Youth competitions (n=205) | | FIS competitions (n=327) | |
| | n | % | n | % | n | % | n | % | n | % | n | % | n | % |
| **Beliefs in snow sports prevention** | | | | | | | | | | | | | | |
| I don't know | 11 | 4.2% | 0 | 0.0% | 0 | 0.0% | 0 | 0.0% | 0 | 0.0% | 3 | 1.6% | 9 | 3.0% |
| Not at all | 6 | 2.3% | 1 | 0.9% | 0 | 0.0% | 0 | 0.0% | 0 | 0.0% | 2 | 1.1% | 3 | 1.0% |
| Slightly | 5 | 1.9% | 0 | 0.0% | 1 | 1.5% | 0 | 0.0% | 0 | 0.0% | 1 | 0.5% | 3 | 1.0% |
| Somewhat | 5 | 1.9% | 4 | 3.4% | 1 | 1.5% | 0 | 0.0% | 0 | 0.0% | 4 | 2.2% | 7 | 2.3% |
| Fairly | 15 | 5.8% | 2 | 1.7% | 3 | 4.4% | 1 | 20.0% | 0 | 0.0% | 8 | 4.3% | 13 | 4.3% |
| Very | 59 | 22.7% | 33 | 28.4% | 14 | 20.6% | 3 | 60.0% | 5 | 38.5% | 50 | 27.2% | 73 | 23.9% |
| Completely | 159 | 61.2% | 76 | 65.5% | 49 | 72.1% | 1 | 20.0% | 8 | 61.5% | 116 | 63.0% | 197 | 64.6% |

**Table 1** *continued.*

| Frequency of performing prevention | | | | | | | | | | | | | | |
|---|---|---|---|---|---|---|---|---|---|---|---|---|---|---|
| >5-6 days/week | 83 | 32.0% | 57 | 49.6% | 25 | 37.9% | 1 | 20.0% | 3 | 25.0% | 57 | 31.1% | 106 | 35.2% |
| 5-6 days/week | 28 | 10.8% | 10 | 8.7% | 6 | 9.1% | 0 | 0.0% | 1 | 8.3% | 21 | 11.5% | 32 | 10.6% |
| 3-4 days/week | 52 | 20.1% | 22 | 19.1% | 13 | 19.7% | 1 | 20.0% | 3 | 25.0% | 38 | 20.8% | 63 | 20.9% |
| 1-2 days/week | 52 | 20.1% | 15 | 13.0% | 13 | 19.7% | 1 | 20.0% | 2 | 16.7% | 34 | 18.6% | 52 | 17.3% |
| 1-2 days/month | 17 | 6.6% | 6 | 5.2% | 3 | 4.5% | 0 | 0.0% | 3 | 25.0% | 14 | 7.7% | 20 | 6.6% |
| < 1 day/month | 10 | 3.9% | 5 | 4.3% | 2 | 3.0% | 1 | 20.0% | 0 | 0.0% | 10 | 5.5% | 12 | 4.0% |
| Never | 17 | 6.6% | 0 | 0.0% | 4 | 6.1% | 1 | 20.0% | 0 | 0.0% | 9 | 4.9% | 16 | 5.3% |

**Table 1** *continued.*

| | **Competition levels** | | | | | | | | | |
|---|---|---|---|---|---|---|---|---|---|---|
| | **Continent Cups (n=197)** | | **World Cup (n=198)** | | **Master competitions (n=26)** | | **Mass events (n=20)** | | **Snow sports not governed by FIS (n=53)** | |
| | **n** | **%** | **n** | **%** | **n** | **%** | **n** | **%** | **n** | **%** |
| **Beliefs in snow sports prevention** | | | | | | | | | | |
| I don't know | 1 | 0.5% | 0 | 0.0% | 0 | 0.0% | 0 | 0.0% | 0 | 0.0% |
| Not at all | 3 | 1.6% | 5 | 2.7% | 0 | 0.0% | 0 | 0.0% | 0 | 0.0% |
| Slightly | 0 | 0.0% | 4 | 2.2% | 0 | 0.0% | 1 | 6.3% | 0 | 0.0% |
| Somewhat | 2 | 1.1% | 4 | 2.2% | 0 | 0.0% | 0 | 0.0% | 1 | 2.2% |
| Fairly | 7 | 3.8% | 12 | 6.5% | 1 | 4.5% | 1 | 6.3% | 3 | 6.7% |
| Very | 51 | 27.9% | 39 | 21.0% | 3 | 13.6% | 4 | 25.0% | 14 | 31.1% |
| Completely | 119 | 65.0% | 122 | 65.6% | 18 | 81.8% | 10 | 62.5% | 27 | 60.0% |

**Table 1** *continued.*

| | | | | | | | | | | |
|---|---|---|---|---|---|---|---|---|---|---|
| **Frequency of performing prevention** | | | | | | | | | | |
| >5-6 days/week | 63 | 34.6% | 74 | 40.0% | 12 | 54.5% | 4 | 25.0% | 14 | 31.1% |
| 5-6 days/week | 21 | 11.5% | 19 | 10.3% | 2 | 9.1% | 1 | 6.3% | 7 | 15.6% |
| 3-4 days/week | 41 | 22.5% | 36 | 19.5% | 3 | 13.6% | 4 | 25.0% | 12 | 26.7% |
| 1-2 days/week | 35 | 19.2% | 33 | 17.8% | 2 | 9.1% | 3 | 18.8% | 7 | 15.6% |
| 1-2 days/month | 8 | 4.4% | 11 | 5.9% | 2 | 9.1% | 1 | 6.3% | 1 | 2.2% |
| < 1 day/month | 6 | 3.3% | 7 | 3.8% | 0 | 0.0% | 2 | 12.5% | 3 | 6.7% |
| Never | 8 | 4.4% | 5 | 2.7% | 1 | 4.5% | 1 | 6.3% | 1 | 2.2% |

**Supplementary file 8**

**Table 1** Perspectives regarding having enough information or needing more information or knowledge on injury and illness prevention.

| | **Total** | | **Snow sports** | | | | | | | | | | | |
|---|---|---|---|---|---|---|---|---|---|---|---|---|---|---|
| | | | **Alpine Skiing (n=205)** | | **Freestyle Skiing (n=93)** | | **Snowboarding (n=72)** | | **Cross-Country Skiing (n=85)** | | **Ski Jumping (n=52)** | | **Nordic Combined (n=31)** | |
| | **n** | **%** | **n** | **%** | **n** | **%** | **n** | **%** | **n** | **%** | **n** | **%** | **n** | **%** |
| **Having enough information** | | | | | | | | | | | | | | |
| I don't know | **19** | **4.6%** | 8 | 4.4% | 2 | 2.5% | 1 | 1.6% | 1 | 1.3% | 4 | 7.84% | 4 | 12.9% |
| Not at all | **51** | **12.4%** | 19 | 10.6% | 13 | 16.0% | 12 | 19.7% | 10 | 13.0% | 6 | 11.76% | 5 | 16.1% |
| Slightly | **54** | **13.1%** | 23 | 12.8% | 8 | 9.9% | 2 | 3.3% | 12 | 15.6% | 7 | 13.73% | 5 | 16.1% |
| Somewhat | **86** | **20.9%** | 41 | 22.8% | 16 | 19.8% | 15 | 24.6% | 15 | 19.5% | 14 | 27.45% | 6 | 19.4% |
| Fairly | **97** | **23.6%** | 49 | 27.2% | 19 | 23.5% | 11 | 18.0% | 15 | 19.5% | 10 | 19.61% | 4 | 12.9% |
| Very | **82** | **20.0%** | 32 | 17.8% | 18 | 22.2% | 15 | 24.6% | 21 | 27.3% | 8 | 15.69% | 6 | 19.4% |
| Completely | **22** | **5.4%** | 8 | 4.4% | 5 | 6.2% | 5 | 8.2% | 3 | 3.9% | 2 | 3.92% | 1 | 3.2% |
| **Feeling of needing more information or knowledge** | | | | | | | | | | | | | | |
| Yes | **323** | **76.5%** | 147 | 81.2% | 61 | 75.3% | 44 | 72.1% | 57 | 74.0% | 41 | 80.4% | 22 | 71.0% |
| No | **99** | **23.5%** | 34 | 18.8% | 20 | 24.7% | 17 | 27.9% | 20 | 26.0% | 10 | 19.6% | 9 | 29.0% |

**Table 1** *continued.*

| | Stakeholder roles | | | | | | | | | | Competition levels | | | |
|---|---|---|---|---|---|---|---|---|---|---|---|---|---|---|
| | Athletes (n=261) | | Coaches (n=116) | | Team staff (n=68) | | Industry - SRS (n=5) | | FIS representatives (n=13) | | Youth competitions (n=205) | | FIS competitions (n=327) | |
| | n | % | n | % | n | % | n | % | n | % | n | % | n | % |
| **Having enough information** | | | | | | | | | | | | | | |
| I don't know | 16 | 6.3% | 2 | 1.8% | 1 | 1.6% | 0 | 0.0% | 0 | 0.0% | 8 | 4.4% | 13 | 4.4% |
| Not at all | 28 | 10.9% | 14 | 12.7% | 10 | 16.1% | 0 | 0.0% | 3 | 23.1% | 27 | 14.9% | 34 | 11.5% |
| Slightly | 33 | 12.9% | 13 | 11.8% | 5 | 8.1% | 1 | 20.0% | 2 | 15.4% | 23 | 12.7% | 39 | 13.2% |
| Somewhat | 62 | 24.2% | 24 | 21.8% | 13 | 21.0% | 1 | 20.0% | 2 | 15.4% | 33 | 18.2% | 64 | 21.7% |
| Fairly | 61 | 23.8% | 27 | 24.5% | 8 | 12.9% | 3 | 60.0% | 1 | 7.7% | 39 | 21.5% | 69 | 23.4% |
| Very | 40 | 15.6% | 27 | 24.5% | 23 | 37.1% | 0 | 0.0% | 4 | 30.8% | 42 | 23.2% | 59 | 20.0% |
| Completely | 16 | 6.3% | 3 | 2.7% | 2 | 3.2% | 0 | 0.0% | 1 | 7.7% | 9 | 5.0% | 17 | 5.8% |
| **Feeling of needing more information or knowledge** | | | | | | | | | | | | | | |
| Yes | 187 | 72.8% | 94 | 85.5% | 47 | 75.8% | 5 | 100.0% | 9 | 69.2% | 141 | 77.9% | 231 | 78.0% |
| No | 70 | 27.2% | 16 | 14.5% | 15 | 24.2% | 0 | 0.0% | 4 | 30.8% | 40 | 22.1% | 65 | 22.0% |

**Table 1** *continued.*

| | Competition levels | | | | | | | | | |
|---|---|---|---|---|---|---|---|---|---|---|
| | Continent Cups (n=197) | | World Cup (n=198) | | Master competitions (n=26) | | Mass events (n=20) | | Snow sports not governed by FIS (n=53) | |
| | **n** | **%** | **n** | **%** | **n** | **%** | **n** | **%** | **n** | **%** |
| **Having enough information** | | | | | | | | | | |
| I don't know | 7 | 4.0% | 3 | 1.7% | 0 | 0.0% | 0 | 0.0% | 3 | 7.1% |
| Not at all | 18 | 10.2% | 24 | 13.4% | 4 | 19.0% | 4 | 25.0% | 7 | 16.7% |
| Slightly | 20 | 11.3% | 26 | 14.5% | 1 | 4.8% | 1 | 6.3% | 7 | 16.7% |
| Somewhat | 43 | 24.3% | 43 | 24.0% | 6 | 28.6% | 4 | 25.0% | 5 | 11.9% |
| Fairly | 52 | 29.4% | 38 | 21.2% | 5 | 23.8% | 2 | 12.5% | 9 | 21.4% |
| Very | 28 | 15.8% | 35 | 19.6% | 5 | 23.8% | 4 | 25.0% | 10 | 23.8% |
| Completely | 9 | 5.1% | 10 | 5.6% | 0 | 0.0% | 1 | 6.3% | 1 | 2.4% |
| **Feeling of needing more information or knowledge** | | | | | | | | | | |
| Yes | 132 | 74.6% | 138 | 77.1% | 16 | 76.2% | 11 | 68.8% | 33 | 78.6% |
| No | 45 | 25.4% | 41 | 22.9% | 5 | 23.8% | 5 | 31.3% | 9 | 21.4% |

**Supplementary file 9**

**Table 1** Needs for more information on injuries.

| | **Total** | | **Snow sports** | | | | | | | | | | | |
|---|---|---|---|---|---|---|---|---|---|---|---|---|---|---|
| | | | **Alpine Skiing (n=205)** | | **Freestyle Skiing (n=93)** | | **Snowboarding (n=72)** | | **Cross-Country Skiing (n=85)** | | **Ski Jumping (n=52)** | | **Nordic Combined (n=31)** | |
| | **n** | **%** | **n** | **%** | **n** | **%** | **n** | **%** | **n** | **%** | **n** | **%** | **n** | **%** |
| **Injury frequent targets** | | | | | | | | | | | | | | |
| Head Injuries & Concussions | **250** | **57.3%** | 129 | 62.9% | 61 | 65.6% | 43 | 59.7% | 29 | 34.1% | 18 | 34.6% | 14 | 45.2% |
| Shoulder, Upper Arm & Elbow Injuries | **115** | **26.4%** | 37 | 18.0% | 26 | 28.0% | 25 | 34.7% | 35 | 41.2% | 9 | 17.3% | 12 | 38.7% |
| Forearm, Wrist & Hand Injuries | **60** | **13.8%** | 25 | 12.2% | 10 | 10.8% | 10 | 13.9% | 15 | 17.6% | 4 | 7.7% | 5 | 16.1% |
| Thoracic Injuries | **58** | **13.3%** | 27 | 13.2% | 11 | 11.8% | 12 | 16.7% | 11 | 12.9% | 5 | 9.6% | 6 | 19.4% |
| Abdominal Injuries | **60** | **13.8%** | 27 | 13.2% | 10 | 10.8% | 10 | 13.9% | 14 | 16.5% | 3 | 5.8% | 4 | 12.9% |
| Spinal Injuries | **163** | **37.4%** | 80 | 39.0% | 34 | 36.6% | 31 | 43.1% | 20 | 23.5% | 13 | 25.0% | 11 | 35.5% |
| Pelvic Injuries | **63** | **14.4%** | 28 | 13.7% | 12 | 12.9% | 14 | 19.4% | 11 | 12.9% | 8 | 15.4% | 4 | 12.9% |
| Hip & Groin Injuries | **96** | **22.0%** | 39 | 19.0% | 16 | 17.2% | 17 | 23.6% | 17 | 20.0% | 12 | 23.1% | 9 | 29.0% |
| Thigh Injuries | **58** | **13.3%** | 26 | 12.7% | 11 | 11.8% | 10 | 13.9% | 10 | 11.8% | 5 | 9.6% | 5 | 16.1% |
| Knee Injuries | **281** | **64.4%** | 132 | 64.4% | 50 | 53.8% | 29 | 40.3% | 35 | 41.2% | 47 | 90.4% | 26 | 83.9% |

| | | | | | | | | | | | | | | |
|---|---|---|---|---|---|---|---|---|---|---|---|---|---|---|
| Lower Leg & Tibia/Shin Injuries | **129** | **29.6%** | 66 | 32.2% | 22 | 23.7% | 14 | 19.4% | 24 | 28.2% | 19 | 36.5% | 10 | 32.3% |
| Ankle, Foot & Toe Injuries | **144** | **33.0%** | 52 | 25.4% | 26 | 28.0% | 22 | 30.6% | 28 | 32.9% | 27 | 51.9% | 13 | 41.9% |
| Other injuries | **26** | **6.0%** | 15 | 7.3% | 5 | 5.4% | 5 | 6.9% | 6 | 7.1% | 0 | 0.0% | 1 | 3.2% |
| None | **37** | **8.5%** | 12 | 5.9% | 9 | 9.7% | 8 | 11.1% | 11 | 12.9% | 2 | 3.8% | 2 | 6.5% |

**Table 1** *continued.*

| | Stakeholder roles | | | | | | | | | | Competition levels | | | |
|---|---|---|---|---|---|---|---|---|---|---|---|---|---|---|
| | Athletes (n=261) | | Coaches (n=116) | | Team staff (n=68) | | Industry - SRS (n=5) | | FIS representatives (n=13) | | Youth competitions (n=205) | | FIS competitions (n=327) | |
| | n | % | n | % | n | % | n | % | n | % | n | % | n | % |
| **Injury frequent targets** | | | | | | | | | | | | | | |
| Head Injuries & Concussions | 140 | 53.6% | 71 | 61.2% | 39 | 57.4% | 4 | 80.0% | 9 | 69.2% | 102 | 49.8% | 169 | 51.7% |
| Shoulder, Upper Arm & Elbow Injuries | 66 | 25.3% | 35 | 30.2% | 21 | 30.9% | 1 | 20.0% | 2 | 15.4% | 51 | 24.9% | 88 | 26.9% |
| Forearm, Wrist & Hand Injuries | 39 | 14.9% | 12 | 10.3% | 9 | 13.2% | 0 | 0.0% | 3 | 23.1% | 27 | 13.2% | 47 | 14.4% |
| Thoracic Injuries | 37 | 14.2% | 16 | 13.8% | 8 | 11.8% | 0 | 0.0% | 2 | 15.4% | 26 | 12.7% | 44 | 13.5% |
| Abdominal Injuries | 40 | 15.3% | 16 | 13.8% | 6 | 8.8% | 0 | 0.0% | 2 | 15.4% | 30 | 14.6% | 45 | 13.8% |
| Spinal Injuries | 91 | 34.9% | 49 | 42.2% | 30 | 44.1% | 2 | 40.0% | 5 | 38.5% | 77 | 37.6% | 121 | 37.0% |
| Pelvic Injuries | 33 | 12.6% | 20 | 17.2% | 12 | 17.6% | 0 | 0.0% | 3 | 23.1% | 31 | 15.1% | 48 | 14.7% |
| Hip & Groin Injuries | 56 | 21.5% | 28 | 24.1% | 16 | 23.5% | 0 | 0.0% | 3 | 23.1% | 46 | 22.4% | 69 | 21.1% |
| Thigh Injuries | 39 | 14.9% | 14 | 12.1% | 6 | 8.8% | 0 | 0.0% | 2 | 15.4% | 29 | 14.1% | 43 | 13.1% |
| Knee Injuries | 172 | 65.9% | 76 | 65.5% | 38 | 55.9% | 5 | 100.0% | 8 | 61.5% | 127 | 62.0% | 204 | 62.4% |
| Lower Leg & Tibia/Shin Injuries | 72 | 27.6% | 40 | 34.5% | 21 | 30.9% | 2 | 40.0% | 4 | 30.8% | 59 | 28.8% | 93 | 28.4% |

| | | | | | | | | | | | | | | |
|---|---|---|---|---|---|---|---|---|---|---|---|---|---|---|
| Ankle, Foot & Toe Injuries | 90 | 34.5% | 33 | 28.4% | 23 | 33.8% | 2 | 40.0% | 3 | 23.1% | 69 | 33.7% | 98 | 30.0% |
| Other injuries | 15 | 5.7% | 9 | 7.8% | 2 | 2.9% | 0 | 0.0% | 1 | 7.7% | 15 | 7.3% | 22 | 6.7% |
| None | 21 | 8.0% | 6 | 5.2% | 8 | 11.8% | 0 | 0.0% | 2 | 15.4% | 14 | 6.8% | 22 | 6.7% |

**Table 1** *continued.*

| | Competition levels | | | | | | | | | |
|---|---|---|---|---|---|---|---|---|---|---|
| | **Continent Cups (n=197)** | | **World Cup (n=198)** | | **Master competitions (n=26)** | | **Mass events (n=20)** | | **Snow sports not governed by FIS (n=53)** | |
| | **n** | **%** | **n** | **%** | **n** | **%** | **n** | **%** | **n** | **%** |
| **Injury frequent targets** | | | | | | | | | | |
| Head Injuries & Concussions | 104 | 52.8% | 118 | 59.6% | 14 | 53.8% | 7 | 35.0% | 25 | 47.2% |
| Shoulder, Upper Arm & Elbow Injuries | 53 | 26.9% | 58 | 29.3% | 7 | 26.9% | 8 | 40.0% | 19 | 35.8% |
| Forearm, Wrist & Hand Injuries | 23 | 11.7% | 24 | 12.1% | 5 | 19.2% | 4 | 20.0% | 9 | 17.0% |
| Thoracic Injuries | 26 | 13.2% | 29 | 14.6% | 3 | 11.5% | 2 | 10.0% | 7 | 13.2% |
| Abdominal Injuries | 25 | 12.7% | 20 | 10.1% | 3 | 11.5% | 1 | 5.0% | 5 | 9.4% |
| Spinal Injuries | 72 | 36.5% | 72 | 36.4% | 9 | 34.6% | 5 | 25.0% | 13 | 24.5% |
| Pelvic Injuries | 26 | 13.2% | 26 | 13.1% | 6 | 23.1% | 2 | 10.0% | 10 | 18.9% |
| Hip & Groin Injuries | 42 | 21.3% | 44 | 22.2% | 5 | 19.2% | 2 | 10.0% | 9 | 17.0% |
| Thigh Injuries | 19 | 9.6% | 25 | 12.6% | 3 | 11.5% | 1 | 5.0% | 5 | 9.4% |
| Knee Injuries | 111 | 56.3% | 110 | 55.6% | 15 | 57.7% | 9 | 45.0% | 25 | 47.2% |
| Lower Leg & Tibia/Shin Injuries | 51 | 25.9% | 48 | 24.2% | 6 | 23.1% | 3 | 15.0% | 13 | 24.5% |

| | | | | | | | | | | |
|---|---|---|---|---|---|---|---|---|---|---|
| Ankle, Foot & Toe Injuries | 54 | 27.4% | 59 | 29.8% | 10 | 38.5% | 5 | 25.0% | 16 | 30.2% |
| Other injuries | 16 | 8.1% | 12 | 6.1% | 3 | 11.5% | 1 | 5.0% | 5 | 9.4% |
| None | 14 | 7.1% | 17 | 8.6% | 3 | 11.5% | 2 | 10.0% | 3 | 5.7% |

**Table 2** Needs for more information on illnesses.

| | Total | | Snow sports | | | | | | | | | | | |
|---|---|---|---|---|---|---|---|---|---|---|---|---|---|---|
| | | | Alpine Skiing (n=205) | | Freestyle Skiing (n=93) | | Snowboarding (n=72) | | Cross-Country Skiing (n=85) | | Ski Jumping (n=52) | | Nordic Combined (n=31) | |
| | **n** | **%** | **n** | **%** | **n** | **%** | **n** | **%** | **n** | **%** | **n** | **%** | **n** | **%** |
| **Illness frequent targets** | | | | | | | | | | | | | | |
| Cardiovascular illnesses | **145** | **33.3%** | 66 | 32.2% | 25 | 26.9% | 20 | 27.8% | 37 | 43.5% | 9 | 17.3% | 11 | 35.5% |
| Endocrinological illnesses | **138** | **31.7%** | 61 | 29.8% | 22 | 23.7% | 21 | 29.2% | 33 | 38.8% | 19 | 36.5% | 8 | 25.8% |
| Respiratory illnesses | **188** | **43.1%** | 81 | 39.5% | 36 | 38.7% | 24 | 33.3% | 48 | 56.5% | 11 | 21.2% | 17 | 54.8% |
| Thermoregulatory illnesses | **134** | **30.7%** | 57 | 27.8% | 22 | 23.7% | 22 | 30.6% | 25 | 29.4% | 15 | 28.8% | 9 | 29.0% |
| Other illnesses | **36** | **8.3%** | 18 | 8.8% | 5 | 5.4% | 6 | 8.3% | 5 | 5.9% | 8 | 15.4% | 5 | 16.1% |
| None | **108** | **24.8%** | 47 | 22.9% | 27 | 29.0% | 20 | 27.8% | 9 | 10.6% | 10 | 19.2% | 4 | 12.9% |

**Table 2** *continued.*

| | Stakeholder roles | | | | | | | | | | Competition levels | | | |
|---|---|---|---|---|---|---|---|---|---|---|---|---|---|---|
| | Athletes (n=261) | | Coaches (n=116) | | Team staff (n=68) | | Industry - SRS (n=5) | | FIS representatives (n=13) | | Youth competitions (n=205) | | FIS competitions (n=327) | |
| | n | % | n | % | n | % | n | % | n | % | n | % | n | % |
| **Illness frequent targets** | | | | | | | | | | | | | | |
| Cardiovascular illnesses | 99 | 37.9% | 35 | 30.2% | 18 | 26.5% | 2 | 40.0% | 4 | 30.8% | 66 | 32.2% | 97 | 29.7% |
| Endocrinological illnesses | 84 | 32.2% | 34 | 29.3% | 24 | 35.3% | 1 | 20.0% | 4 | 30.8% | 60 | 29.3% | 103 | 31.5% |
| Respiratory illnesses | 114 | 43.7% | 48 | 41.4% | 30 | 44.1% | 2 | 40.0% | 6 | 46.2% | 79 | 38.5% | 132 | 40.4% |
| Thermoregulatory illnesses | 87 | 33.3% | 31 | 26.7% | 19 | 27.9% | 1 | 20.0% | 4 | 30.8% | 49 | 23.9% | 89 | 27.2% |
| Other illnesses | 15 | 5.7% | 15 | 12.9% | 6 | 8.8% | 1 | 20.0% | 2 | 15.4% | 21 | 10.2% | 33 | 10.1% |
| None | 66 | 25.3% | 31 | 26.7% | 10 | 14.7% | 1 | 20.0% | 4 | 30.8% | 47 | 22.9% | 73 | 22.3% |

**Table 2** *continued.*

| | Competition levels | | | | | | | | | |
|---|---|---|---|---|---|---|---|---|---|---|
| | Continent Cups (n=197) | | World Cup (n=198) | | Master competitions (n=26) | | Mass events (n=20) | | Snow sports not governed by FIS (n=53) | |
| | n | % | n | % | n | % | n | % | n | % |
| **Illness frequent targets** | | | | | | | | | | |
| Cardiovascular illnesses | 67 | 34.0% | 68 | 34.3% | 10 | 38.5% | 7 | 35.0% | 15 | 28.3% |
| Endocrinological illnesses | 60 | 30.5% | 59 | 29.8% | 5 | 19.2% | 6 | 30.0% | 14 | 26.4% |
| Respiratory illnesses | 81 | 41.1% | 81 | 40.9% | 11 | 42.3% | 10 | 50.0% | 18 | 34.0% |
| Thermoregulatory illnesses | 52 | 26.4% | 55 | 27.8% | 8 | 30.8% | 8 | 40.0% | 15 | 28.3% |
| Other illnesses | 19 | 9.6% | 12 | 6.1% | 2 | 7.7% | 1 | 5.0% | 6 | 11.3% |
| None | 43 | 21.8% | 48 | 24.2% | 2 | 7.7% | 2 | 10.0% | 12 | 22.6% |

## Supplementary file 10

**Table 1.A** Needs for more information on targeted prevention areas: overall, athlete- and equipment-related areas.

| | **Total** | | **Snow sports** | | | | | | | | | | | |
|---|---|---|---|---|---|---|---|---|---|---|---|---|---|---|
| | | | **Alpine Skiing (n=205)** | | **Freestyle Skiing (n=93)** | | **Snowboarding (n=72)** | | **Cross-Country Skiing (n=85)** | | **Ski Jumping (n=52)** | | **Nordic Combined (n=31)** | |
| | **n** | **%** | **n** | **%** | **n** | **%** | **n** | **%** | **n** | **%** | **n** | **%** | **n** | **%** |
| **Frequent targeted areas** | | | | | | | | | | | | | | |
| **A. Athlete-related areas** | **1206** | **32.0%** | 556 | 31.4% | 233 | 32.2% | 178 | 32.7% | 214 | 35.3% | 149 | 31.6% | 92 | 30.8% |
| Physical aspects | **280** | **64.2%** | 125 | 61.0% | 54 | 58.1% | 38 | 52.8% | 55 | 64.7% | 31 | 59.6% | 22 | 71.0% |
| Mental aspects | **299** | **68.6%** | 127 | 62.0% | 61 | 65.6% | 45 | 62.5% | 52 | 61.2% | 40 | 76.9% | 22 | 71.0% |
| Skill/technical/tactical aspects | **176** | **40.4%** | 80 | 39.0% | 32 | 34.4% | 24 | 33.3% | 25 | 29.4% | 34 | 65.4% | 12 | 38.7% |
| Load management | **158** | **36.2%** | 85 | 41.5% | 27 | 29.0% | 22 | 30.6% | 29 | 34.1% | 14 | 26.9% | 15 | 48.4% |
| Education/awareness | **148** | **33.9%** | 76 | 37.1% | 29 | 31.2% | 25 | 34.7% | 21 | 24.7% | 15 | 28.8% | 10 | 32.3% |
| Social aspects | **114** | **26.1%** | 51 | 24.9% | 22 | 23.7% | 17 | 23.6% | 25 | 29.4% | 13 | 25.0% | 10 | 32.3% |
| Others | **5** | **1.1%** | 4 | 2.0% | 1 | 1.1% | 2 | 2.8% | 1 | 1.2% | 1 | 1.9% | 1 | 3.2% |
| None | **26** | **6.0%** | 8 | 3.9% | 7 | 7.5% | 5 | 6.9% | 6 | 7.1% | 1 | 1.9% | 0 | 0.0% |

| | | | | | | | | | | | | | | |
|---|---|---|---|---|---|---|---|---|---|---|---|---|---|---|
| **B. Equipment-related areas** | **938** | **24.9%** | 462 | 26.1% | 163 | 22.5% | 119 | 21.9% | 124 | 20.4% | 136 | 28.8% | 81 | 27.1% |
| Ski/board (including preparation) | **208** | **47.7%** | 99 | 48.3% | 31 | 33.3% | 20 | 27.8% | 36 | 42.4% | 32 | 61.5% | 20 | 64.5% |
| Binding | **172** | **39.4%** | 91 | 44.4% | 24 | 25.8% | 18 | 25.0% | 10 | 11.8% | 37 | 71.2% | 22 | 71.0% |
| Boot | **153** | **35.1%** | 77 | 37.6% | 23 | 24.7% | 15 | 20.8% | 19 | 22.4% | 28 | 53.8% | 13 | 41.9% |
| Protectors/helmet | **185** | **42.4%** | 102 | 49.8% | 40 | 43.0% | 32 | 44.4% | 11 | 12.9% | 16 | 30.8% | 11 | 35.5% |
| Gear/clothing | **121** | **27.8%** | 60 | 29.3% | 25 | 26.9% | 16 | 22.2% | 19 | 22.4% | 17 | 32.7% | 12 | 38.7% |
| Others | **9** | **2.1%** | 6 | 2.9% | 4 | 4.3% | 3 | 4.2% | 1 | 1.2% | 1 | 1.9% | 0 | 0.0% |
| None | **90** | **20.6%** | 27 | 13.2% | 16 | 17.2% | 15 | 20.8% | 28 | 32.9% | 5 | 9.6% | 3 | 9.7% |

**Table 1.A** *continued.*

| | Stakeholder roles | | | | | | | | | | Competition levels | | | |
|---|---|---|---|---|---|---|---|---|---|---|---|---|---|---|
| | Athletes (n=261) | | Coaches (n=116) | | Team staff (n=68) | | Industry - SRS (n=5) | | FIS representatives (n=13) | | Youth competitions (n=205) | | FIS competitions (n=327) | |
| | n | % | n | % | n | % | n | % | n | % | n | % | n | % |
| **Frequent targeted areas** | | | | | | | | | | | | | | |
| **A. Athlete-related areas** | 706 | 31.9% | 342 | 32.5% | 195 | 32.8% | 14 | 26.4% | 39 | 32.8% | 555 | 33.4% | 890 | 33.1% |
| Physical aspects | 170 | 65.1% | 75 | 64.7% | 45 | 66.2% | 5 | 100.0% | 9 | 69.2% | 134 | 65.4% | 205 | 62.7% |
| Mental aspects | 182 | 69.7% | 78 | 67.2% | 49 | 72.1% | 2 | 40.0% | 9 | 69.2% | 131 | 63.9% | 221 | 67.6% |
| Skill/technical/tactical aspects | 116 | 44.4% | 39 | 33.6% | 26 | 38.2% | 2 | 40.0% | 3 | 23.1% | 83 | 40.5% | 139 | 42.5% |
| Load management | 79 | 30.3% | 54 | 46.6% | 29 | 42.6% | 2 | 40.0% | 5 | 38.5% | 72 | 35.1% | 110 | 33.6% |
| Education/awareness | 73 | 28.0% | 54 | 46.6% | 26 | 38.2% | 2 | 40.0% | 7 | 53.8% | 71 | 34.6% | 111 | 33.9% |
| Social aspects | 63 | 24.1% | 38 | 32.8% | 18 | 26.5% | 0 | 0.0% | 5 | 38.5% | 55 | 26.8% | 88 | 26.9% |
| Others | 2 | 0.8% | 1 | 0.9% | 1 | 1.5% | 1 | 20.0% | 0 | 0.0% | 2 | 1.0% | 5 | 1.5% |
| None | 21 | 8.0% | 3 | 2.6% | 1 | 1.5% | 0 | 0.0% | 1 | 7.7% | 7 | 3.4% | 11 | 3.4% |
| **B. Equipment-related areas** | 552 | 24.9% | 256 | 24.4% | 138 | 23.2% | 20 | 37.7% | 28 | 23.5% | 412 | 24.8% | 662 | 24.6% |
| Ski/board (including preparation) | 126 | 48.3% | 53 | 45.7% | 30 | 44.1% | 5 | 100.0% | 7 | 53.8% | 94 | 45.9% | 156 | 47.7% |

| | | | | | | | | | | | | | | |
|---|---|---|---|---|---|---|---|---|---|---|---|---|---|---|
| Binding | 90 | 34.5% | 55 | 47.4% | 30 | 44.1% | 5 | 100.0% | 5 | 38.5% | 81 | 39.5% | 117 | 35.8% |
| Boot | 84 | 32.2% | 45 | 38.8% | 27 | 39.7% | 4 | 80.0% | 6 | 46.2% | 71 | 34.6% | 107 | 32.7% |
| Protectors/helmet | 112 | 42.9% | 50 | 43.1% | 21 | 30.9% | 4 | 80.0% | 4 | 30.8% | 79 | 38.5% | 127 | 38.8% |
| Gear/clothing | 76 | 29.1% | 33 | 28.4% | 15 | 22.1% | 2 | 40.0% | 2 | 15.4% | 54 | 26.3% | 85 | 26.0% |
| Others | 4 | 1.5% | 5 | 4.3% | 2 | 2.9% | 0 | 0.0% | 1 | 7.7% | 3 | 1.5% | 7 | 2.1% |
| None | 60 | 23.0% | 15 | 12.9% | 13 | 19.1% | 0 | 0.0% | 3 | 23.1% | 30 | 14.6% | 63 | 19.3% |

**Table 1.A** *continued.*

| | **Competition levels** | | | | | | | | | |
|---|---|---|---|---|---|---|---|---|---|---|
| | **Continent Cups (n=197)** | | **World Cup (n=198)** | | **Master competitions (n=26)** | | **Mass events (n=20)** | | **Snow sports not governed by FIS (n=53)** | |
| | **n** | **%** | **n** | **%** | **n** | **%** | **n** | **%** | **n** | **%** |
| **Frequent targeted areas** | | | | | | | | | | |
| **A. Athlete-related areas** | 524 | 33.0% | 512 | 31.5% | 62 | 28.4% | 52 | 32.1% | 119 | 32.2% |
| Physical aspects | 116 | 58.9% | 112 | 56.6% | 13 | 50.0% | 11 | 55.0% | 26 | 49.1% |
| Mental aspects | 126 | 64.0% | 125 | 63.1% | 13 | 50.0% | 15 | 75.0% | 28 | 52.8% |
| Skill/technical/tactical aspects | 76 | 38.6% | 67 | 33.8% | 10 | 38.5% | 4 | 20.0% | 19 | 35.8% |
| Load management | 69 | 35.0% | 78 | 39.4% | 7 | 26.9% | 7 | 35.0% | 18 | 34.0% |
| Education/awareness | 71 | 36.0% | 65 | 32.8% | 10 | 38.5% | 4 | 20.0% | 14 | 26.4% |
| Social aspects | 52 | 26.4% | 47 | 23.7% | 7 | 26.9% | 10 | 50.0% | 11 | 20.8% |
| Others | 3 | 1.5% | 3 | 1.5% | 0 | 0.0% | 0 | 0.0% | 0 | 0.0% |
| None | 11 | 5.6% | 15 | 7.6% | 2 | 7.7% | 1 | 5.0% | 3 | 5.7% |
| **B. Equipment-related areas** | 393 | 24.8% | 397 | 24.5% | 55 | 25.2% | 40 | 24.7% | 84 | 22.7% |
| Ski/board (including preparation) | 90 | 45.7% | 85 | 42.9% | 11 | 42.3% | 11 | 55.0% | 17 | 32.1% |
| Binding | 70 | 35.5% | 76 | 38.4% | 11 | 42.3% | 6 | 30.0% | 13 | 24.5% |
| Boot | 64 | 32.5% | 62 | 31.3% | 10 | 38.5% | 7 | 35.0% | 13 | 24.5% |

| | | | | | | | | | | |
|---|---|---|---|---|---|---|---|---|---|---|
| Protectors/helmet | 75 | 38.1% | 82 | 41.4% | 13 | 50.0% | 6 | 30.0% | 19 | 35.8% |
| Gear/clothing | 49 | 24.9% | 50 | 25.3% | 6 | 23.1% | 6 | 30.0% | 12 | 22.6% |
| Others | 4 | 2.0% | 5 | 2.5% | 1 | 3.8% | 0 | 0.0% | 3 | 5.7% |
| None | 41 | 20.8% | 37 | 18.7% | 3 | 11.5% | 4 | 20.0% | 7 | 13.2% |

**Table 1.B** Needs for more information on targeted areas: course- and snow/environment-related areas.

| | Total | | Snow sports | | | | | | | | | | | |
|---|---|---|---|---|---|---|---|---|---|---|---|---|---|---|
| | | | Alpine Skiing (n=205) | | Freestyle Skiing (n=93) | | Snowboarding (n=72) | | Cross-Country Skiing (n=85) | | Ski Jumping (n=52) | | Nordic Combined (n=31) | |
| | n | % | n | % | n | % | n | % | n | % | n | % | n | % |
| **C. Course-related areas** | **775** | **20.6%** | 382 | 21.5% | 165 | 22.8% | 120 | 22.1% | 116 | 19.1% | 81 | 17.2% | 58 | 19.4% |
| Course design | **231** | **53.0%** | 123 | 60.0% | 45 | 48.4% | 38 | 52.8% | 39 | 45.9% | 16 | 30.8% | 12 | 38.7% |
| Jump design | **154** | **35.3%** | 61 | 29.8% | 45 | 48.4% | 28 | 38.9% | 7 | 8.2% | 28 | 53.8% | 17 | 54.8% |
| Safety nets | **126** | **28.9%** | 81 | 39.5% | 26 | 28.0% | 20 | 27.8% | 14 | 16.5% | 8 | 15.4% | 9 | 29.0% |
| Jury decisions | **160** | **36.7%** | 83 | 40.5% | 32 | 34.4% | 18 | 25.0% | 29 | 34.1% | 18 | 34.6% | 11 | 35.5% |
| Others | **4** | **0.9%** | 3 | 1.5% | 0 | 0.0% | 0 | 0.0% | 1 | 1.2% | 0 | 0.0% | 1 | 3.2% |
| None | **100** | **22.9%** | 31 | 15.1% | 17 | 18.3% | 16 | 22.2% | 26 | 30.6% | 11 | 21.2% | 8 | 25.8% |
| **D. Snow- and environment-related areas** | **846** | **22.5%** | 373 | 21.0% | 163 | 22.5% | 127 | 23.3% | 153 | 25.2% | 106 | 22.5% | 68 | 22.7% |
| Snow surface | **258** | **59.2%** | 127 | 62.0% | 41 | 44.1% | 35 | 48.6% | 43 | 50.6% | 33 | 63.5% | 21 | 67.7% |
| Visibility | **179** | **41.1%** | 95 | 46.3% | 40 | 43.0% | 28 | 38.9% | 20 | 23.5% | 17 | 32.7% | 11 | 35.5% |
| Temperature | **138** | **31.7%** | 57 | 27.8% | 21 | 22.6% | 20 | 27.8% | 42 | 49.4% | 14 | 26.9% | 10 | 32.3% |
| Wind | **170** | **39.0%** | 52 | 25.4% | 36 | 38.7% | 26 | 36.1% | 30 | 35.3% | 37 | 71.2% | 22 | 71.0% |
| Others | **10** | **2.3%** | 6 | 2.9% | 3 | 3.2% | 1 | 1.4% | 1 | 1.2% | 1 | 1.9% | 1 | 3.2% |

| | | | | | | | | | | | | | | |
|---|---|---|---|---|---|---|---|---|---|---|---|---|---|---|
| None | **91** | **20.9%** | 36 | 17.6% | 22 | 23.7% | 17 | 23.6% | 17 | 20.0% | 4 | 7.7% | 3 | 9.7% |

**Table 1.B** *continued.*

| | Stakeholder roles | | | | | | | | | | Competition levels | | | |
|---|---|---|---|---|---|---|---|---|---|---|---|---|---|---|
| | Athletes (n=261) | | Coaches (n=116) | | Team staff (n=68) | | Industry - SRS (n=5) | | FIS representatives (n=13) | | Youth competitions (n=205) | | FIS competitions (n=327) | |
| | n | % | n | % | n | % | n | % | n | % | n | % | n | % |
| **C. Course-related areas** | 455 | 20.6% | 226 | 21.5% | 116 | 19.5% | 10 | 18.9% | 25 | 21.0% | 330 | 19.9% | 551 | 20.5% |
| Course design | 134 | 51.3% | 73 | 62.9% | 33 | 48.5% | 5 | 100.0% | 7 | 53.8% | 103 | 50.2% | 160 | 48.9% |
| Jump design | 85 | 32.6% | 50 | 43.1% | 24 | 35.3% | 2 | 40.0% | 3 | 23.1% | 62 | 30.2% | 112 | 34.3% |
| Safety nets | 68 | 26.1% | 41 | 35.3% | 20 | 29.4% | 1 | 20.0% | 7 | 53.8% | 48 | 23.4% | 86 | 26.3% |
| Jury decisions | 100 | 38.3% | 45 | 38.8% | 22 | 32.4% | 2 | 40.0% | 5 | 38.5% | 74 | 36.1% | 118 | 36.1% |
| Others | 1 | 0.4% | 2 | 1.7% | 1 | 1.5% | 0 | 0.0% | 0 | 0.0% | 1 | 0.5% | 4 | 1.2% |
| None | 67 | 25.7% | 15 | 12.9% | 16 | 23.5% | 0 | 0.0% | 3 | 23.1% | 42 | 20.5% | 71 | 21.7% |
| **D. Snow- and environment-related areas** | 500 | 22.6% | 227 | 21.6% | 145 | 24.4% | 9 | 17.0% | 27 | 22.7% | 365 | 22.0% | 589 | 21.9% |
| Snow surface | 145 | 55.6% | 75 | 64.7% | 45 | 66.2% | 5 | 100.0% | 8 | 61.5% | 117 | 57.1% | 178 | 54.4% |
| Visibility | 110 | 42.1% | 44 | 37.9% | 31 | 45.6% | 2 | 40.0% | 6 | 46.2% | 71 | 34.6% | 126 | 38.5% |
| Temperature | 78 | 29.9% | 38 | 32.8% | 29 | 42.6% | 2 | 40.0% | 5 | 38.5% | 67 | 32.7% | 98 | 30.0% |
| Wind | 104 | 39.8% | 43 | 37.1% | 31 | 45.6% | 0 | 0.0% | 4 | 30.8% | 72 | 35.1% | 114 | 34.9% |
| Others | 4 | 1.5% | 4 | 3.4% | 2 | 2.9% | 0 | 0.0% | 0 | 0.0% | 4 | 2.0% | 9 | 2.8% |

| | | | | | | | | | | | | | | |
|---|---|---|---|---|---|---|---|---|---|---|---|---|---|---|
| None | 59 | 22.6% | 23 | 19.8% | 7 | 10.3% | 0 | 0.0% | 4 | 30.8% | 34 | 16.6% | 64 | 19.6% |

**Table 1.B** *continued.*

| | Competition levels | | | | | | | | | |
|---|---|---|---|---|---|---|---|---|---|---|
| | Continent Cups (n=197) | | World Cup (n=198) | | Master competitions (n=26) | | Mass events (n=20) | | Snow sports not governed by FIS (n=53) | |
| | **n** | **%** | **n** | **%** | **n** | **%** | **n** | **%** | **n** | **%** |
| **C. Course-related areas** | 323 | 20.4% | 341 | 21.0% | 49 | 22.5% | 32 | 19.8% | 77 | 20.8% |
| Course design | 91 | 46.2% | 99 | 50.0% | 17 | 65.4% | 9 | 45.0% | 23 | 43.4% |
| Jump design | 71 | 36.0% | 74 | 37.4% | 9 | 34.6% | 7 | 35.0% | 15 | 28.3% |
| Safety nets | 46 | 23.4% | 58 | 29.3% | 10 | 38.5% | 4 | 20.0% | 11 | 20.8% |
| Jury decisions | 71 | 36.0% | 65 | 32.8% | 10 | 38.5% | 9 | 45.0% | 14 | 26.4% |
| Others | 1 | 0.5% | 2 | 1.0% | 1 | 3.8% | 0 | 0.0% | 1 | 1.9% |
| None | 43 | 21.8% | 43 | 21.7% | 2 | 7.7% | 3 | 15.0% | 13 | 24.5% |
| **D. Snow- and environment-related areas** | 347 | 21.9% | 373 | 23.0% | 52 | 23.9% | 38 | 23.5% | 90 | 24.3% |
| Snow surface | 107 | 54.3% | 110 | 55.6% | 13 | 50.0% | 12 | 60.0% | 24 | 45.3% |
| Visibility | 73 | 37.1% | 82 | 41.4% | 14 | 53.8% | 8 | 40.0% | 18 | 34.0% |
| Temperature | 53 | 26.9% | 55 | 27.8% | 9 | 34.6% | 6 | 30.0% | 14 | 26.4% |
| Wind | 67 | 34.0% | 84 | 42.4% | 11 | 42.3% | 9 | 45.0% | 23 | 43.4% |
| Others | 4 | 2.0% | 5 | 2.5% | 1 | 3.8% | 1 | 5.0% | 2 | 3.8% |
| None | 43 | 21.8% | 37 | 18.7% | 4 | 15.4% | 2 | 10.0% | 9 | 17.0% |

## Supplementary file 11

**Table 1.A** Needs for more information on specific preventative intervention areas: injury and illness registration methods and warm-up, activation and cool-down strategies.

| | **Total** | | **Snow sports** | | | | | | | | | | | |
|---|---|---|---|---|---|---|---|---|---|---|---|---|---|---|
| | | | **Alpine Skiing (n=205)** | | **Freestyle Skiing (n=93)** | | **Snowboarding (n=72)** | | **Cross-Country Skiing (n=85)** | | **Ski Jumping (n=52)** | | **Nordic Combined (n=31)** | |
| | **n** | **%** | **n** | **%** | **n** | **%** | **n** | **%** | **n** | **%** | **n** | **%** | **n** | **%** |
| **A. Injury and illness registration methods** | | | | | | | | | | | | | | |
| I don't know | **82** | **19.9%** | 29 | 16.5% | 22 | 27.5% | 14 | 24.1% | 8 | 10.4% | 8 | 16.0% | 4 | 13.3% |
| Not at all | **71** | **17.2%** | 24 | 13.6% | 16 | 20.0% | 10 | 17.2% | 17 | 22.1% | 4 | 8.0% | 5 | 16.7% |
| Slightly | **42** | **10.2%** | 13 | 7.4% | 6 | 7.5% | 7 | 12.1% | 11 | 14.3% | 10 | 20.0% | 6 | 20.0% |
| Somewhat | **61** | **14.8%** | 34 | 19.3% | 9 | 11.3% | 6 | 10.3% | 12 | 15.6% | 4 | 8.0% | 2 | 6.7% |
| Fairly | **67** | **16.2%** | 32 | 18.2% | 11 | 13.8% | 10 | 17.2% | 11 | 14.3% | 14 | 28.0% | 5 | 16.7% |
| Very | **59** | **14.3%** | 28 | 15.9% | 11 | 13.8% | 5 | 8.6% | 10 | 13.0% | 8 | 16.0% | 8 | 26.7% |
| Completely | **31** | **7.5%** | 16 | 9.1% | 5 | 6.3% | 6 | 10.3% | 8 | 10.4% | 2 | 4.0% | 0 | 0.0% |
| **B. Warm-up, activation and cool-down strategies** | | | | | | | | | | | | | | |
| I don't know | **23** | **5.6%** | 9 | 5.1% | 5 | 6.3% | 2 | 3.4% | 2 | 2.6% | 2 | 4.0% | 3 | 10.0% |
| Not at all | **95** | **23.1%** | 32 | 18.3% | 22 | 27.5% | 21 | 36.2% | 16 | 20.8% | 8 | 16.0% | 8 | 26.7% |
| Slightly | **63** | **15.3%** | 34 | 19.4% | 13 | 16.3% | 10 | 17.2% | 14 | 18.2% | 10 | 20.0% | 7 | 23.3% |
| Somewhat | **75** | **18.2%** | 31 | 17.7% | 16 | 20.0% | 13 | 22.4% | 13 | 16.9% | 7 | 14.0% | 3 | 10.0% |

| | | | | | | | | | | | | | | |
|---|---|---|---|---|---|---|---|---|---|---|---|---|---|---|
| Fairly | **79** | **19.2%** | 36 | 20.6% | 11 | 13.8% | 5 | 8.6% | 15 | 19.5% | 10 | 20.0% | 4 | 13.3% |
| Very | **33** | **8.0%** | 12 | 6.9% | 7 | 8.8% | 1 | 1.7% | 7 | 9.1% | 6 | 12.0% | 5 | 16.7% |
| Completely | **44** | **10.7%** | 21 | 12.0% | 6 | 7.5% | 6 | 10.3% | 10 | 13.0% | 7 | 14.0% | 0 | 0.0% |

**Table 1.A** *continued.*

| | **Stakeholder roles** | | | | | | | | | | **Competition levels** | | | |
|---|---|---|---|---|---|---|---|---|---|---|---|---|---|---|
| | **Athletes (n=261)** | | **Coaches (n=116)** | | **Team staff (n=68)** | | **Industry - SRS (n=5)** | | **FIS representatives (n=13)** | | **Youth competitions (n=205)** | | **FIS competitions (n=327)** | |
| | **n** | **%** | **n** | **%** | **n** | **%** | **n** | **%** | **n** | **%** | **n** | **%** | **n** | **%** |
| **A. Injury and illness registration methods** | | | | | | | | | | | | | | |
| I don't know | 62 | 24.7% | 14 | 12.8% | 8 | 13.3% | 1 | 20.0% | 2 | 15.4% | 32 | 17.9% | 57 | 19.5% |
| Not at all | 40 | 15.9% | 20 | 18.3% | 11 | 18.3% | 0 | 0.0% | 2 | 15.4% | 30 | 16.8% | 45 | 15.4% |
| Slightly | 25 | 10.0% | 14 | 12.8% | 7 | 11.7% | 0 | 0.0% | 1 | 7.7% | 16 | 8.9% | 32 | 10.9% |
| Somewhat | 34 | 13.5% | 20 | 18.3% | 7 | 11.7% | 1 | 20.0% | 0 | 0.0% | 30 | 16.8% | 45 | 15.4% |
| Fairly | 37 | 14.7% | 19 | 17.4% | 11 | 18.3% | 2 | 40.0% | 2 | 15.4% | 26 | 14.5% | 50 | 17.1% |
| Very | 34 | 13.5% | 13 | 11.9% | 12 | 20.0% | 1 | 20.0% | 4 | 30.8% | 25 | 14.0% | 42 | 14.3% |
| Completely | 19 | 7.6% | 9 | 8.3% | 4 | 6.7% | 0 | 0.0% | 2 | 15.4% | 20 | 11.2% | 22 | 7.5% |
| **B. Warm-up, activation and cool-down strategies** | | | | | | | | | | | | | | |
| I don't know | 19 | 7.5% | 2 | 1.9% | 1 | 1.6% | 1 | 20.0% | 0 | 0.0% | 8 | 4.5% | 15 | 5.2% |
| Not at all | 58 | 22.9% | 23 | 21.9% | 14 | 23.0% | 0 | 0.0% | 4 | 30.8% | 29 | 16.4% | 61 | 21.0% |
| Slightly | 31 | 12.3% | 19 | 18.1% | 14 | 23.0% | 1 | 20.0% | 5 | 38.5% | 24 | 13.6% | 48 | 16.6% |
| Somewhat | 45 | 17.8% | 21 | 20.0% | 12 | 19.7% | 0 | 0.0% | 1 | 7.7% | 38 | 21.5% | 56 | 19.3% |
| Fairly | 50 | 19.8% | 19 | 18.1% | 12 | 19.7% | 1 | 20.0% | 1 | 7.7% | 37 | 20.9% | 55 | 19.0% |
| Very | 21 | 8.3% | 9 | 8.6% | 2 | 3.3% | 2 | 40.0% | 0 | 0.0% | 20 | 11.3% | 22 | 7.6% |
| Completely | 29 | 11.5% | 12 | 11.4% | 6 | 9.8% | 0 | 0.0% | 2 | 15.4% | 21 | 11.9% | 33 | 11.4% |

**Table 1.A** *continued.*

| | Competition levels | | | | | | | | | |
|---|---|---|---|---|---|---|---|---|---|---|
| | Continent Cups (n=197) | | World Cup (n=198) | | Master competitions (n=26) | | Mass events (n=20) | | Snow sports not governed by FIS (n=53) | |
| | n | % | n | % | n | % | n | % | n | % |
| **A. Injury and illness registration methods** | | | | | | | | | | |
| I don't know | 35 | 20.0% | 32 | 18.3% | 3 | 15.0% | 1 | 6.3% | 6 | 14.6% |
| Not at all | 26 | 14.9% | 33 | 18.9% | 6 | 30.0% | 2 | 12.5% | 7 | 17.1% |
| Slightly | 23 | 13.1% | 19 | 10.9% | 2 | 10.0% | 3 | 18.8% | 6 | 14.6% |
| Somewhat | 29 | 16.6% | 21 | 12.0% | 3 | 15.0% | 1 | 6.3% | 7 | 17.1% |
| Fairly | 31 | 17.7% | 30 | 17.1% | 0 | 0.0% | 4 | 25.0% | 5 | 12.2% |
| Very | 24 | 13.7% | 29 | 16.6% | 2 | 10.0% | 4 | 25.0% | 6 | 14.6% |
| Completely | 7 | 4.0% | 11 | 6.3% | 4 | 20.0% | 1 | 6.3% | 4 | 9.8% |
| **B. Warm-up, activation and cool-down strategies** | | | | | | | | | | |
| I don't know | 6 | 3.4% | 8 | 4.6% | 0 | 0.0% | 0 | 0.0% | 2 | 4.9% |
| Not at all | 42 | 24.1% | 44 | 25.1% | 3 | 15.0% | 5 | 31.3% | 9 | 22.0% |
| Slightly | 35 | 20.1% | 30 | 17.1% | 3 | 15.0% | 4 | 25.0% | 5 | 12.2% |
| Somewhat | 35 | 20.1% | 35 | 20.0% | 5 | 25.0% | 2 | 12.5% | 11 | 26.8% |
| Fairly | 30 | 17.2% | 32 | 18.3% | 4 | 20.0% | 1 | 6.3% | 2 | 4.9% |
| Very | 12 | 6.9% | 13 | 7.4% | 0 | 0.0% | 1 | 6.3% | 6 | 14.6% |
| Completely | 14 | 8.0% | 13 | 7.4% | 5 | 25.0% | 3 | 18.8% | 6 | 14.6% |

**Table 1.B** Needs for more information on specific intervention areas: training methods and testing practices.

| | Total | | Snow sports | | | | | | | | | | | |
|---|---|---|---|---|---|---|---|---|---|---|---|---|---|---|
| | | | Alpine Skiing (n=205) | | Freestyle Skiing (n=93) | | Snowboarding (n=72) | | Cross-Country Skiing (n=85) | | Ski Jumping (n=52) | | Nordic Combined (n=31) | |
| | n | % | n | % | n | % | n | % | n | % | n | % | n | % |
| **C. Training methods** | | | | | | | | | | | | | | |
| I don't know | **33** | **8.0%** | 16 | 9.1% | 3 | 3.8% | 3 | 5.2% | 6 | 7.8% | 1 | 2.0% | 4 | 13.8% |
| Not at all | **67** | **16.3%** | 21 | 11.9% | 19 | 23.8% | 11 | 19.0% | 14 | 18.2% | 6 | 12.0% | 7 | 24.1% |
| Slightly | **45** | **10.9%** | 21 | 11.9% | 12 | 15.0% | 7 | 12.1% | 9 | 11.7% | 6 | 12.0% | 4 | 13.8% |
| Somewhat | **87** | **21.1%** | 42 | 23.9% | 15 | 18.8% | 13 | 22.4% | 12 | 15.6% | 12 | 24.0% | 1 | 3.4% |
| Fairly | **73** | **17.7%** | 35 | 19.9% | 15 | 18.8% | 7 | 12.1% | 11 | 14.3% | 9 | 18.0% | 5 | 17.2% |
| Very | **57** | **13.8%** | 22 | 12.5% | 9 | 11.3% | 9 | 15.5% | 11 | 14.3% | 8 | 16.0% | 7 | 24.1% |
| Completely | **50** | **12.1%** | 19 | 10.8% | 7 | 8.8% | 8 | 13.8% | 14 | 18.2% | 8 | 16.0% | 1 | 3.4% |
| **D. Testing practices** | | | | | | | | | | | | | | |
| I don't know | **71** | **17.3%** | 31 | 17.8% | 14 | 17.5% | 9 | 15.5% | 9 | 11.7% | 8 | 16.0% | 3 | 10.3% |
| Not at all | **82** | **20.0%** | 24 | 13.8% | 23 | 28.8% | 13 | 22.4% | 19 | 24.7% | 7 | 14.0% | 6 | 20.7% |
| Slightly | **42** | **10.2%** | 22 | 12.6% | 8 | 10.0% | 7 | 12.1% | 14 | 18.2% | 4 | 8.0% | 4 | 13.8% |
| Somewhat | **67** | **16.3%** | 31 | 17.8% | 12 | 15.0% | 10 | 17.2% | 10 | 13.0% | 8 | 16.0% | 3 | 10.3% |
| Fairly | **67** | **16.3%** | 34 | 19.5% | 9 | 11.3% | 3 | 5.2% | 7 | 9.1% | 15 | 30.0% | 5 | 17.2% |
| Very | **47** | **11.5%** | 17 | 9.8% | 8 | 10.0% | 10 | 17.2% | 8 | 10.4% | 5 | 10.0% | 8 | 27.6% |
| Completely | **34** | **8.3%** | 15 | 8.6% | 6 | 7.5% | 6 | 10.3% | 10 | 13.0% | 3 | 6.0% | 0 | 0.0% |

**Table 1.B** *continued.*

| | Stakeholder roles | | | | | | | | | | Competition levels | | | |
|---|---|---|---|---|---|---|---|---|---|---|---|---|---|---|
| | Athletes (n=261) | | Coaches (n=116) | | Team staff (n=68) | | Industry - SRS (n=5) | | FIS representatives (n=13) | | Youth competitions (n=205) | | FIS competitions (n=327) | |
| | n | % | n | % | n | % | n | % | n | % | n | % | n | % |
| **C. Training methods** | | | | | | | | | | | | | | |
| I don't know | 30 | 11.9% | 0 | 0.0% | 2 | 3.3% | 1 | 20.0% | 0 | 0.0% | 14 | 7.8% | 23 | 7.9% |
| Not at all | 40 | 15.9% | 16 | 15.1% | 12 | 19.7% | 1 | 20.0% | 4 | 30.8% | 24 | 13.4% | 40 | 13.7% |
| Slightly | 22 | 8.7% | 15 | 14.2% | 8 | 13.1% | 1 | 20.0% | 2 | 15.4% | 17 | 9.5% | 31 | 10.7% |
| Somewhat | 50 | 19.8% | 28 | 26.4% | 13 | 21.3% | 0 | 0.0% | 2 | 15.4% | 38 | 21.2% | 62 | 21.3% |
| Fairly | 43 | 17.1% | 23 | 21.7% | 9 | 14.8% | 0 | 0.0% | 1 | 7.7% | 38 | 21.2% | 54 | 18.6% |
| Very | 34 | 13.5% | 14 | 13.2% | 10 | 16.4% | 2 | 40.0% | 2 | 15.4% | 30 | 16.8% | 43 | 14.8% |
| Completely | 33 | 13.1% | 10 | 9.4% | 7 | 11.5% | 0 | 0.0% | 2 | 15.4% | 18 | 10.1% | 38 | 13.1% |
| **D. Testing practices** | | | | | | | | | | | | | | |
| I don't know | 55 | 21.9% | 10 | 9.6% | 3 | 5.0% | 2 | 40.0% | 1 | 7.7% | 30 | 16.9% | 55 | 19.0% |
| Not at all | 60 | 23.9% | 12 | 11.5% | 12 | 20.0% | 0 | 0.0% | 3 | 23.1% | 30 | 16.9% | 58 | 20.1% |
| Slightly | 22 | 8.8% | 16 | 15.4% | 8 | 13.3% | 0 | 0.0% | 1 | 7.7% | 17 | 9.6% | 28 | 9.7% |
| Somewhat | 36 | 14.3% | 23 | 22.1% | 9 | 15.0% | 0 | 0.0% | 3 | 23.1% | 25 | 14.1% | 40 | 13.8% |
| Fairly | 34 | 13.5% | 23 | 22.1% | 11 | 18.3% | 2 | 40.0% | 0 | 0.0% | 34 | 19.2% | 48 | 16.6% |
| Very | 25 | 10.0% | 10 | 9.6% | 11 | 18.3% | 1 | 20.0% | 3 | 23.1% | 23 | 13.0% | 33 | 11.4% |
| Completely | 19 | 7.6% | 10 | 9.6% | 6 | 10.0% | 0 | 0.0% | 2 | 15.4% | 18 | 10.2% | 27 | 9.3% |

**Table 1.B** *continued.*

| | Competition levels | | | | | | | | | |
|---|---|---|---|---|---|---|---|---|---|---|
| | Continent Cups (n=197) | | World Cup (n=198) | | Master competitions (n=26) | | Mass events (n=20) | | Snow sports not governed by FIS (n=53) | |
| | n | % | n | % | n | % | n | % | n | % |
| **C. Training methods** | | | | | | | | | | |
| I don't know | 9 | 5.2% | 10 | 5.7% | 0 | 0.0% | 1 | 6.3% | 1 | 2.4% |
| Not at all | 30 | 17.3% | 36 | 20.6% | 1 | 5.0% | 2 | 12.5% | 8 | 19.5% |
| Slightly | 22 | 12.7% | 25 | 14.3% | 2 | 10.0% | 5 | 31.3% | 5 | 12.2% |
| Somewhat | 40 | 23.1% | 32 | 18.3% | 4 | 20.0% | 2 | 12.5% | 8 | 19.5% |
| Fairly | 32 | 18.5% | 29 | 16.6% | 4 | 20.0% | 2 | 12.5% | 7 | 17.1% |
| Very | 26 | 15.0% | 25 | 14.3% | 4 | 20.0% | 2 | 12.5% | 8 | 19.5% |
| Completely | 14 | 8.1% | 18 | 10.3% | 5 | 25.0% | 2 | 12.5% | 4 | 9.8% |
| **D. Testing practices** | | | | | | | | | | |
| I don't know | 25 | 14.5% | 22 | 12.6% | 5 | 25.0% | 0 | 0.0% | 5 | 12.2% |
| Not at all | 35 | 20.2% | 41 | 23.4% | 4 | 20.0% | 4 | 25.0% | 10 | 24.4% |
| Slightly | 19 | 11.0% | 20 | 11.4% | 2 | 10.0% | 2 | 12.5% | 6 | 14.6% |
| Somewhat | 34 | 19.7% | 32 | 18.3% | 4 | 20.0% | 1 | 6.3% | 7 | 17.1% |
| Fairly | 26 | 15.0% | 25 | 14.3% | 2 | 10.0% | 4 | 25.0% | 3 | 7.3% |
| Very | 23 | 13.3% | 23 | 13.1% | 1 | 5.0% | 3 | 18.8% | 5 | 12.2% |
| Completely | 11 | 6.4% | 12 | 6.9% | 2 | 10.0% | 2 | 12.5% | 5 | 12.2% |

**Table 1.C** Needs for more information on specific intervention areas: the return-to-sport process.

| | **Total** | | **Snow sports** | | | | | | | | | | | |
|---|---|---|---|---|---|---|---|---|---|---|---|---|---|---|
| | | | **Alpine Skiing (n=205)** | | **Freestyle Skiing (n=93)** | | **Snowboarding (n=72)** | | **Cross-Country Skiing (n=85)** | | **Ski Jumping (n=52)** | | **Nordic Combined (n=31)** | |
| | **n** | **%** | **n** | **%** | **n** | **%** | **n** | **%** | **n** | **%** | **n** | **%** | **n** | **%** |
| **E. Return-to-sport process** | | | | | | | | | | | | | | |
| I don't know | **41** | **10.0%** | 13 | 7.4% | 9 | 11.3% | 5 | 8.6% | 7 | 9.2% | 4 | 8.0% | 3 | 10.3% |
| Not at all | **60** | **14.6%** | 22 | 12.6% | 18 | 22.5% | 8 | 13.8% | 14 | 18.4% | 4 | 8.0% | 2 | 6.9% |
| Slightly | **47** | **11.5%** | 19 | 10.9% | 9 | 11.3% | 7 | 12.1% | 16 | 21.1% | 8 | 16.0% | 5 | 17.2% |
| Somewhat | **71** | **17.3%** | 34 | 19.4% | 13 | 16.3% | 11 | 19.0% | 9 | 11.8% | 8 | 16.0% | 6 | 20.7% |
| Fairly | **61** | **14.9%** | 21 | 12.0% | 12 | 15.0% | 8 | 13.8% | 10 | 13.2% | 11 | 22.0% | 8 | 27.6% |
| Very | **76** | **18.5%** | 37 | 21.1% | 12 | 15.0% | 7 | 12.1% | 12 | 15.8% | 10 | 20.0% | 5 | 17.2% |
| Completely | **54** | **13.2%** | 29 | 16.6% | 7 | 8.8% | 12 | 20.7% | 8 | 10.5% | 5 | 10.0% | 0 | 0.0% |

**Table 1.C** *continued.*

| | Stakeholder roles | | | | | | | | | | Competition levels | | | |
|---|---|---|---|---|---|---|---|---|---|---|---|---|---|---|
| | Athletes (n=261) | | Coaches (n=116) | | Team staff (n=68) | | Industry - SRS (n=5) | | FIS representatives (n=13) | | Youth competitions (n=205) | | FIS competitions (n=327) | |
| | n | % | n | % | n | % | n | % | n | % | n | % | n | % |
| **E. Return-to-sport process** | | | | | | | | | | | | | | |
| I don't know | 37 | 14.8% | 3 | 2.9% | 1 | 1.7% | 1 | 20.0% | 0 | 0.0% | 11 | 6.2% | 28 | 9.7% |
| Not at all | 37 | 14.8% | 15 | 14.3% | 10 | 16.7% | 0 | 0.0% | 4 | 30.8% | 24 | 13.5% | 35 | 12.1% |
| Slightly | 27 | 10.8% | 9 | 8.6% | 11 | 18.3% | 1 | 20.0% | 1 | 7.7% | 21 | 11.8% | 30 | 10.3% |
| Somewhat | 44 | 17.6% | 20 | 19.0% | 8 | 13.3% | 1 | 20.0% | 0 | 0.0% | 30 | 16.9% | 49 | 16.9% |
| Fairly | 31 | 12.4% | 20 | 19.0% | 13 | 21.7% | 0 | 0.0% | 2 | 15.4% | 21 | 11.8% | 46 | 15.9% |
| Very | 40 | 16.0% | 24 | 22.9% | 9 | 15.0% | 2 | 40.0% | 4 | 30.8% | 45 | 25.3% | 60 | 20.7% |
| Completely | 34 | 13.6% | 14 | 13.3% | 8 | 13.3% | 0 | 0.0% | 2 | 15.4% | 26 | 14.6% | 42 | 14.5% |

**Table 1.C** *continued.*

| | Competition levels | | | | | | | | | |
|---|---|---|---|---|---|---|---|---|---|---|
| | **Continent Cups (n=197)** | | **World Cup (n=198)** | | **Master competitions (n=26)** | | **Mass events (n=20)** | | **Snow sports not governed by FIS (n=53)** | |
| | **n** | **%** | **n** | **%** | **n** | **%** | **n** | **%** | **n** | **%** |
| **E. Return-to-sport process** | | | | | | | | | | |
| I don't know | 17 | 9.8% | 18 | 10.3% | 1 | 5.0% | 0 | 0.0% | 2 | 4.9% |
| Not at all | 26 | 15.0% | 33 | 19.0% | 2 | 10.0% | 0 | 0.0% | 7 | 17.1% |
| Slightly | 19 | 11.0% | 23 | 13.2% | 2 | 10.0% | 6 | 37.5% | 4 | 9.8% |
| Somewhat | 33 | 19.1% | 29 | 16.7% | 6 | 30.0% | 2 | 12.5% | 7 | 17.1% |
| Fairly | 27 | 15.6% | 30 | 17.2% | 0 | 0.0% | 1 | 6.3% | 6 | 14.6% |
| Very | 32 | 18.5% | 27 | 15.5% | 3 | 15.0% | 6 | 37.5% | 9 | 22.0% |
| Completely | 19 | 11.0% | 14 | 8.0% | 6 | 30.0% | 1 | 6.3% | 6 | 14.6% |

**Supplementary file 12**

**Table 1.A** Responsibilities across prevention stages: development and dissemination.

| | **Total** | | **Snow sports** | | | | | | | | | | | |
|---|---|---|---|---|---|---|---|---|---|---|---|---|---|---|
| | | | **Alpine Skiing (n=205)** | | **Freestyle Skiing (n=93)** | | **Snowboarding (n=72)** | | **Cross-Country Skiing (n=85)** | | **Ski Jumping (n=52)** | | **Nordic Combined (n=31)** | |
| | **n** | **%** | **n** | **%** | **n** | **%** | **n** | **%** | **n** | **%** | **n** | **%** | **n** | **%** |
| **A. Development** | | | | | | | | | | | | | | |
| Athlete | **182** | **41.7%** | 69 | 33.7% | 35 | 37.6% | 22 | 30.6% | 28 | 32.9% | 31 | 59.6% | 12 | 38.7% |
| Coach | **273** | **62.6%** | 119 | 58.0% | 54 | 58.1% | 35 | 48.6% | 40 | 47.1% | 38 | 73.1% | 17 | 54.8% |
| Team staff | **249** | **57.1%** | 119 | 58.0% | 56 | 60.2% | 42 | 58.3% | 46 | 54.1% | 14 | 26.9% | 19 | 61.3% |
| SRS/industry | **96** | **22.0%** | 57 | 27.8% | 19 | 20.4% | 11 | 15.3% | 12 | 14.1% | 5 | 9.6% | 7 | 22.6% |
| Regional/NSSA | **185** | **42.4%** | 100 | 48.8% | 32 | 34.4% | 32 | 44.4% | 32 | 37.6% | 13 | 25.0% | 11 | 35.5% |
| FIS | **231** | **53.0%** | 116 | 56.6% | 47 | 50.5% | 34 | 47.2% | 31 | 36.5% | 21 | 40.4% | 18 | 58.1% |
| Others | **11** | **2.5%** | 6 | 2.9% | 3 | 3.2% | 2 | 2.8% | 1 | 1.2% | 0 | 0.0% | 1 | 3.2% |
| **B. Dissemination** | | | | | | | | | | | | | | |
| Athlete | **155** | **35.6%** | 59 | 28.8% | 25 | 26.9% | 25 | 34.7% | 28 | 32.9% | 29 | 55.8% | 12 | 38.7% |
| Coach | **286** | **65.6%** | 121 | 59.0% | 59 | 63.4% | 51 | 70.8% | 46 | 54.1% | 37 | 71.2% | 23 | 74.2% |
| Team staff | **211** | **48.4%** | 102 | 49.8% | 47 | 50.5% | 39 | 54.2% | 32 | 37.6% | 17 | 32.7% | 13 | 41.9% |
| SRS/industry | **102** | **23.4%** | 60 | 29.3% | 15 | 16.1% | 18 | 25.0% | 12 | 14.1% | 7 | 13.5% | 6 | 19.4% |

| | | | | | | | | | | | | | | |
|---|---|---|---|---|---|---|---|---|---|---|---|---|---|---|
| Regional/NSSA | **241** | **55.3%** | 118 | 57.6% | 43 | 46.2% | 39 | 54.2% | 41 | 48.2% | 21 | 40.4% | 20 | 64.5% |
| FIS | **248** | **56.9%** | 125 | 61.0% | 49 | 52.7% | 32 | 44.4% | 42 | 49.4% | 21 | 40.4% | 19 | 61.3% |
| Others | **15** | **3.4%** | 10 | 4.9% | 3 | 3.2% | 4 | 5.6% | 2 | 2.4% | 0 | 0.0% | 0 | 0.0% |

**Table 1.A** *continued.*

| | **Stakeholder roles** | | | | | | | | | | **Competition levels** | | | |
|---|---|---|---|---|---|---|---|---|---|---|---|---|---|---|
| | **Athletes (n=261)** | | **Coaches (n=116)** | | **Team staff (n=68)** | | **Industry - SRS (n=5)** | | **FIS representatives (n=13)** | | **Youth competitions (n=205)** | | **FIS competitions (n=327)** | |
| | **n** | **%** | **n** | **%** | **n** | **%** | **n** | **%** | **n** | **%** | **n** | **%** | **n** | **%** |
| **A. Development** | | | | | | | | | | | | | | |
| Athlete | 128 | 49.0% | 40 | 34.5% | 19 | 27.9% | 1 | 20.0% | 6 | 46.2% | 74 | 36.1% | 131 | 40.1% |
| Coach | 167 | 64.0% | 76 | 65.5% | 34 | 50.0% | 2 | 40.0% | 12 | 92.3% | 118 | 57.6% | 198 | 60.6% |
| Team staff | 137 | 52.5% | 75 | 64.7% | 46 | 67.6% | 2 | 40.0% | 9 | 69.2% | 105 | 51.2% | 175 | 53.5% |
| SRS/industry | 49 | 18.8% | 33 | 28.4% | 17 | 25.0% | 2 | 40.0% | 3 | 23.1% | 32 | 15.6% | 62 | 19.0% |
| Regional/NSSA | 89 | 34.1% | 70 | 60.3% | 35 | 51.5% | 0 | 0.0% | 6 | 46.2% | 89 | 43.4% | 131 | 40.1% |
| FIS | 125 | 47.9% | 76 | 65.5% | 34 | 50.0% | 5 | 100.0% | 6 | 46.2% | 94 | 45.9% | 166 | 50.8% |
| Others | 5 | 1.9% | 4 | 3.4% | 3 | 4.4% | 0 | 0.0% | 0 | 0.0% | 9 | 4.4% | 5 | 1.5% |
| **B. Dissemination** | | | | | | | | | | | | | | |
| Athlete | 103 | 39.5% | 34 | 29.3% | 23 | 33.8% | 0 | 0.0% | 5 | 38.5% | 71 | 34.6% | 112 | 34.3% |
| Coach | 171 | 65.5% | 78 | 67.2% | 44 | 64.7% | 2 | 40.0% | 9 | 69.2% | 131 | 63.9% | 206 | 63.0% |
| Team staff | 113 | 43.3% | 63 | 54.3% | 41 | 60.3% | 1 | 20.0% | 7 | 53.8% | 86 | 42.0% | 145 | 44.3% |
| SRS/industry | 50 | 19.2% | 37 | 31.9% | 20 | 29.4% | 0 | 0.0% | 1 | 7.7% | 43 | 21.0% | 66 | 20.2% |
| Regional/NSSA | 126 | 48.3% | 79 | 68.1% | 45 | 66.2% | 1 | 20.0% | 9 | 69.2% | 113 | 55.1% | 172 | 52.6% |
| FIS | 134 | 51.3% | 77 | 66.4% | 40 | 58.8% | 5 | 100.0% | 6 | 46.2% | 99 | 48.3% | 177 | 54.1% |
| Others | 3 | 1.1% | 7 | 6.0% | 6 | 8.8% | 0 | 0.0% | 0 | 0.0% | 12 | 5.9% | 7 | 2.1% |

**Table 1.A** *continued.*

| | **Competition levels** | | | | | | | | | |
|---|---|---|---|---|---|---|---|---|---|---|
| | **Continent Cups (n=197)** | | **World Cup (n=198)** | | **Master competitions (n=26)** | | **Mass events (n=20)** | | **Snow sports not governed by FIS (n=53)** | |
| | **n** | **%** | **n** | **%** | **n** | **%** | **n** | **%** | **n** | **%** |
| **A. Development** | | | | | | | | | | |
| Athlete | 72 | 36.5% | 78 | 39.4% | 10 | 38.5% | 10 | 50.0% | 21 | 39.6% |
| Coach | 118 | 59.9% | 110 | 55.6% | 14 | 53.8% | 12 | 60.0% | 26 | 49.1% |
| Team staff | 115 | 58.4% | 124 | 62.6% | 12 | 46.2% | 7 | 35.0% | 29 | 54.7% |
| SRS/industry | 45 | 22.8% | 50 | 25.3% | 8 | 30.8% | 2 | 10.0% | 12 | 22.6% |
| Regional/NSSA | 82 | 41.6% | 74 | 37.4% | 14 | 53.8% | 9 | 45.0% | 26 | 49.1% |
| FIS | 113 | 57.4% | 114 | 57.6% | 13 | 50.0% | 6 | 30.0% | 22 | 41.5% |
| Others | 3 | 1.5% | 4 | 2.0% | 2 | 7.7% | 0 | 0.0% | 3 | 5.7% |
| **B. Dissemination** | | | | | | | | | | |
| Athlete | 62 | 31.5% | 71 | 35.9% | 3 | 11.5% | 6 | 30.0% | 22 | 41.5% |
| Coach | 119 | 60.4% | 116 | 58.6% | 15 | 57.7% | 12 | 60.0% | 31 | 58.5% |
| Team staff | 94 | 47.7% | 98 | 49.5% | 11 | 42.3% | 8 | 40.0% | 26 | 49.1% |
| SRS/industry | 42 | 21.3% | 45 | 22.7% | 7 | 26.9% | 3 | 15.0% | 14 | 26.4% |
| Regional/NSSA | 109 | 55.3% | 99 | 50.0% | 16 | 61.5% | 14 | 70.0% | 29 | 54.7% |
| FIS | 117 | 59.4% | 122 | 61.6% | 12 | 46.2% | 8 | 40.0% | 26 | 49.1% |
| Others | 3 | 1.5% | 5 | 2.5% | 2 | 7.7% | 0 | 0.0% | 4 | 7.5% |

**Table 1.B** Responsibilities across prevention stages: implementation.

| | Total | | Snow sports | | | | | | | | | | | |
|---|---|---|---|---|---|---|---|---|---|---|---|---|---|---|
| | | | Alpine Skiing (n=205) | | Freestyle Skiing (n=93) | | Snowboarding (n=72) | | Cross-Country Skiing (n=85) | | Ski Jumping (n=52) | | Nordic Combined (n=31) | |
| | n | % | n | % | n | % | n | % | n | % | n | % | n | % |
| **C. Implementation** | | | | | | | | | | | | | | |
| Athlete | **337** | **77.3%** | 145 | 70.7% | 60 | 64.5% | 52 | 72.2% | 53 | 62.4% | 45 | 86.5% | 25 | 80.6% |
| Coach | **356** | **81.7%** | 158 | 77.1% | 70 | 75.3% | 54 | 75.0% | 62 | 72.9% | 40 | 76.9% | 27 | 87.1% |
| Team staff | **271** | **62.2%** | 127 | 62.0% | 57 | 61.3% | 45 | 62.5% | 50 | 58.8% | 19 | 36.5% | 22 | 71.0% |
| SRS/industry | **120** | **27.5%** | 65 | 31.7% | 22 | 23.7% | 19 | 26.4% | 19 | 22.4% | 12 | 23.1% | 13 | 41.9% |
| Regional/NSSA | **165** | **37.8%** | 77 | 37.6% | 36 | 38.7% | 31 | 43.1% | 27 | 31.8% | 17 | 32.7% | 20 | 64.5% |
| FIS | **210** | **48.2%** | 102 | 49.8% | 43 | 46.2% | 36 | 50.0% | 26 | 30.6% | 23 | 44.2% | 22 | 71.0% |
| Others | **18** | **4.1%** | 13 | 6.3% | 2 | 2.2% | 3 | 4.2% | 3 | 3.5% | 2 | 3.8% | 2 | 6.5% |

**Table 1.B** *continued.*

| | **Stakeholder roles** | | | | | | | | | | **Competition levels** | | | |
|---|---|---|---|---|---|---|---|---|---|---|---|---|---|---|
| | **Athletes (n=261)** | | **Coaches (n=116)** | | **Team staff (n=68)** | | **Industry - SRS (n=5)** | | **FIS representatives (n=13)** | | **Youth competitions (n=205)** | | **FIS competitions (n=327)** | |
| | **n** | **%** | **n** | **%** | **n** | **%** | **n** | **%** | **n** | **%** | **n** | **%** | **n** | **%** |
| **C. Implementation** | | | | | | | | | | | | | | |
| Athlete | 214 | 82.0% | 88 | 75.9% | 45 | 66.2% | 2 | 40.0% | 9 | 69.2% | 144 | 70.2% | 232 | 70.9% |
| Coach | 203 | 77.8% | 109 | 94.0% | 54 | 79.4% | 2 | 40.0% | 11 | 84.6% | 158 | 77.1% | 252 | 77.1% |
| Team staff | 146 | 55.9% | 83 | 71.6% | 51 | 75.0% | 3 | 60.0% | 9 | 69.2% | 113 | 55.1% | 189 | 57.8% |
| SRS/industry | 53 | 20.3% | 47 | 40.5% | 26 | 38.2% | 3 | 60.0% | 2 | 15.4% | 48 | 23.4% | 81 | 24.8% |
| Regional/NSSA | 74 | 28.4% | 64 | 55.2% | 36 | 52.9% | 1 | 20.0% | 7 | 53.8% | 77 | 37.6% | 119 | 36.4% |
| FIS | 104 | 39.8% | 75 | 64.7% | 37 | 54.4% | 4 | 80.0% | 5 | 38.5% | 85 | 41.5% | 147 | 45.0% |
| Others | 8 | 3.1% | 6 | 5.2% | 5 | 7.4% | 0 | 0.0% | 1 | 7.7% | 10 | 4.9% | 8 | 2.4% |

**Table 1.B** *continued.*

| | Competition levels | | | | | | | | | |
|---|---|---|---|---|---|---|---|---|---|---|
| | **Continent Cups (n=197)** | | **World Cup (n=198)** | | **Master competitions (n=26)** | | **Mass events (n=20)** | | **Snow sports not governed by FIS (n=53)** | |
| | **n** | **%** | **n** | **%** | **n** | **%** | **n** | **%** | **n** | **%** |
| **C. Implementation** | | | | | | | | | | |
| Athlete | 131 | 66.5% | 138 | 69.7% | 17 | 65.4% | 11 | 55.0% | 36 | 67.9% |
| Coach | 155 | 78.7% | 146 | 73.7% | 15 | 57.7% | 14 | 70.0% | 41 | 77.4% |
| Team staff | 128 | 65.0% | 137 | 69.2% | 14 | 53.8% | 11 | 55.0% | 32 | 60.4% |
| SRS/industry | 56 | 28.4% | 69 | 34.8% | 9 | 34.6% | 6 | 30.0% | 17 | 32.1% |
| Regional/NSSA | 82 | 41.6% | 82 | 41.4% | 10 | 38.5% | 10 | 50.0% | 23 | 43.4% |
| FIS | 107 | 54.3% | 116 | 58.6% | 10 | 38.5% | 8 | 40.0% | 26 | 49.1% |
| Others | 4 | 2.0% | 5 | 2.5% | 4 | 15.4% | 0 | 0.0% | 1 | 1.9% |

**Supplementary file 13**

**Table 1** Knowledge dissemination preferences.

| **Communication tool** | **Total** | | **Snow sport** | | | | | | | | | | | |
|---|---|---|---|---|---|---|---|---|---|---|---|---|---|---|
| | | | **Alpine Skiing (n=205)** | | **Freestyle Skiing (n=93)** | | **Snowboarding (n=72)** | | **Cross-Country Skiing (n=85)** | | **Ski Jumping (n=52)** | | **Nordic Combined (n=31)** | |
| | **n** | **%** | **n** | **%** | **n** | **%** | **n** | **%** | **n** | **%** | **n** | **%** | **n** | **%** |
| Book | **88** | **20.2%** | 44 | 21.5% | 14 | 15.1% | 14 | 19.4% | 16 | 18.8% | 9 | 17.3% | 7 | 22.6% |
| e-Book | **132** | **30.3%** | 66 | 32.2% | 25 | 26.9% | 21 | 29.2% | 23 | 27.1% | 12 | 23.1% | 9 | 29.0% |
| Magazine print | **57** | **13.1%** | 34 | 16.6% | 11 | 11.8% | 5 | 6.9% | 10 | 11.8% | 2 | 3.8% | 4 | 12.9% |
| Magazine digital | **77** | **17.7%** | 41 | 20.0% | 16 | 17.2% | 8 | 11.1% | 12 | 14.1% | 11 | 21.2% | 4 | 12.9% |
| Newsletter print | **35** | **8.0%** | 21 | 10.2% | 9 | 9.7% | 5 | 6.9% | 5 | 5.9% | 2 | 3.8% | 3 | 9.7% |
| Newsletter e-mail | **137** | **31.4%** | 72 | 35.1% | 23 | 24.7% | 22 | 30.6% | 29 | 34.1% | 12 | 23.1% | 9 | 29.0% |
| Podcasts audio | **151** | **34.6%** | 73 | 35.6% | 34 | 36.6% | 22 | 30.6% | 27 | 31.8% | 11 | 21.2% | 10 | 32.3% |
| Podcasts video | **140** | **32.1%** | 74 | 36.1% | 33 | 35.5% | 20 | 27.8% | 21 | 24.7% | 10 | 19.2% | 6 | 19.4% |
| Seminars | **119** | **27.3%** | 61 | 29.8% | 19 | 20.4% | 18 | 25.0% | 21 | 24.7% | 17 | 32.7% | 11 | 35.5% |
| Webinars | **128** | **29.4%** | 67 | 32.7% | 25 | 26.9% | 23 | 31.9% | 27 | 31.8% | 18 | 34.6% | 11 | 35.5% |
| Weblog | **19** | **4.4%** | 7 | 3.4% | 5 | 5.4% | 6 | 8.3% | 4 | 4.7% | 4 | 7.7% | 4 | 12.9% |
| Website | **154** | **35.3%** | 77 | 37.6% | 22 | 23.7% | 18 | 25.0% | 31 | 36.5% | 23 | 44.2% | 11 | 35.5% |
| Social media | **125** | **28.7%** | 49 | 23.9% | 26 | 28.0% | 17 | 23.6% | 28 | 32.9% | 14 | 26.9% | 4 | 12.9% |
| Testimonials personal | **64** | **14.7%** | 33 | 16.1% | 10 | 10.8% | 10 | 13.9% | 16 | 18.8% | 8 | 15.4% | 5 | 16.1% |

| | | | | | | | | | | | | | | |
|---|---|---|---|---|---|---|---|---|---|---|---|---|---|---|
| Testimonials digital | **49** | **11.2%** | 34 | 16.6% | 11 | 11.8% | 7 | 9.7% | 10 | 11.8% | 2 | 3.8% | 1 | 3.2% |
| Other/s | **10** | **2.3%** | 3 | 1.5% | 5 | 5.4% | 3 | 4.2% | 1 | 1.2% | 1 | 1.9% | 0 | 0.0% |

**Table 1** *continued.*

| Communication tool | Stakeholder roles | | | | | | | | | | Competition levels | | | |
|---|---|---|---|---|---|---|---|---|---|---|---|---|---|---|
| | Athletes (n=261) | | Coaches (n=116) | | Team staff (n=68) | | Industry - SRS (n=5) | | FIS representatives (n=13) | | Youth competitions (n=205) | | FIS competitions (n=327) | |
| | n | % | n | % | n | % | n | % | n | % | n | % | n | % |
| Book | 48 | 18.4% | 26 | 22.4% | 19 | 27.9% | 2 | 40.0% | 3 | 23.1% | 36 | 17.6% | 61 | 18.7% |
| e-Book | 74 | 28.4% | 39 | 33.6% | 23 | 33.8% | 2 | 40.0% | 8 | 61.5% | 54 | 26.3% | 93 | 28.4% |
| Magazine print | 39 | 14.9% | 12 | 10.3% | 7 | 10.3% | 0 | 0.0% | 3 | 23.1% | 24 | 11.7% | 40 | 12.2% |
| Magazine digital | 50 | 19.2% | 15 | 12.9% | 10 | 14.7% | 2 | 40.0% | 7 | 53.8% | 29 | 14.1% | 58 | 17.7% |
| Newsletter print | 20 | 7.7% | 10 | 8.6% | 4 | 5.9% | 1 | 20.0% | 3 | 23.1% | 13 | 6.3% | 26 | 8.0% |
| Newsletter e-mail | 73 | 28.0% | 39 | 33.6% | 22 | 32.4% | 3 | 60.0% | 7 | 53.8% | 71 | 34.6% | 107 | 32.7% |
| Podcasts audio | 98 | 37.5% | 37 | 31.9% | 18 | 26.5% | 1 | 20.0% | 5 | 38.5% | 62 | 30.2% | 108 | 33.0% |
| Podcasts video | 80 | 30.7% | 43 | 37.1% | 16 | 23.5% | 3 | 60.0% | 4 | 30.8% | 64 | 31.2% | 104 | 31.8% |
| Seminars | 51 | 19.5% | 47 | 40.5% | 27 | 39.7% | 0 | 0.0% | 7 | 53.8% | 55 | 26.8% | 92 | 28.1% |
| Webinars | 47 | 18.0% | 52 | 44.8% | 37 | 54.4% | 1 | 20.0% | 6 | 46.2% | 65 | 31.7% | 94 | 28.7% |
| Weblog | 11 | 4.2% | 4 | 3.4% | 4 | 5.9% | 0 | 0.0% | 1 | 7.7% | 7 | 3.4% | 14 | 4.3% |
| Website | 101 | 38.7% | 35 | 30.2% | 22 | 32.4% | 3 | 60.0% | 3 | 23.1% | 65 | 31.7% | 119 | 36.4% |
| Social media | 104 | 39.8% | 13 | 11.2% | 9 | 13.2% | 0 | 0.0% | 3 | 23.1% | 55 | 26.8% | 99 | 30.3% |
| Testimonials personal | 41 | 15.7% | 14 | 12.1% | 12 | 17.6% | 0 | 0.0% | 2 | 15.4% | 27 | 13.2% | 49 | 15.0% |
| Testimonials digital | 31 | 11.9% | 12 | 10.3% | 8 | 11.8% | 1 | 20.0% | 2 | 15.4% | 22 | 10.7% | 38 | 11.6% |
| Other/s | 4 | 1.5% | 4 | 3.4% | 3 | 4.4% | 0 | 0.0% | 0 | 0.0% | 4 | 2.0% | 5 | 1.5% |

**Table 1** *continued.*

| Communication tool | Competition levels | | | | | | | | | |
|---|---|---|---|---|---|---|---|---|---|---|
| | Continent Cups (n=197) | | World Cup (n=198) | | Master competitions (n=26) | | Mass events (n=20) | | Snow sports not governed by FIS (n=53) | |
| | n | % | n | % | n | % | n | % | n | % |
| Book | 38 | 19.3% | 38 | 19.2% | 4 | 15.4% | 4 | 20.0% | 10 | 18.9% |
| e-Book | 56 | 28.4% | 60 | 30.3% | 7 | 26.9% | 6 | 30.0% | 15 | 28.3% |
| Magazine print | 20 | 10.2% | 19 | 9.6% | 6 | 23.1% | 2 | 10.0% | 4 | 7.5% |
| Magazine digital | 34 | 17.3% | 27 | 13.6% | 5 | 19.2% | 4 | 20.0% | 6 | 11.3% |
| Newsletter print | 15 | 7.6% | 13 | 6.6% | 5 | 19.2% | 1 | 5.0% | 6 | 11.3% |
| Newsletter e-mail | 66 | 33.5% | 48 | 24.2% | 12 | 46.2% | 8 | 40.0% | 19 | 35.8% |
| Podcasts audio | 74 | 37.6% | 62 | 31.3% | 7 | 26.9% | 7 | 35.0% | 14 | 26.4% |
| Podcasts video | 68 | 34.5% | 54 | 27.3% | 8 | 30.8% | 4 | 20.0% | 12 | 22.6% |
| Seminars | 55 | 27.9% | 55 | 27.8% | 7 | 26.9% | 8 | 40.0% | 13 | 24.5% |
| Webinars | 65 | 33.0% | 63 | 31.8% | 9 | 34.6% | 9 | 45.0% | 20 | 37.7% |
| Weblog | 9 | 4.6% | 7 | 3.5% | 2 | 7.7% | 2 | 10.0% | 3 | 5.7% |
| Website | 67 | 34.0% | 59 | 29.8% | 12 | 46.2% | 7 | 35.0% | 10 | 18.9% |
| Social media | 57 | 28.9% | 36 | 18.2% | 2 | 7.7% | 4 | 20.0% | 7 | 13.2% |
| Testimonials personal | 26 | 13.2% | 29 | 14.6% | 3 | 11.5% | 1 | 5.0% | 9 | 17.0% |
| Testimonials digital | 24 | 12.2% | 23 | 11.6% | 3 | 11.5% | 1 | 5.0% | 8 | 15.1% |
| Other/s | 4 | 2.0% | 7 | 3.5% | 1 | 3.8% | 0 | 0.0% | 3 | 5.7% |
